# Production of white cheese using the gel brine technique and determination of its physicochemical, color, and microbiological properties using multivariate statistical analyses

**DOI:** 10.3389/fnut.2026.1930095

**Published:** 2026-10-21

**Authors:** Mustafa Çavuş, Hasan Yetim, Hasan Cankurt

**Affiliations:** 1 Food Technology Program, Department of Food Processing, Safiye Çıkrıkçıoğlu Vocational School, Kayseri University, Kayseri, Türkiye; 2 Department of Food Engineering, Faculty of Engineering and Natural Sciences, Istanbul Sabahattin Zaim University, Istanbul, Türkiye; 3 Department of Food Engineering, Faculty of Chemistry and Metallurgy, Yıldız Technical University, Istanbul, Türkiye; 4 Faculty of Health Sciences, Department of Nutrition and Dietetics, Biruni University, Istanbul, Türkiye

**Keywords:** gel brine, salt, stabilizer, storage, white cheese

## Abstract

**Background:**

In this research, a new salting method called the “gel brine technique” was developed and applied in cheese salting. The gel brine technique involves increasing the viscosity of the brine used in cheese salting by adding certain thickening and gelling stabilizers.

**Methods:**

The aim was to reduce the salt content of brined white cheese, which holds an important place in the human diet due to its nutritional components. In the study, white cheese brine was prepared in gel form by adding different stabilizers (gelatin, xanthan gum, carrageenan gum, and pectin). The stabilizers were used at the following concentrations: carrageenan gum and xanthan gum at 0.5, 1.0, 1.5%; pectin and gelatin at 1.0, 2.0, 3.0%; and salt at 2.0, 4.0, and 6.0%. A control group consisting of brine without stabilizers but containing salt at 2.0, 4.0, and 6.0% was also included. The white cheeses were then kept in these brines for 90 days and stored at +4 °C. The physicochemical, color and microbiological properties of the cheeses produced in duplicate were analyzed.

**Results:**

As a result, the dry matter content of cheeses produced with gel brine ranged from 28.26 to 44.81%, salt content from 0.79 to 3.45%, water activity (a_w_) from 0.92 to 0.93, and L* values (color parameter) from 71.82 to 94.59. For microbiological counts, total yeast-mold counts ranged from 1.0 to 6.7 log cfu/g. Since the gel brines in this study were prepared with very low salt levels, some cheeses did not survive until the end of the storage period and exhibited melting. For example, the control group samples melted completely, so analyses on days 60 and 90 could not be performed. However, some samples survived the storage period without melting even at 2% salt and were also sensorially appreciated (the cheese with 3% gelatin and 2% salt in its brine, and the cheese with 1% carrageenan gum and 2% salt in its brine).

**Conclusion:**

The study concluded that low-salt, uniquely flavored white cheeses that do not melt in their packaging and retain their texture can be successfully produced using the gel brine technique.

## 1 Introduction

When cheeses are kept in high-salt brine, they become excessively salty, and the cheese’s unique flavor is lost. Only the taste of salt is perceived. In addition, the excessive salt suppresses the starter culture that creates the aroma, and the desired flavor and aroma of the cheese are weakened. The portion of the cheese remaining on the surface of the brine loses moisture, and mold can form within a few days. The “gel brine technique” is a method used to solve these problems (1). Gel brine is prepared by dissolving a stabilizer and salt in potable water, then pasteurizing at 85 °C for 15 min and cooling to 30–35 °C. The prepared gel brines are then transferred to containers holding the cheeses. Limiting the transfer of salt and water between the gel brine and the cheese allows for the use of lower salt concentrations. Since its introduction by Cankurt (2), the gel brine technique has been extended to soft white cheese (3) and to raw milk cheese (4).

It is well known that the most commonly used preservation method for white cheese is keeping it in brine containing 12–14% salt. White cheese, which has a white color, a non-porous structure, and a mildly salty acidic taste, is cut into standard sizes of 7 × 7 × 7 or 7 × 7 × 10 cm and weighed 350–500 g, then left to ripen in brine (5, 6). The structure of white cheese has been classified in various ways in the literature (semi-hard, soft, semi-soft). Freshly produced white cheese has a soft structure, but after a 90-day ripening period, it is classified as semi-hard or semi-soft (7). Despite its popularity, traditional immersion in high-concentration brine often results in excessive salt uptake and potential structural degradation over prolonged storage periods (4). To mitigate these challenges, the gel brine technique leverages stabilizers to modulate mass transfer kinetics, thereby facilitating a controlled reduction in sodium levels while maintaining the structural integrity of the cheese matrix (8). By regulating salt diffusion through a stabilized viscous medium, this approach offers a precise alternative to traditional salting, which often struggles to balance sensory quality with sodium reduction targets (9).

Salting, one of the oldest known food preservation methods, is performed on cheese either as dry salting or immersion in brine. Salt is also responsible for the characteristic aroma and good textural properties of white cheese (7). Moreover, salt directly affects the physicochemical and biochemical events occurring in cheese. Salt limits microbiological development, provides enzyme inactivation, decreases the moisture content of cheese (contributing to textural structure), and plays a role in enhancing product quality (10, 11). Despite these functional benefits, high sodium intake is a major public health concern, prompting investigations into innovative brining techniques to reduce salt concentrations without compromising food safety (12).

Although sodium is essential for cellular homeostasis, fluid and electrolyte balance, and neuromuscular function, the estimated physiological requirement is <500 mg/day. Nevertheless, global average daily sodium intake exceeds 4,000 mg, approximately twice the WHO-recommended limit of <2,000 mg/day for adults. Excessive sodium intake increases the risk of various chronic diseases, particularly cardiovascular disease, stroke, and coronary heart disease, and has also been associated with obesity, gastric cancer, and osteoporosis. Although 194 countries committed in 2013 to a 30% reduction in population sodium intake, this target has not yet been achieved. Effective implementation of WHO-recommended policies could reduce sodium intake by more than 20% and potentially prevent over 7 million deaths by 2030 (13). In line with this global target, Türkiye received a sodium reduction policy score of 3 out of 4, placing it among the countries in the European Region with relatively advanced sodium reduction policies. In Türkiye, sodium reduction has been incorporated into both the Noncommunicable Disease Plan and the multisectoral development plan. Mandatory measures include sodium declaration on pre-packaged foods, public food procurement policies in schools, and mandatory marketing restrictions on television and radio targeting children and adolescents up to 18 years of age. In addition, voluntary sodium reduction targets have been established for several food categories, including bread, cheese, processed meat, savoury snacks, and ready-to-eat foods. However, the absence of front-of-pack labelling and sodium-specific media campaigns prevents Türkiye from achieving the highest policy score of 4. Therefore, although Türkiye has made substantial progress in establishing and implementing sodium reduction policies, further strengthening of mandatory reformulation, front-of-pack labelling, and public awareness initiatives is needed to achieve greater reductions in population sodium intake. This situation necessitates not only a reduction in sodium consumption at the societal level but also the development of effective production and salting techniques aimed at reducing sodium content, particularly in dairy products. The primary goal in this process is to reduce sodium content while preserving the structural, sensory, and technological properties of the products (13, 14).

During the storage of cheese in brine, certain nutritional components diffuse into the brine solution. One of the primary objectives of this study was to reduce the migration of these components by converting the brine into a gel, thereby contributing to the preservation of the cheese’s nutritional quality. Furthermore, when cheeses are immersed in conventional brine, they tend to float to the surface due to the density of the brine. As a result, the portion of the cheese exposed above the brine loses moisture and may develop mold within a few days. In contrast, no such problem was observed in cheeses stored in gelled brine.

Accordingly, different salt concentrations and various stabilizers were employed in the production of White cheese. Throughout the storage period, physicochemical, color, and microbiological analyses were performed on the samples, and the obtained results were comparatively evaluated.

## 2 Materials and methods

### 2.1 Materials

Full-fat cow’s milk was used to produce the cheeses in the study. The milk was purchased from the dairy farm of Erciyes University Agricultural Research Center. The cows were of the Holstein breed, and the milk was obtained during the summer season in the third lactation period. A commercial freeze-dried direct-to-use cheese starter culture (Danisco Choozit RA 22, Denmark), calcium chloride, and microbial rennet (Fermento 220, Strength: 220 IMCU/ml, Konya) were used as a coagulant enzyme. The following stabilizers were used: bovine gelatin (Benosan 250 Bloom), xanthan gum, carrageenan gum, and pectin. Cheese production was carried out at the Pilot Application Center of the Food Technology Department at Kayseri University Safiye Çıkrıkçıoğlu Vocational School. Three kilograms of each sample was produced and placed in plastic (polypropylene) containers. In preparing the brine, ordinary tap water and refined sea salt containing Cl, Na, I, and other minerals were used. High-purity chemicals from Sigma-Aldrich and Merck were used in the analyses performed on the cheeses.

### 2.2 Methods

#### 2.2.1 Research design

The experimental cheeses were produced in duplicate. In this study, a 4 × 3 × 3 × 5 factorial design was used for stabilized cheeses. Factor A was gel brine application (4 levels: stabiliser-supplemented gel brine); Factor B was stabiliser concentration (3 levels: carrageenan gum and xanthan gum at 0.5, 1.0, 1.5%; gelatin and pectin at 1, 2, 3%); Factor C was brine salt concentration (3 levels: 2.0, 4.0, 6.0% w/w NaCl); and Factor D was storage duration at 4 °C (5 levels: 0, 15, 30, 60, 90 days). Three independent cheese-making trials were conducted, yielding a total of 36 treatment combinations × 5 storage times × 2 duplicates = 360 experimental units (Supplementary Table S1, *n* = 72).

The study employed a 1 × 1 × 3 × 5 factorial design for the control group. Factor A was the traditional brine application (1 level); Factor B was the stabilizer concentration (1 level: 0%); Factor C was the brine salt concentration (3 levels: 2.0, 4.0, 6.0% w/w NaCl); and Factor D was the storage time at 4 °C (5 levels: 0, 15, 30, 60, 90 days). Three independent cheese-making trials were conducted for the control group, yielding a total of 3 treatment combinations × 5 storage times × 2 duplicates = 30 experimental units (Supplementary Table S1, *n* = 6).

Some of the 78 (72 + 6) experimental cheeses did not survive until the end of the storage period and melted; since a valid comparison would not be possible, no measurements were taken on these cheeses.

#### 2.2.2 Preparation of white cheese samples

Full-fat cow’s milk intended for cheesemaking was subjected to platform tests. Once the milk was deemed suitable, it was processed. After filtration, the milk was pasteurized at 80 °C for 2 min in a steam-jacketed double-walled cooking kettle. It was then immediately cooled to the inoculation temperature of 37 °C, and a previously activated 2.0% direct-to-use starter culture and 1.0% calcium chloride were added. Based on the rennet test, the rennet amount was calculated, and coagulation was performed in a 500 kg chrome steel cheese vat at 37 °C using microbial rennet (Fermento 220, Strength: 220 IMCU/ml, Konya) over a 1-h coagulation period. At the end of this period, the curd was cut with a cheese harp and left to rest for 10 min. The whey was then drained. After being left to stand for half an hour, the curd was collected and pressed. After pressing for 2 h, it was cut into slices of 4 × 5 × 7 cm in size. These slices weighed 0.5 kg each and were placed into containers in 3 kg portions (15). Bovine gelatin (Benosan 250B) was used. After the gel and viscous brines were pasteurized at 85 °C for 15 min, they were allowed to cool (30–35 °C), and then 2 kg was placed in each plastic container on top of the experimental cheeses. The cheeses were stored throughout the storage period at +4 °C in transparent, wide-mouthed plastic containers (16). The experimental cheeses were produced in duplicate. Samples were analyzed on days 1, 15, 30, 60, and 90. Some of the experimental cheeses did not survive until the end of the storage period and melted; since a valid comparison would not be possible, no measurements were taken on these cheeses.

In the preliminary trials of the study, white cheese samples were produced with brines containing different stabilizers and salt at various ratios, as well as control cheeses whose brine contained only salt. Images related to the cheese production process are presented in Figures 1, 2.

**Figure 1:**
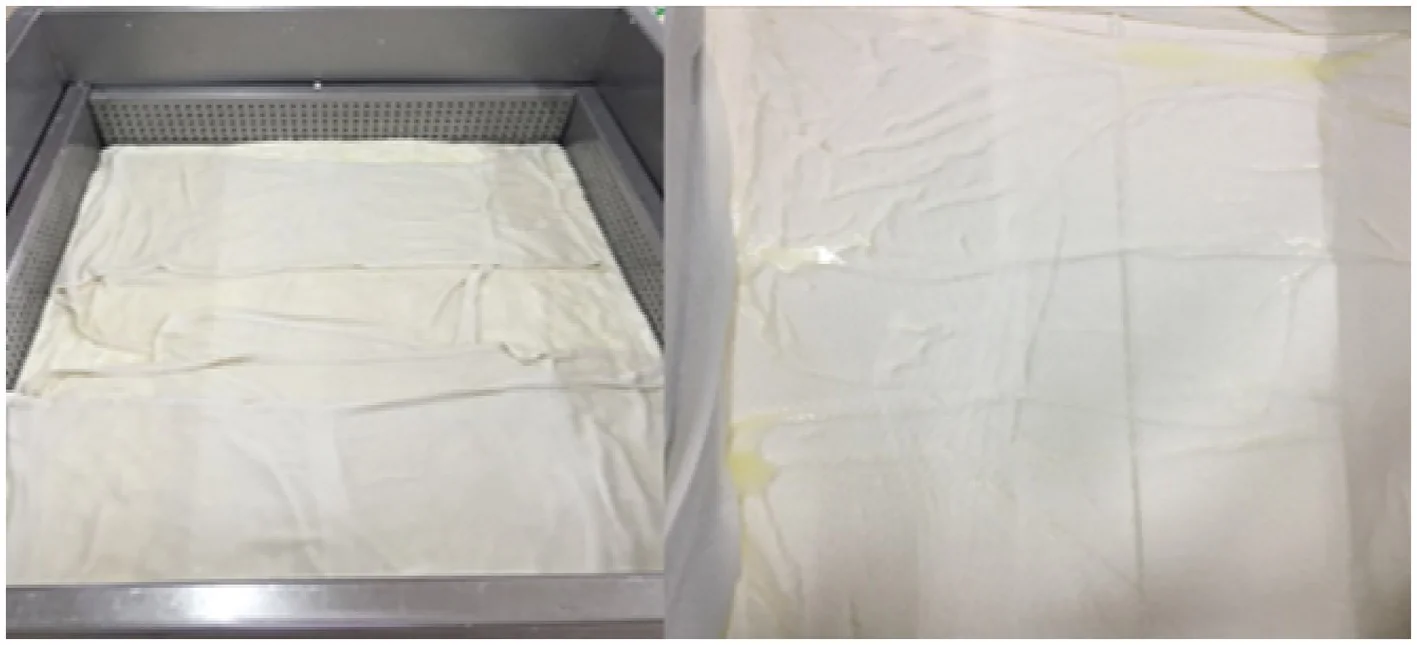
Production of brine-cured white cheese.

**Figure 2:**
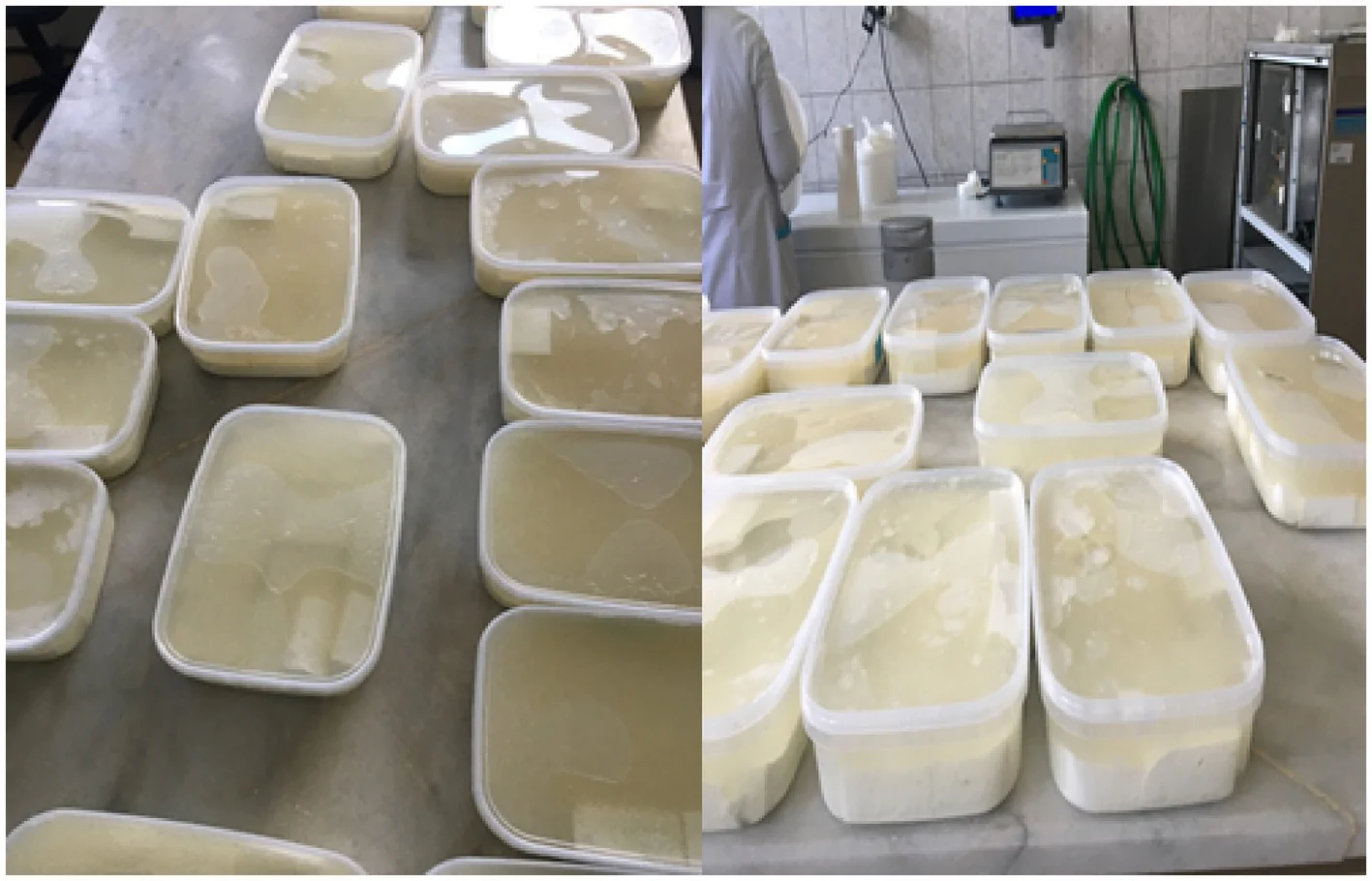
White cheese with stabilizer added to its brine.

#### 2.2.3 Preparation of gel and brines

##### 2.2.3.1 Pectin-containing brine

In stainless steel tanks, mixtures containing 1.0, 2.0, and 3.0% pectin, and separately 2.0, 4.0, and 6.0% salt were weighed and dissolved in tap water to prepare brines. The thickened brines were pasteurized at 85 °C for 15 min, then allowed to cool (30–35 °C) and subsequently placed into 2 kg plastic containers for the test cheeses.

##### 2.2.3.2 Xanthan gum-containing brine

Brines were prepared by weighing mixtures containing 0.5, 1.0, and 1.5% xanthan gum and 2.0, 4.0, and 6% salt separately in stainless steel vats and dissolving them in tap water. The thickened brines were pasteurized at 85 °C for 15 min, then allowed to cool (30–35 °C) and subsequently placed into 2 kg plastic containers for the test cheeses.

##### 2.2.3.3 Carrageenan gum brine

In stainless steel vats, mixtures containing 0.5, 1.0, and 1.5% carrageenan gum, and separately 2.0, 4.0, and 6.0% salt were weighed and dissolved in tap water to prepare brines. The thickened brines were pasteurized at 85 °C for 15 min, then allowed to cool (30–35 °C) before being placed in plastic containers, 2 kg each, containing the test cheeses.

##### 2.2.3.4 Gelatin-containing brine

Brines were prepared by weighing mixtures containing 1.0, 2.0, and 3.0% beef gelatin and 2.0, 4.0, and 6.0% salt separately in stainless steel vats and dissolving them in tap water. The gel brines were pasteurized at 85 °C for 15 min, then allowed to cool (30–35 °C) and subsequently placed into 2 kg plastic containers for the test cheeses.

##### 2.2.3.5 Control brine

In stainless steel tanks, refined sea salt at concentrations of 2.0, 4.0, and 6.0% was weighed and dissolved in tap water to prepare brines. These brines were pasteurized at 85 °C for 15 min, then allowed to cool (30–35 °C) before being placed in 2 kg portions of each plastic container for the sample cheeses.

#### 2.2.4 Analyses of the materials used and products produced

##### 2.2.4.1 Milk analyses

The pH of raw milk used in white cheese production was determined by titration using a Hanna-Instrument pH microprocessor benchtop pH meter, the acidity was determined by the titration method in terms of Soxhlet-Henkel (^o^SH), the dry matter content was determined by the gravimetric method, and the fat content was determined by the Gerber method (17). The total nitrogen content of the milk was determined by the Kjeldahl method, and this value was multiplied by a factor of 6.38 to find the % protein content. The casein content was accepted as 80% of the protein content. The presence of soda in the milk was determined by the rosalic acid test, and the presence of antibiotics (AuroFlow™ Prime™ Beta Lactam Mrl) and peroxides (hydrogen peroxide kit, CB5150) was determined with ready-made kits (17).

##### 2.2.4.2 Determination of the amount of cheese rennet

The amount of rennet to be added to the milk is a function of the coagulation time. While factors such as the acidity, composition, temperature of the milk, and the strength of the rennet affect the amount of rennet, the most important factor is the coagulation time. As the coagulation time we determine increases, the amount of rennet to be added also increases. In white cheese, the coagulation time is generally 60–120 min (17).

In the renneting stage of cheese at 42 °C, 25 mL of milk was taken and placed in an Erlenmeyer flask. Then, 1 mL of the rennet solution, prepared by diluting it with water at a ratio of 1/10, was added to the milk in the Erlenmeyer flask. The milk and rennet mixture in the Erlenmeyer flask was slowly stirred, and a stopwatch was started. The stopwatch was stopped at the moment the first curd formation was observed, and the elapsed time was recorded in seconds. As a result, the amount of rennet to be added to the milk was determined by calculations (18).

Yeast amount(ml)=AxBxC/DxE.

A: Amount of yeast used in the experiment (ml).

B: Amount of milk in the vat (L).

C: Coagulation time of the milk used in the experiment (s).

D: Amount of milk used in the experiment (L).

E: Desired curd formation time in the milk in the vat (s).

##### 2.2.4.3 Physicochemical analyses

The yield of the cheese samples was calculated using the following formula:

Yield(%)=Amount ofRawCheese(kg)/Amount of Milk(L)x100.

The pH values of the cheese samples were measured using a benchtop pH meter. 10 g of each sample was weighed, 40 mL of distilled water was added, and the mixture was homogenized with an Ultra-Turrax homogenizer for 2 min. The pH meter electrode was then immersed in this homogeneous liquid and the readings were recorded (17). For the total acidity analysis, 10 g of sample was weighed, 40 mL of distilled water was added, and the mixture was homogenized for 2 min; then, after adding a few drops of phenolphthalein as an indicator, titration was performed with 0.1 N NaOH. Total acidity was calculated as lactic acid based on the consumption (17). Dry matter content was determined by the gravimetric method (19). Fat content was determined as % fat using the Van Gulik method (17). After the dry matter analysis, cheese samples were analyzed using a Gerber centrifuge (Funke Gerber FG3670, Germany) to determine their fat content; and fat ratio was calculated on a dry matter basis. The salt content of cheese samples was determined by titration with 0.1 N silver nitrate using the Mohr method and expressed as % salt. For this purpose, 5 g of sample was taken and ground in a mortar; the sample was then diluted to 250 mL with hot water (65 °C) to allow salt transfer into water. After filtering through coarse filter paper, 10 mL of the filtrate was taken and titrated with 0.1 N silver nitrate after adding potassium chromate as an indicator (17). Protein content was calculated by determining the nitrogen content of samples subjected to wet digestion using the Micro Kjeldahl method and multiplying the resulting value by the conversion factor of 6.38 applicable to dairy products, expressed as % (20). The ash content of cheese samples was determined by combustion in an ash furnace at 500–600 °C for 5–6 h (M1811, Elektromag, Istanbul, Turkey) (17). Water activity (a_w_) values of cheese samples were determined using an automatic water activity meter (Aqua Lab 2.0, USA) (21).

##### 2.2.4.4 Color analyses

A reflectance Tintometer (Lovibond Reflectance Tintometer 962, Canada) was used to determine the color properties of the cheeses. L* values (ranging from black to white) measured the lightness–darkness, a* values measured green–red, and b* values measured yellow–blue of the samples (22).

##### 2.2.4.5 Microbiological analyses

For microbiological analyses of cheeses, cultural counting methods were employed for lactic acid bacteria, yeast-mold, and total mesophilic aerobic bacteria counts. To test the microbiological properties of the samples, 25 g of sample and 225 mL of sterile Ringer solution were used to prepare serial dilutions (23) to enumerate total lactic acid bacteria (*Streptococci* and *Lactococci*), total mesophilic aerobic bacteria, and total yeast-mold counts.

*Lactobacilli* in cheese were enumerated using MRS Agar after 2–3 days of incubation at 30 °C and expressed as colony-forming units per gram (cfu/g) of cheese (24). *Lactococci* were enumerated using M17 agar after 3–4 days of incubation at 30 °C (25); results were also expressed as cfu/g of cheese. Total bacterial counts (TMAB) were determined by counting colonies that developed after 24 h of incubation at 30 °C on Plate Count Agar using the surface spread method (24). Total yeast-mold counts were determined by counting colonies that developed after 3–5 days of incubation at 25 °C on Dichloran Rose Bengal Chloramphenicol Agar using the surface spread method (24).

##### 2.2.4.6 Statistical analyses

Data obtained regarding the physicochemical, color, and microbiological properties of the white cheeses stored with different salt and stabilizer concentrations were evaluated using analysis of variance (ANOVA) with the JMP PRO statistical software package. Statistical analyses were performed for control samples and cheeses containing stabilizers + salt in their brines. Significant differences between means were identified using the Tukey multiple comparison test at a significance level of *p* < 0.05 (26). Principal component analysis (PCA), hierarchical clustering analysis (HCA), and heat map analysis were performed using XLSTAT software (XLSTAT, 2008, Addinsoft, New York, NY, USA) to categorize cheese samples according to their physicochemical, microbiological, and color properties. Additionally, Pearson correlations between all properties were determined with XLSTAT software.

## 3 Results

### 3.1 Characteristics of milk used in white cheese production

Cow’s milk was used as a raw material in the production of cheeses. The composition of cow’s milk varies depending on the animal breed, age, and type of feeding. The amount of milk used (yield) to produce 1 kg of brine-cured white cheese is 6 liters. In this study, the total dry matter of the milk used in the production of cheeses ranged from 11.90 to 12.05%. The fat content of the milk we used in our study ranged from 3.62 to 3.68%, the protein content from 3.30 to 3.39%, the pH values from 6.61 to 6.64, and the SH values from 6.6 to 6.7. No additives or residues such as hydrogen peroxide, neutralizing agents, or antibiotics were detected in the milk. Since the milk was obtained from the university’s cow farm where the study was conducted, it maintained its freshness until the production stage and was found to be safe in terms of milk adulteration.

### 3.2 Physicochemical properties

Dry matter analysis was performed on white cheese samples produced by adding different stabilizers to their brines, and the results are presented in Table 1. Examination of this table reveals that the day 1 dry matter values of the samples are very close to each other. Over the 90-day storage period, dry matter values began to differ. The values exhibited fluctuations throughout the storage period. On the first day of storage, the lowest dry matter value was found in the cheese with carrageenan gum in its brine, while the highest dry matter value was measured in the control sample. According to the Turkish Standards Institute for white cheese, cheese must contain at least 40% dry matter. While the initial values of the produced cheeses met the standards, the dry matter ratio decreased during storage as the cheeses absorbed some water, ultimately falling below the standard threshold (Table 1).

**Table 1:** Dry matter values measured during storage in white cheese products containing different stabilizers and salts in their brine (%).

Stabilizer	Stabilizer % - Salt %	1st day	15th day	30th day	60th day	90th day
Carrageenan gum	0–2	43.112 ± 0.21^Aba^	40.484 ± 0.30^Ab^	36.077 ± 0.25^Bc^	-	-
	0–4	42.262 ± 0.42^ABCa^	39.545 ± 0.44^Ab^	-	-	-
	0–6	43.463 ± 0.42^Aa^	40.506 ± 0.51^Ab^	37.126 ± 0.20^ABc^	-	-
	0.5–2	40.461 ± 0.71^ABCa^	36.234 ± 0.39^CDEb^	33.731 ± 0.67^Cc^	33.373 ± 0.50^Cc^	-
	0.5–4	38.442 ± 1.39^BCDa^	34.413 ± 0.22^Eb^	32.282 ± 0.46^DEb^	-	-
	0.5–6	38.214 ± 1.27^CDa^	34.472 ± 0.16^Eb^	32.000 ± 0.28^DEb^	-	-
	1–2	43.450 ± 0.44^Aa^	39.800 ± 0.36^Ab^	38.285 ± 0.18^Ab^	38.165 ± 0.24^Ab^	37.523 ± 0.17^Ab^
	1–4	42.434 ± 0.12^ABCa^	38.635 ± 0.30^ABb^	34.576 ± 0.17^Cc^	33.052 ± 0.25^Cc^	32.278 ± 0.22^Ac^
	1–6	35.345 ± 2.00^Da^	36.544 ± 0.40^CDa^	33.463 ± 0.25^Ca^	-	-
	1.5–2	42.300 ± 0.86^ABCa^	37.431 ± 0.44^BCb^	34.432 ± 0.37^Cbc^	32.325 ± 0.23^Cc^	32.053 ± 0.21^Ac^
	1.5–4	42.017 ± 2.17^ABCa^	38.890 ± 0.51^ABab^	37.294 ± 0.13^ABab^	35.844 ± 0.47^Bb^	34.963 ± 0.35^Ab^
	1.5–6	38.622 ± 2.04^ABCDa^	35.010 ± 1.10^DEab^	32.425 ± 0.20^DEb^	-	-
Xanthan gum	0–2	43.112 ± 0.21^Aba^	40.484 ± 0.30^Ab^	36.077 ± 0.25^ABc^	-	-
	0–4	42.262 ± 0.42^Ba^	39.545 ± 0.44^ABb^	-	-	-
	0–6	43.463 ± 0.42^Aa^	40.506 ± 0.51^Aab^	37.126 ± 0.20^Ab^	-	-
	0.5–2	38.360 ± 0.00^FGa^	35.734 ± 0.25^CDEab^	33.044 ± 1.11^CDb^	-	-
	0.5–4	40.252 ± 0.16^CDEa^	35.196 ± 0.30^CDEb^	28.265 ± 1.00^Gc^	-	-
	0.5–6	39.394 ± 0.16^EFa^	35.685 ± 0.65^CDEb^	31.650 ± 0.55^CDEFc^	-	-
	1–2	40.493 ± 0.18^CDa^	37.125 ± 0.18^BCDb^	32.534 ± 0.64^CDc^	-	-
	1–4	40.706 ± 0.15^Ca^	37.894 ± 1.75^ABCab^	34.010 ± 0.51^BCb^	-	-
	1–6	39.558 ± 0.44^DEa^	36.867 ± 1.36^BCDa^	29.078 ± 0.17^FGb^	-	-
	1.5–2	37.965 ± 0.14^Ga^	35.263 ± 0.42^CDEb^	31.963 ± 0.33^CDEc^	-	-
	1.5–4	37.450 ± 0.31^GHa^	34.400 ± 0.62^DEb^	29.696 ± 0.15^EFGc^	-	-
	1.5–6	36.562 ± 0.19^Ha^	33.084 ± 1.16^Eb^	31.076 ± 0.22^DEFb^	-	-
Gelatin	0–2	43.112 ± 0.21^Aa^	40.484 ± 0.30^Ab^	36.077 ± 0.25^ABc^	-	-
	0–4	42.262 ± 0.42^ABa^	39.545 ± 0.44^ABb^	-	-	-
	0–6	43.463 ± 0.42^Aa^	40.506 ± 0.51^Aab^	37.126 ± 0.20^Ab^	-	-
	1–2	37.971 ± 0.41^Ca^	36.175 ± 0.27^Eb^	33.722 ± 0.75^CDc^	31.110 ± 0.21^BCd^	29.955 ± 0.21^Ad^
	1–4	39.353 ± 0.31^BCa^	35.843 ± 0.26^Eb^	33.035 ± 0.18^CDc^	-	-
	1–6	40.542 ± 0.78^ABCa^	37.450 ± 0.28^CDb^	34.523 ± 0.18^BCc^	-	-
	2–2	42.325 ± 1.47^ABa^	39.324 ± 0.26^ABb^	36.464 ± 0.30^ABc^	31.960 ± 0.14^ABd^	29.913 ± 0.14^Ad^
	2–4	41.344 ± 0.13^ABa^	38.606 ± 0.13^BCb^	35.020 ± 0.16^ABCc^	31.114 ± 0.26^Cd^	28.329 ± 0.20^Ae^
	2–6	37.950 ± 0.13^Ca^	34.454 ± 0.23^Fb^	32.116 ± 0.22^DEc^	-	-
	3–2	43.544 ± 0.41^Aa^	39.983 ± 0.17^Ab^	37.075 ± 0.25^Ac^	32.756 ± 0.26^Ad^	29.142 ± 0.26^Ae^
	3–4	39.946 ± 1.42^BCa^	36.512 ± 0.35^DEb^	33.561 ± 0.43^CDb^	30.337 ± 0.23^Cc^	-
	3–6	37.647 ± 1.39^Ca^	32.773 ± 0.38^Gb^	30.513 ± 0.31^Ebc^	28.113 ± 0.74^Dc^	
Pectin	0–2	43.112 ± 0.21^ABa^	40.484 ± 0.30^Ab^	36.077 ± 0.25^ABc^	-	-
	0–4	42.262 ± 0.42^ABCDa^	39.545 ± 0.44^ABb^	-	-	-
	0–6	43.463 ± 0.42^ABa^	40.506 ± 0.51^Aab^	37.126 ± 0.20^ABb^	-	-
	1–2	39.972 ± 0.15^CDa^	37.396 ± 0.37^BCb^	-	-	-
	1–4	44.813 ± 0.10^Aa^	40.960 ± 0.14^Ab^	37.485 ± 0.30^Ac^	34.112 ± 0.26^Ad^	-
	1–6	40.334 ± 0.54^BCDa^	37.606 ± 0.52^BCb^	33.435 ± 0.23^CDc^	30.363 ± 0.32^Cd^	
	2–2	42.900 ± 0.18^ABCa^	37.545 ± 1.24^BCb^	35.113 ± 0.21^BCb^	31.814 ± 0.31^Bc^	-
	2–4	42.760 ± 1.29^ABCDa^	36.487 ± 0.26^CDb^	-	-	-
	2–6	43.145 ± 0.15^ABa^	36.723 ± 0.77^CDb^	-	-	
	3–2	40.986 ± 1.27^BCDa^	34.832 ± 0.02^Db^	31.457 ± 0.26^Dc^	-	-
	3–4	42.674 ± 0.04^ABCDa^	35.534 ± 0.44^CDb^	31.960 ± 0.13^Dc^	-	-
	3–6	39.714 ± 1.86^Aa^	37.237 ± 0.73^Da^	-	-	-

A–G: Uppercase letters within the same column indicate comparisons among cheese varieties across storage periods.

a–d: Lowercase letters within the same row indicate comparisons among samples at different storage periods. Different letters indicate statistically significant differences between samples, whereas identical letters indicate no statistically significant difference.

**-**: Because of the cheese samples had melted, analysis did not be performed.

The lowest dry matter value was measured at 28.265% in the cheese with 0.5% xanthan gum and 4% salt in its brine on day 30. The highest dry matter value was measured as 44.813% on day 1 in the cheese with 1% pectin and 4% salt in its brine. While the sample with the highest dry matter content at the beginning of storage was the cheese containing gelatin in its brine, the sample with the highest value at the end of storage was the cheese containing carrageenan gum in its brine (Table 1). The decrease in dry matter values during storage of white cheese samples containing different stabilizers and salt concentrations in their brines may be attributed to water migration from the brine into the cheese matrix, resulting in increased moisture content and consequently a reduction in dry matter percentage. The differences among treatments could be related to the water-binding capacity of the stabilizers and their effects on the cheese microstructure.

Fat analysis was performed on white cheese samples with different stabilizers added to their brines using the Gerber method, and the results are presented in Table 2. The lowest fat value was measured as 14.14% on day 60 in the cheese with 1% pectin stabilizer and 4% salt in its brine. The highest fat value was measured as 18.90% on day 1 in the cheese with 0.5% carrageenan gum and 6% salt in its brine. All cheeses produced in this study were made from full-fat milk. Therefore, as can be seen in Table 2, the fat ratios of the samples were relatively high on the first day. A substantial decrease in the fat ratio within the cheese mass was observed on day 90 of storage. The decrease in fat percentage during storage was mainly attributed to water migration into the cheese matrix, which increased moisture content and caused a proportional reduction in fat percentage when expressed on a wet basis. Although some fat migration from the cheese into the brine may have occurred, this loss was considered limited and unlikely to be the primary cause of the observed decrease. The fat content of the produced cheeses could have been higher; however, high-temperature pasteurization was applied to simulate industrial cheese production conditions, which may have influenced the cheese matrix characteristics and water retention capacity. In fact, the increase in ash values in our cheeses was almost entirely related to salt content.

**Table 2:** Fat values measured during storage in white cheese products containing different stabilizers and salts in their brine (%).

Stabilizer	Stabilizer % - Salt %	1st day	15th day	30th day	60th day	90th day
Carrageenan gum	0–2	18.25 ± 0.35^ABa^	14.38 ± 0.53^Cb^	14.16 ± 0.06^Fb^	-	-
	0–4	18.45 ± 0.64^ABa^	14.75 ± 0.35^Cb^	-	-	-
	0–6	18.48 ± 0.74^ABa^	14.20 ± 0.14^Cb^	14.00 ± 0.03^Fb^	-	-
	0.5–2	18.75 ± 0.35^Aa^	18.25 ± 0.35^Aa^	15.50 ± 0.35^Cb^	15.10 ± 0.14^Ab^	-
	0.5–4	17.70 ± 0.14^ABa^	17.25 ± 0.35^ABab^	16.35 ± 0.26^Bb^	-	-
	0.5–6	18.90 ± 0.14^Aa^	18.25 ± 0.35^Aa^	17.30 ± 0.24^Ab^	-	-
	1–2	18.27 ± 0.45^ABa^	17.75 ± 0.35^ABab^	15.13 ± 0.18^CDEc^	15.50 ± 0.71^Ac^	14.25 ± 0.35^Bbc^
	1–4	17.35 ± 0.78^ABa^	17.13 ± 0.18^ABa^	14.65 ± 0.21^DEFb^	15.25 ± 0.35^Ab^	15.13 ± 0.18^ABa^
	1–6	17.75 ± 0.35^ABa^	17.00 ± 0.35^ABab^	16.40 ± 0.19^Bb^	-	-
	1.5–2	17.13 ± 0.18^ABa^	16.80 ± 0.28^Bab^	15.13 ± 0.18^CDEc^	16.25 ± 0.35^Ab^	16.05 ± 0.21^ABa^
	1.5–4	17.20 ± 0.42^ABa^	16.80 ± 0.42^Bab^	15.35 ± 0.35^CDb^	16.30 ± 0.28^Aab^	15.75 ± 0.35^Aa^
	1.5–6	16.75 ± 0.35^ABa^	16.50 ± 0.42^Ba^	14.38 ± 0.18^EFb^	-	-
Xanthan gum	0–2	18.25 ± 0.35^Aa^	14.38 ± 0.53^CDb^	14.16 ± 0.06^ABb^	-	-
	0–4	18.45 ± 0.64^Aa^	14.75 ± 0.35^CDb^	-	-	-
	0–6	18.48 ± 0.74^Aa^	14.20 ± 0.14^Db^	14.00 ± 0.03^Bb^	-	-
	0.5–2	17.25 ± 0.35^ABa^	16.50 ± 0.71^ABa^	15.03 ± 0.67^ABa^	-	-
	0.5–4	17.50 ± 0.71^ABa^	15.25 ± 0.35^ABCDa^	15.50 ± 0.71^ABa^	-	-
	0.5–6	16.75 ± 0.35^ABa^	15.65 ± 0.64^ABCDa^	15.88 ± 0.88^ABa^	-	-
	1–2	17.75 ± 0.35^Aa^	15.05 ± 0.07^BCDb^	15.80 ± 0.28^ABb^	-	-
	1–4	15.00 ± 1.41^Ba^	15.75 ± 0.35^ABCDa^	15.65 ± 0.78^ABa^	-	-
	1–6	17.25 ± 1.06^ABa^	15.88 ± 0.18^ABCa^	15.35 ± 0.21^ABa^	-	-
	1.5–2	16.38 ± 0.18^ABa^	16.75 ± 0.49^Aa^	15.44 ± 0.16^ABa^	-	-
	1.5–4	16.75 ± 0.35^ABa^	16.00 ± 0.42^ABCa^	15.80 ± 0.28^ABa^	-	-
	1.5–6	16.65 ± 0.64^ABa^	16.63 ± 0.18^ABa^	16.00 ± 0.14^Aa^	-	-
Gelatin	0–2	18.25 ± 0.35^Aa^	14.38 ± 0.53^Ab^	14.16 ± 0.06^Bb^	-	-
	0–4	18.45 ± 0.64^Aa^	14.75 ± 0.35^Ab^	-	-	-
	0–6	18.48 ± 0.74^Aa^	14.20 ± 0.14^Ab^	14.00 ± 0.03^Bb^	-	-
	1–2	18.50 ± 0.71^Aa^	15.50 ± 0.71^Ab^	15.13 ± 0.53^ABb^	15.14 ± 00^Ab^	15.00 ± 0.14^Ab^
	1–4	18.25 ± 0.35^Aa^	15.25 ± 0.35^Ab^	14.50 ± 0.42^ABb^	-	-
	1–6	17.50 ± 0.71^Aa^	15.13 ± 0.88^Aa^	14.75 ± 0.35^ABa^	-	-
	2–2	18.75 ± 0.35^Aa^	15.63 ± 0.18^Ab^	15.25 ± 0.35^ABb^	15.18 ± 00^Ab^	15.75 ± 0.35^Ab^
	2–4	18.13 ± 0.53^Aa^	16.15 ± 0.92^Aab^	15.38 ± 0.18^ABab^	15.24 ± 00^Ab^	15.18 ± 0.32^Aab^
	2–6	17.75 ± 1.41^Aa^	15.00 ± 1.41^Aa^	14.75 ± 0.35^ABa^	-	-
	3–2	17.75 ± 0.35^Aa^	16.13 ± 0.53^Ab^	15.75 ± 0.35^Ab^	15.18 ± 00^Ab^	15.00 ± 0.14^Ab^
	3–4	18.00 ± 1.41^Aa^	15.75 ± 0.35^Aa^	15.00 ± 0.71^ABa^	15.35 ± 00^Aa^	-
	3–6	17.88 ± 0.53^Aa^	15.63 ± 0.18^Ab^	14.88 ± 0.18^ABb^	14.55 ± 0.1^Ab^	-
Pectin	0–2	18.25 ± 0.35^Aa^	14.38 ± 0.53^Ab^	14.16 ± 0.06^ABb^	-	-
	0–4	18.45 ± 0.64^Aa^	14.75 ± 0.35^Ab^	-	-	-
	0–6	18.48 ± 0.74^Aa^	14.20 ± 0.14^Ab^	14.00 ± 0.03^Bb^	-	-
	1–2	18.25 ± 0.35^Aa^	15.25 ± 0.35^Ab^	-	-	-
	1–4	17.75 ± 0.21^Aa^	15.15 ± 0.07^Ab^	14.90 ± 0.14^ABbc^	14.14 ± 0^Ac^	-
	1–6	16.90 ± 0.14^Aa^	15.25 ± 0.35^Ab^	15.05 ± 0.07^Ab^	14.28 ± 0^Ab^	-
	2–2	17.00 ± 0.71^Aa^	14.25 ± 0.35^Ab^	14.90 ± 0.57^ABb^	14.14 ± 0^Ab^	-
	2–4	18.40 ± 0.85^Aa^	14.75 ± 0.35^Ab^	-	-	-
	2–6	18.00 ± 0.57^Aa^	14.90 ± 0.14^Ab^	-	-	-
	3–2	18.85 ± 0.35^Aa^	14.95 ± 0.21^Ab^	14.65 ± 0.21^ABb^	-	-
	3–4	17.90 ± 0.85^Aa^	14.80 ± 0.42^Ab^	14.20 ± 0.18^ABb^	-	-
	3–6	18.80 ± 0.28^Aa^	15.06 ± 0.34^Ab^	-	-	-

A–G: Uppercase letters within the same column indicate comparisons among cheese varieties across storage periods.

a–d: Lowercase letters within the same row indicate comparisons among samples at different storage periods. Different letters indicate statistically significant differences between samples, whereas identical letters indicate no statistically significant difference.

**-**: Because of the cheese samples had melted, analysis did not be performed.

Ash values began to differ during storage. The values exhibited a fluctuating course throughout the storage period. At the end of maturation, ash values generally increased compared to the beginning of storage. Ash analysis was performed on white cheese samples with different stabilizers added to their brine, and results are presented in Table 3. The lowest ash value was measured as 1.66% on day 1 in the cheese with 2% gelatin and 2% salt in its brine. The highest ash value was measured as 3.67% on day 30 in the cheese with 0.5% xanthan and 6% salt in its brine. While the highest ash content at the beginning of storage was in the control group cheeses, the highest value at the end of storage was measured in the cheese containing gelatin in its brine. The ash values in white cheese samples with different stabilizers and salt levels in their brines were observed to increase throughout storage.

**Table 3:** Ash values measured during storage in white cheese products containing different stabilizers and salts in their brine (%).

Stabilizer	Stabilizer % - Salt %	1st day	15th day	30th day	60th day	90th day
Carrageenan gum	0–2	2.35 ± 0.07^CDa^	2.45 ± 0.07^EFa^	2.55 ± 0.07^CDa^	-	-
	0–4	2.61 ± 0.08^Ba^	2.73 ± 0.11^Ca^	-	-	-
	0–6	3.04 ± 0.08^Aab^	3.25 ± 0.07^Ba^	2.96 ± 0.04^Ab^	-	-
	0.5–2	2.51 ± 0.08^BCa^	2.74 ± 0.06^Ca^	2.62 ± 0.05^BCa^	2.69 ± 0.05^Aa^	-
	0.5–4	2.32 ± 0.06^CDEb^	2.62 ± 0.04^CDa^	2.65 ± 0.04^BCa^	-	-
	0.5–6	3.19 ± 0.02^Ab^	3.43 ± 0.07^Aa^	2.76 ± 0.01^ABc^	-	-
	1–2	1.94 ± 0.01^Fc^	2.16 ± 0.06^Gab^	2.09 ± 0.01^Fb^	2.28 ± 0.04^Ba^	2.20 ± 0.05^Bab^
	1–4	2.18 ± 0.09^DEb^	2.47 ± 0.06^DEa^	2.18 ± 0.08^EFb^	2.42 ± 0.04^Bab^	2.44 ± 0.04^Aab^
	1–6	3.12 ± 0.02^Ab^	3.38 ± 0.04^ABa^	2.35 ± 0.9^DEc^	-	-
	1.5–2	1.92 ± 0.02^Fb^	2.25 ± 0.09^Ga^	2.16 ± 0.06^EFab^	2.26 ± 0.08^Ba^	2.30 ± 0.03^ABa^
	1.5–4	2.10 ± 0.03^EFb^	2.31 ± 0.08^FGab^	2.19 ± 0.06^EFab^	2.31 ± 0.05^Bab^	2.33 ± 0.05^ABa^
	1.5–6	2.22 ± 0.02^DEb^	2.46 ± 0.06^Ea^	2.30 ± 0.06^Eab^	-	-
Xanthan gum	0–2	2.35 ± 0.07^DEa^	2.45 ± 0.07^ABa^	2.55 ± 0.07^DEa^	-	-
	0–4	2.61 ± 0.08^CDa^	2.73 ± 0.11^ABa^	-	-	-
	0–6	3.04 ± 0.08^ABab^	3.25 ± 0.07Aa	2.96 ± 0.04^Bb^	-	-
	0.5–2	1.93 ± 0.05^Fa^	2.17 ± 0.00^Ba^	2.22 ± 0.05^Fa^	-	-
	0.5–4	2.55 ± 0.12^CDa^	2.56 ± 0.06^ABa^	2.76 ± 0.10^BCDa^	-	-
	0.5–6	3.17 ± 0.07^Aa^	2.86 ± 0.06^ABa^	3.67 ± 0.02^Aa^	-	-
	1–2	1.97 ± 0.02^Fb^	2.18 ± 0.08^Bab^	2.35 ± 0.06^EFa^	-	-
	1–4	2.25 ± 0.12^Eb^	2.37 ± 0.08^ABb^	2.75 ± 0.03^BCDa^	-	-
	1–6	2.76 ± 0.04^BCb^	2.97 ± 0.06^ABb^	3.58 ± 0.06^Aa^	-	-
	1.5–2	2.10 ± 0.04^EFa^	2.20 ± 0.06^Ba^	2.31 ± 0.08^EFa^	-	-
	1.5–4	2.33 ± 0.01^DEb^	2.43 ± 0.04^ABb^	2.90 ± 0.13^BCa^	-	-
	1.5–6	2.36 ± 0.02^DEb^	2.47 ± 0.05^ABab^	2.66 ± 0.08^CDa^	-	-
Gelatin	0–2	2.35 ± 0.07^Ba^	2.45 ± 0.07^Ba^	2.55 ± 0.07^Da^	-	-
	0–4	2.61 ± 0.08^Ba^	2.73 ± 0.11^Ba^	-	-	-
	0–6	3.04 ± 0.08^Aab^	3.25 ± 0.07^Aa^	2.96 ± 0.04^Cb^	-	-
	1–2	1.71 ± 0.08^Cc^	1.88 ± 0.01^CDbc^	2.18 ± 0.11^Fa^	2.03 ± 0.04^Dab^	2.18 ± 0.04^BCa^
	1–4	2.35 ± 0.07^Ba^	2.47 ± 0.06^Ba^	2.56 ± 0.06^Da^	-	-
	1–6	2.92 ± 0.08^Aa^	3.09 ± 0.06^Aa^	3.18 ± 0.04^Ba^	-	-
	2–2	1.66 ± 0.08^Cc^	1.74 ± 0.19^Dbc^	1.99 ± 0.06^Gabc^	2.10 ± 0.06^Dab^	2.17 ± 0.04^Ca^
	2–4	1.89 ± 0.04^Cc^	2.10 ± 0.10^Cbc^	2.36 ± 0.06^Eab^	2.35 ± 0.07^Cab^	2.41 ± 0.08^Ba^
	2–6	3.03 ± 0.10^Ab^	3.33 ± 0.04^Aab^	3.45 ± 0.07^Aa^	-	-
	3–2	1.91 ± 0.06^Cc^	2.08 ± 0.02^Cbc^	2.28 ± 0.04^EFb^	2.48 ± 0.04^Ca^	2.65 ± 0.06^Aa^
	3–4	2.32 ± 0.05^Bb^	2.53 ± 0.03^Bab^	2.66 ± 0.06^Da^	2.75 ± 0.07^Ba^	-
	3–6	2.95 ± 0.07^Ac^	3.19 ± 0.04^Aab^	3.36 ± 0.06^Aa^	2.99 ± 0.05^Abc^	-
Pectin	0–2	2.35 ± 0.07^CDEa^	2.45 ± 0.07^Da^	2.55 ± 0.07^Ba^	-	-
	0–4	2.61 ± 0.08^BCa^	2.73 ± 0.11^CDa^	-	-	-
	0–6	3.04 ± 0.08^Aab^	3.25 ± 0.07^ABa^	2.96 ± 0.04^Ab^	-	-
	1–2	2.05 ± 0.19^EFGa^	1.99 ± 0.08^Ea^	-	-	-
	1–4	1.82 ± 0.05^Gb^	1.93 ± 0.04^Eb^	2.15 ± 0.07^Ca^	2.28 ± 0.04^Aa^	-
	1–6	1.90 ± 0.06^FGb^	2.07 ± 0.07^Eab^	2.22 ± 0.10^Ca^	2.23 ± 0.05^Aa^	-
	2–2	1.83 ± 0.12^FGab^	2.01 ± 0.05^Ea^	2.26 ± 0.08^Ca^	2.23 ± 0.04^Ab^	-
	2–4	2.44 ± 0.05^BCDa^	2.57 ± 0.05^Da^	-	-	-
	2–6	3.06 ± 0.06^Aa^	3.30 ± 0.10^Aa^	-	-	-
	3–2	1.73 ± 0.11^Gb^	1.84 ± 0.15^Eab^	2.19 ± 0.05^Ca^	-	-
	3–4	2.18 ± 0.04^DEFb^	2.41 ± 0.04^Da^	2.37 ± 0.04^BCa^	-	-
	3–6	2.75 ± 0.07^ABa^	2.96 ± 0.08^BCa^	-	-	-

A–G: Uppercase letters within the same column indicate comparisons among cheese varieties across storage periods.

a–d: Lowercase letters within the same row indicate comparisons among samples at different storage periods. Different letters indicate statistically significant differences between samples, whereas identical letters indicate no statistically significant difference.

**-**: Because of the cheese samples had melted, analysis did not be performed.

Protein analysis was performed on white cheese samples with different stabilizers added to their brines, and the results are presented in Table 4. A decrease in protein content was observed in all samples at the end of the storage period. The lowest protein content was measured as 11.04% on day 30 in the cheese with 1% xanthan and 4% salt in its brine. The highest protein content was measured as 14.49% on day 1 in the cheese with 1% xanthan gum and 4% salt in its brine. At the beginning of storage, the highest protein content was in the cheese containing gelatin in its brine, while at the end of storage the highest value was measured in the cheese containing carrageenan gum in its brine. A decrease in protein values during storage was observed in white cheese samples with different stabilizers and salt levels in their brines.

**Table 4:** Protein values measured during storage in white cheese products containing different stabilizers and salts in their brine (%).

Stabilizer	Stabilizer % - Salt %	1st day	15th day	30th day	60th day	90th day
Carrageenan gum	0–2	16.96 ± 0.11^Aa^	15.54 ± 0.11^Ab^	12.6 ± 0.23^CDEc^	-	-
	0–4	16.50 ± 0.05^Aa^	13.77 ± 0.23^Cb^	-	-	-
	0–6	16.88 ± 0.11^Aa^	13.52 ± 0.11^CDb^	12.86 ± 0.20^BCDc^	-	-
	0.5–2	15.54 ± 0.35^Ba^	13.35 ± 0.11^Db^	12.51 ± 0.11^DEc^	11.67 ± 0.11^Bd^	-
	0.5–4	14.91 ± 0.17^Ba^	13.35 ± 0.11^Db^	11.84 ± 0.11^Ec^	-	-
	0.5–6	17.13 ± 0.23^Aa^	14.7 ± 0.11^Bb^	13.60 ± 0.23^ABc^	-	-
	1–2	16.59 ± 0.29^Aa^	15.58 ± 0.17^Ab^	13.44 ± 0.23^ABCc^	13.86 ± 0.11^Ac^	12.34 ± 0.11^Bd^
	1–4	17.17 ± 0.17^Aa^	14.74 ± 0.17^Bb^	13.77 ± 0.23^Abc^	13.25 ± 0.20^Ac^	12.95 ± 0.12^ABc^
	1–6	17.05 ± 0.35^Aa^	14.99 ± 0.17^Bb^	13.90 ± 0.17^Ac^	-	-
	1.5–2	16.8 ± 0.23^Aa^	15.52 ± 0.14^Ab^	13.23 ± 0.29^ABCDc^	11.59 ± 0.23^Bd^	13.02 ± 0.11^ABc^
	1.5–4	16.59 ± 0.17^Aa^	14.95 ± 0.23^Bb^	13.35 ± 0.35^ABCDc^	11.42 ± 0.23^Bd^	13.81 ± 0.17^Ac^
	1.5–6	16.96 ± 0.23^Aa^	14.95 ± 0.23^Bb^	13.27 ± 0.23^ABCDc^	-	-
Xanthan gum	0–2	16.96 ± 0.11^Aa^	15.54 ± 0.11^ABb^	12.6 ± 0.23^Dc^	-	-
	0–4	16.50 ± 0.05^Aa^	13.77 ± 0.23^EFb^	-	-	-
	0–6	16.88 ± 0.11^Aa^	13.52 ± 0.11^Fb^	12.86 ± 0.20^Dc^	-	-
	0.5–2	17.22 ± 0.11^Aa^	16.12 ± 0.23^Ab^	15.62 ± 0.23^Ab^	-	-
	0.5–4	16.67 ± 0.17^Aa^	15.87 ± 0.11^Ab^	14.36 ± 0.11^Cc^	-	-
	0.5–6	15.62 ± 0.23^Ba^	14.15 ± 0.17^DEFb^	13.35 ± 0.11^Dc^	-	-
	1–2	17.30 ± 0.23^Aa^	15.07 ± 0.29^BCb^	15.20 ± 0.11^ABb^	-	-
	1–4	14.49 ± 0.29^Da^	12.01 ± 0.11^Gb^	11.04 ± 0.17^Ec^	-	-
	1–6	14.86 ± 0.35^BCDa^	13.56 ± 0.17^Fb^	14.61 ± 0.23^BCab^	-	-
	1.5–2	15.58 ± 0.17^BCa^	14.44 ± 0.23^CDEb^	15.16 ± 0.29^ABab^	-	-
	1.5–4	14.74 ± 0.29^CDa^	13.77 ± 0.23^EFa^	12.72 ± 0.17^Db^	-	-
	1.5–6	15.16 ± 0.17^BCDa^	14.57 ± 0.059^CDa^	13.31 ± 0.17^Db^	-	-
Gelatin	0–2	16.96 ± 0.11^ABCa^	15.54 ± 0.11^BCb^	12.6 ± 0.23^EFc^	-	-
	0–4	16.50 ± 0.05^Ca^	13.77 ± 0.23^EFb^	-	-	-
	0–6	16.88 ± 0.11^ABCa^	13.52 ± 0.11^Fb^	12.86 ± 0.20^DEFc^	-	-
	1–2	17.05 ± 0.11^ABCa^	16.04 ± 0.11^ABb^	14.95 ± 0.23^Ac^	14.19 ± 0.11^Ac^	12.39 ± 0.29^Ad^
	1–4	17.38 ± 0.11^ABa^	16.17 ± 0.17^ABb^	14.40 ± 0.17^ABc^	-	-
	1–6	16.84 ± 0.17^ABCa^	16.8 ± 0.11^Aa^	14.19 ± 0.11^ABCb^	-	-
	2–2	17.55 ± 0.11^Aa^	13.77 ± 0.11^EFb^	12.93 ± 0.23^DEFc^	13.69 ± 0.11^ABb^	13.02 ± 0.11^Ac^
	2–4	17.09 ± 0.17^ABCa^	14.02 ± 0.11^EFb^	12.43 ± 0.47^Fc^	13.35 ± 0.11^Bbc^	12.82 ± 0.14^Abc^
	2–6	15.20 ± 0.11^Da^	13.69 ± 0.11^Fb^	12.72 ± 0.17^DEFc^	-	-
	3–2	16.38 ± 0.11^Ca^	14.53 ± 0.1^DEb^	13.52 ± 0.11^BCDc^	12.09 ± 0.23^Cd^	11.08 ± 0.23^Be^
	3–4	16.67 ± 0.17^BCa^	14.19 ± 0.11^EFb^	13.35 ± 0.11^CDEc^	11.55 ± 0.29^Cd^	-
	3–6	16.8 ± 0.47^BCa^	15.12 ± 0.47^CDb^	14.44 ± 0.23^Abc^	13.53 ± 0.12^ABc^	-
Pectin	0–2	16.96 ± 0.11^Aa^	15.54 ± 0.11^Ab^	12.6 ± 0.23^Dc^	-	-
	0–4	16.50 ± 0.05^ABa^	13.77 ± 0.23^CDb^	-	-	-
	0–6	16.88 ± 0.11^Aa^	13.52 ± 0.11^Db^	12.86 ± 0.20^CDc^	-	-
	1–2	15.87 ± 0.11^BCa^	14.16 ± 0.17^BCDb^	-	-	-
	1–4	15.036 ± 0.11^Da^	14.028 ± 0.11^BCDb^	13.90 ± 0.17^Bb^	11.92 ± 0.23^Ac^	-
	1–6	15.37 ± 0.11^CDa^	14.23 ± 0.17^BCDb^	13.35 ± 0.11^BCc^	11.84 ± 0.23^Ad^	-
	2–2	16.71 ± 0.11^Aa^	14.95 ± 0.23^ABb^	13.60 ± 0.11^Bc^	12.72 ± 0.17^Ad^	-
	2–4	15.28 ± 0.11^CDa^	14.78 ± 0.23^ABCa^	-	-	-
	2–6	14.78 ± 0.11^Da^	14.20 ± 0.11^BCDa^	-	-	-
	3–2	16.96 ± 0.23^Aa^	15.12 ± 0.23^ABb^	14.7 ± 0.11^Ab^	-	-
	3–4	16.71 ± 0.35^Aa^	14.61 ± 0.23^ABCDb^	13.52 ± 0.11^Bc^	-	-
	3–6	16.63 ± 0.23^Aa^	15 ± 0.23^ABb^	-	-	-

A–G: Uppercase letters within the same column indicate comparisons among cheese varieties across storage periods.

a–d: Lowercase letters within the same row indicate comparisons among samples at different storage periods. Different letters indicate statistically significant differences between samples, whereas identical letters indicate no statistically significant difference.

**-**: Because of the cheese samples had melted, analysis did not be performed.

Salt analysis was performed on white cheese samples with different stabilizers added to their brines, and the results are presented in Table 5. Salt values for cheese samples increased compared to the beginning of storage. The highest salt value was observed in the sample containing xanthan, and the lowest in the sample containing pectin. The lowest salt value was measured as 0.79% on day 1 in both the cheese with 1% xanthan gum and 2% salt and the cheese with 1% pectin and 2% salt in their brines. The highest salt value was measured as 3.45% on day 30 in the cheese with 2% gelatin and 6% salt in its brine.

**Table 5:** Salt values measured during storage in white cheese products containing different stabilizers and salts in their brine (%).

Stabilizer	Stabilizer % - Salt %	1st day	15th day	30th day	60th day	90th day
Carrageenan gum	0–2	0.86 ± 0^Db^	1.15 ± 0^EFa^	1.22 ± 0.10^Da^	-	-
	0–4	1.44 ± 0^Ba^	1.44 ± 0^DEa^	-	-	-
	0–6	1.73 ± 0^Aa^	1.72 ± 0^BCDa^	1.76 ± 0.1^BCDa^	-	-
	0.5–2	0.86 ± 0^Dc^	0.86 ± 0^Fc^	1.15 ± 0^Db^	1.29 ± 0^Ca^	-
	0.5–4	1.22 ± 0.10^Ca^	1.44 ± 0^DEa^	1.40 ± 0.0^CDa^	-	-
	0.5–6	1.44 ± 0^Ba^	2.16 ± 0.20^ABa^	2.05 ± 0.1^BCa^	-	-
	1–2	0.86 ± 0^Dd^	1.29 ± 0^DEFb^	1.32 ± 0^Dc^	1.44 ± 0^BCa^	1.15 ± 0^Cc^
	1–4	1.15 ± 0^Cb^	1.65 ± 0.10^CDa^	1.58 ± 0^BCDa^	1.51 ± 0.10^Ba^	1.72 ± 0^Ba^
	1–6	1.44 ± 0^Bb^	2.016 ± 0.20^ABCab^	2.20 ± 0.1^Ba^	-	-
	1.5–2	0.86 ± 0^Db^	0.93 ± 0.10^Fb^	1.37 ± 0.10^CDa^	1.29 ± 0^Ca^	1.36 ± 0.10^Ca^
	1.5–4	1.08 ± 0.10^Cc^	1.58 ± 0.20^CDEb^	1.66 ± 0.10^BCDab^	1.87 ± 0^Aab^	2.08 ± 0.10^Aa^
	1.5–6	1.44 ± 0^Bb^	2.23 ± 0.10^Aab^	3.168 ± 0.40^Aa^	-	-
Xanthan gum	0–2	0.86 ± 0^EFb^	1.15 ± 0^Ea^	1.22 ± 0.10^Fa^	-	-
	0–4	1.44 ± 0^BCDa^	1.44 ± 0^BCDEa^	-	-	-
	0–6	1.73 ± 0^ABCa^	1.72 ± 0^BCa^	1.76 ± 0.1^DEa^	-	-
	0.5–2	1.00 ± 0.20^DEFa^	1.15 ± 0^Ea^	1.29 ± 0.20^EFa^	-	-
	0.5–4	1.29 ± 0.20^CDEFb^	1.58 ± 0^BCDab^	1.94 ± 0.10^CDa^	-	-
	0.5–6	1.87 ± 0.20^ABb^	2.66 ± 0.10^Aa^	2.44 ± 0.20^Bab^	-	-
	1–2	0.79 ± 0.30^Fa^	1.29 ± 0.20^DEa^	1.15 ± 0^Fa^	-	-
	1–4	1.36 ± 0.10^BCDEb^	1.8 ± 0.10^Ba^	1.94 ± 0.10^CDa^	-	-
	1–6	2.01 ± 0^Ab^	2.80 ± 0.10^Aa^	2.59 ± 0^Ba^	-	-
	1.5–2	0.93 ± 0.10^DEFb^	1.36 ± 0.10^CDEa^	1.44 ± 0^EFa^	-	-
	1.5–4	1.44 ± 0^BCDb^	1.8 ± 0.10^Ba^	2.37 ± 0.10^BCa^	-	-
	1.5–6	2.08 ± 0.10^Ac^	2.80 ± 0.10^Ab^	3.31 ± 0.20^Aa^	-	-
Gelatin	0–2	0.86 ± 0^Eb^	1.15 ± 0^FGa^	1.22 ± 0.10^Fa^	-	-
	0–4	1.44 ± 0^ABCDa^	1.44 ± 0^DEa^	-	-	-
	0–6	1.73 ± 0^ABa^	1.72 ± 0^Ca^	1.76 ± 0.1^Da^	-	-
	1–2	1.08 ± 0.20^DEb^	1.08 ± 0^Gb^	1.44 ± 0^EFab^	1.08 ± 0.10^Bab^	1.51 ± 0.10^Aa^
	1–4	1.44 ± 0^ABCDab^	1.29 ± 0^EFb^	1.65 ± 0.10^DEa^	-	-
	1–6	1.87 ± 0.20^Aa^	2.37 ± 0.10^Aa^	1.87 ± 0^CDa^	-	-
	2–2	1.08 ± 0.20^DEb^	1.22 ± 0.10^Fab^	1.44 ± 0^EFab^	1.29 ± 0^Bab^	1.65 ± 0.10^Aa^
	2–4	1.36 ± 0.10^BCDd^	1.51 ± 0.10^Dcd^	2.01 ± 0^Ca^	1.72 ± 0^Ab^	1.87 ± 0^Aab^
	2–6	1.72 ± 0^ABc^	2.44 ± 0^Ab^	3.45 ± 0^Aa^	-	-
	3–2	1.08 ± 0.10^CDEa^	1.15 ± 0^FGa^	1.44 ± 0^EFa^	1.15 ± 0^Ba^	1.44 ± 0^Aa^
	3–4	1.51 ± 0.10^ABCab^	1.58 ± 0^CDab^	1.80 ± 0.10^CDa^	1.29 ± 0^Bb^	
	3–6	1.72 ± 0^ABc^	2.16 ± 0^Bb^	2.80 ± 0.10^Ba^	1.65 ± 0.10^Ac^	-
Pectin	0–2	0.86 ± 0^Db^	1.15 ± 0^FGa^	1.22 ± 0.10^CDa^	-	-
	0–4	1.44 ± 0^Ba^	1.44 ± 0^DEa^	-	-	-
	0–6	1.73 ± 0^Aa^	1.72 ± 0^Ca^	1.76 ± 0.1^Ba^	-	-
	1–2	0.79 ± 0.10^Da^	1.08 ± 0^Ga^	-	-	-
	1–4	0.86 ± 0^Dc^	1.29 ± 0^EFb^	1.22 ± 0.10^CDb^	1.65 ± 0.10^ABa^	-
	1–6	1.15 ± 0^Cb^	1.51 ± 0.10^Dab^	1.51 ± 0.10^BCab^	2.01 ± 0.20^Aa^	-
	2–2	0.86 ± 0^Db^	1.08 ± 0^Gb^	1.08 ± 0^Db^	1.36 ± 0.10^Ba^	-
	2–4	1.15 ± 0^Cb^	1.8 ± 0.10^Ca^	-	-	-
	2–6	1.44 ± 0^Bc^	2.30 ± 0^Bb^	2.80 ± 0^Aa^	-	-
	3–2	0.86 ± 0^Db^	1.15 ± 0^FGa^	-	-	-
	3–4	1.08 ± 0.10^Cb^	1.29 ± 0^EFab^	1.51 ± 0.10^BCa^	-	-
	3–6	1.72 ± 0^Ab^	2.52 ± 0.10^Aa^	-	-	-

A–G: Uppercase letters within the same column indicate comparisons among cheese varieties across storage periods.

a–d: Lowercase letters within the same row indicate comparisons among samples at different storage periods. Different letters indicate statistically significant differences between samples, whereas identical letters indicate no statistically significant difference.

**-**: Because of the cheese samples had melted, analysis did not be performed.

pH analysis was performed on white cheese samples with different stabilizers added to their brines, and the results are presented in Table 6. The pH values of all samples decreased throughout storage. While the highest pH value was measured in samples containing carrageenan, the lowest was observed in samples containing pectin. The lowest pH value was measured as 4.74 on day 90 in the cheese with 1.5% carrageenan gum and 4% salt in its brine. The highest pH value was measured as 5.63 on day 15 in the cheese with 0.5% xanthan and 6% salt in its brine.

**Table 6:** pH values measured during storage in white cheese products containing different stabilizers and salts in their brine.

Stabilizer	Stabilizer % - Salt %	1st day	15th day	30th day	60th day	90th day
Carrageenan gum	0–2	5.22 ± 0.01^Da^	5.17 ± 0.01^Ca^	5.11 ± 0.01^DEb^	-	-
	0–4	5.15 ± 0.00^Ea^	5.29 ± 0.00^Aa^	-	-	-
	0–6	5.28 ± 0.01^Ba^	5.24 ± 0.01^Bab^	5.19 ± 0.01^ABb^	-	-
	0.5–2	5.28 ± 0.00^Ba^	5.25 ± 0.01^Ba^	5.17 ± 0.02^BCb^	5.03 ± 0.01^Ac^	-
	0.5–4	5.32 ± 0.00^Aa^	5.14 ± 0.00^CDc^	5.18 ± 0.01^ABCb^	-	-
	0.5–6	5.33 ± 0.00^Aa^	5.24 ± 0.00^Bb^	5.23 ± 0.01^Ab^	-	-
	1–2	5.25 ± 0.01^Ca^	5.03 ± 0.01^Fc^	5.16 ± 0.01^BCDb^	5.01 ± 0.02^Ac^	4.98 ± 0.02^Ac^
	1–4	5.17 ± 0.01^Ea^	5.14 ± 0.01^Da^	5.00 ± 0.01^Gb^	4.99 ± 0.02^Ab^	4.95 ± 0.02^Ab^
	1–6	5.16 ± 0.01^Ea^	5.17 ± 0.01^Ca^	5.14 ± 0.01^CDa^	-	-
	1.5–2	5.10 ± 0.01^Ga^	5.07 ± 0.01^Ea^	4.96 ± 0.01^Ga^	4.92 ± 0.01^Ba^	4.91 ± 0.13^Aa^
	1.5–4	5.13 ± 0.01^Fa^	5.15 ± 0.01^CDa^	5.08 ± 0.01^EFb^	4.92 ± 0.01^Bc^	4.74 ± 0.01^Ad^
	1.5–6	5.21 ± 0.00^Da^	5.10 ± 0.01^Eb^	5.06 ± 0.01^Fc^	-	-
Xanthan gum	0–2	5.22 ± 0.01^BCa^	5.17 ± 0.01^Ja^	5.11 ± 0.01^Fb^	-	-
	0–4	5.15 ± 0.00^Cb^	5.29 ± 0.00^GHa^	-	-	-
	0–6	5.28 ± 0.01^ABCa^	5.24 ± 0.01^Iab^	5.19 ± 0.01^Eb^	-	-
	0.5–2	5.21 ± 0.00^BCc^	5.31 ± 0.02^FGb^	5.38 ± 0.01^Ca^	-	-
	0.5–4	5.39 ± 0.01^Ab^	5.46 ± 0.01^Ca^	5.46 ± 0.01^Ba^	-	-
	0.5–6	5.24 ± 0.01^ABCc^	5.63 ± 0.00^Aa^	5.55 ± 0.01^Ab^	-	-
	1–2	5.23 ± 0.01^ABCc^	5.34 ± 0.00^DEb^	5.39 ± 0.01^Ca^	-	-
	1–4	5.23 ± 0.01^ABCc^	5.33 ± 0.01^EFb^	5.37 ± 0.01^Ca^	-	-
	1–6	5.23 ± 0.01^ABCc^	5.37 ± 0.00^Db^	5.52 ± 0.01^Aa^	-	-
	1.5–2	5.16 ± 0.01^Cc^	5.35 ± 0.01^DEa^	5.30 ± 0.01^Db^	-	-
	1.5–4	5.37 ± 0.14^ABa^	5.27 ± 0.01^HIa^	5.39 ± 0.01^Ca^	-	-
	1.5–6	5.20 ± 0.00^Cc^	5.55 ± 0.00^Ba^	5.39 ± 0.01^Cb^	-	-
Gelatin	0–2	5.22 ± 0.01^Ca^	5.17 ± 0.01^Ea^	5.11 ± 0.01^Bb^	-	-
	0–4	5.15 ± 0.00^Db^	5.29 ± 0.00^Aa^	-	-	-
	0–6	5.28 ± 0.01^Aa^	5.24 ± 0.01^BCDab^	5.19 ± 0.01^Ab^	-	-
	1–2	5.25 ± 0.01^Ba^	5.17 ± 0.01^Eab^	5.04 ± 0.01^Cabc^	4.91 ± 0.17^Abc^	4.83 ± 0.02^Ac^
	1–4	5.23 ± 0.01^BCa^	5.21 ± 0.01^Da^	5.11 ± 0.01^Bb^	-	-
	1–6	5.24 ± 0.01^BCa^	5.27 ± 0.01^ABa^	5.08 ± 0.02^BCb^	-	-
	2–2	5.14 ± 0.01^DEa^	5.13 ± 0.01^FGa^	5.03 ± 0.01^Ca^	4.98 ± 0.01^Aa^	4.90 ± 0.16^Aa^
	2–4	5.16 ± 0.01^Da^	5.16 ± 0.01^EFa^	5.04 ± 0.01^Cb^	5.08 ± 0.01^Aab^	4.93 ± 0.04^Ac^
	2–6	5.22 ± 0.01^Cb^	5.25 ± 0.01^BCa^	5.12 ± 0.01^Bc^	-	-
	3–2	5.12 ± 0.01^Ea^	5.15 ± 0.01^EFa^	5.08 ± 0.01^BCa^	4.93 ± 0.13^Aa^	4.89 ± 0.18^Aa^
	3–4	5.09 ± 0.01^Fa^	5.11 ± 0.01^Ga^	5.10 ± 0.01^Ba^	5.10 ± 0.01^Aa^	-
	3–6	5.24 ± 0.01^BCa^	5.23 ± 0.01^CDa^	5.20 ± 0.01^Aa^	5.13 ± 0.02^Ab^	-
Pectin	0–2	5.22 ± 0.01^Ba^	5.17 ± 0.01^DEa^	5.11 ± 0.01^Bb^	-	-
	0–4	5.15 ± 0.00^Cb^	5.29 ± 0.00^Aa^	-	-	-
	0–6	5.28 ± 0.01^Aa^	5.24 ± 0.01^Bab^	5.19 ± 0.01^Ab^	-	-
	1–2	5.09 ± 0.01^DEb^	5.17 ± 0.01^DEa^		-	-
	1–4	5.08 ± 0.01^EFc^	5.16 ± 0.00^DEb^	5.23 ± 0.00^Aa^	5.06 ± 0.01^Ad^	-
	1–6	5.09 ± 0.00^DEa^	5.09 ± 0.01^Fa^	5.00 ± 0.00^Cb^	4.90 ± 0.01^Cc^	-
	2–2	5.06 ± 0.01^FGc^	5.19 ± 0.01^CDa^	5.09 ± 0.00^Bb^	5.01 ± 0.00^Bd^	-
	2–4	5.12 ± 0.01^Db^	5.22 ± 0.01^BCa^		-	-
	2–6	5.12 ± 0.00^CDb^	5.28 ± 0.00^Aa^		-	-
	3–2	5.03 ± 0.00^Gb^	5.15 ± 0.01^Ea^	5.13 ± 0.01^Ba^	-	-
	3–4	5.06 ± 0.01^FGc^	5.16 ± 0.01^Eb^	5.22 ± 0.00^Aa^	-	-
	3–6	5.08 ± 0.01^EFb^	5.30 ± 0.01^Aa^		-	-

A–G: Uppercase letters within the same column indicate comparisons among cheese varieties across storage periods.

a–d: Lowercase letters within the same row indicate comparisons among samples at different storage periods. Different letters indicate statistically significant differences between samples, whereas identical letters indicate no statistically significant difference.

**-**: Because of the cheese samples had melted, analysis did not be performed.

Total acidity analysis was performed on white cheese samples with different stabilizers added to their brines, and the results are presented in Table 7. Titratable acidity did not show a regular pattern of change in the samples. In some samples, a decrease followed by an increase was observed, while in others a continuous increase was seen. The lowest total acidity value was measured as 0.81% on day 30 in the cheese with 1.5% xanthan and 4% salt in its brine. The highest total acidity value was measured as 2.03% on day 90 in the cheese with 2% gelatin and 4% salt in its brine.

**Table 7:** Acidity values measured during storage in white cheese products containing different stabilizers and salts in their brine (%).

	Stabilizer % - Salt %	1st day	15th day	30th day	60th day	90th day
Carrageenan gum	0–2	1.24 ± 0.01^Db^	1.28 ± 0.01^CDab^	1.34 ± 0.02^Ca^	-	-
	0–4	1.06 ± 0.01^Eb^	1.13 ± 0.01^Ea^	-	-	-
	0–6	1.36 ± 0.01^Ab^	1.42 ± 0.01^Aab^	1.48 ± 0.01^Ba^	-	-
	0.5–2	1.28 ± 0.01^CDc^	1.17 ± 0.05^Ed^	1.60 ± 0.01^Ab^	1.73 ± 0.01^ABa^	-
	0.5–4	1.25 ± 0.01^Da^	1.16 ± 0.01^Eb^	1.19 ± 0.01^Db^	-	-
	0.5–6	1.29 ± 0.01^CDb^	1.40 ± 0.01^ABa^	1.48 ± 0.04^Ba^	-	-
	1–2	1.34 ± 0.02^ABCd^	1.10 ± 0^Ee^	1.39 ± 0.01^Cc^	1.75 ± 0.01^Ab^	1.87 ± 0.01^Aa^
	1–4	1.35 ± 0.00^ABd^	1.19 ± 0.02^DEe^	1.53 ± 0.02^Ac^	1.61 ± 0.01^Bb^	1.77 ± 0.01^Ba^
	1–6	1.29 ± 0.01^BCDb^	1.30 ± 0.06^BCb^	1.56 ± 0.01^Aa^	-	-
	1.5–2	1.37 ± 0.03^Ac^	1.16 ± 0.01^Ed^	1.40 ± 0.01^Cc^	1.65 ± 0.07^ABb^	1.88 ± 0.01^Aa^
	1.5–4	1.37 ± 0.01^Ac^	1.20 ± 0^CDEd^	1.38 ± 0.04^Cc^	1.77 ± 0.01^Aa^	1.65 ± 0.01^Cb^
	1.5–6	1.25 ± 0.03^Db^	1.45 ± 0^Aa^	1.51 ± 0.01^Ba^	-	-
Xanthan gum	0–2	1.24 ± 0.01^Bb^	1.28 ± 0.01^Bab^	1.34 ± 0.02^Ba^	-	-
	0–4	1.06 ± 0.01^CDb^	1.13 ± 0.01^CDa^	-	-	-
	0–6	1.36 ± 0.01^Ab^	1.42 ± 0.01^Aab^	1.48 ± 0.01^Aa^	-	-
	0.5–2	1.03 ± 0.01^DEa^	1.12 ± 0.05^DEa^	1.00 ± 0.04^EFa^	-	-
	0.5–4	0.99 ± 0.02^EFb^	0.92 ± 0.01^Fb^	1.14 ± 0.02^CDa^	-	-
	0.5–6	1.00 ± 0.01^DEFa^	0.83 ± 0.03^Gb^	1.06 ± 0.01^DEa^	-	-
	1–2	0.94 ± 0.01^FGb^	1.04 ± 0.00^Ea^	0.91 ± 0.03^FGb^	-	-
	1–4	0.90 ± 0.03^GHc^	1.05 ± 0.03^DEb^	1.16 ± 0.00^Ca^	-	-
	1–6	1.12 ± 0.01^Ca^	1.10 ± 0.01^DEa^	0.86 ± 0.02^GHb^	-	-
	1.5–2	1.04 ± 0.01^DEb^	0.90 ± 0.01^FGc^	1.30 ± 0.03^Ba^	-	-
	1.5–4	0.83 ± 0.03^Hb^	1.21 ± 0.01^BCa^	0.81 ± 0.00^Hb^	-	-
	1.5–6	1.01 ± 0.03^DEFb^	0.90 ± 0.00^FGc^	1.16 ± 0.01^Ca^	-	-
Gelatin	0–2	1.24 ± 0.01^Ab^	1.28 ± 0.01^Aab^	1.34 ± 0.02^Ea^	-	-
	0–4	1.06 ± 0.01^Ab^	1.13 ± 0.01^Aa^	-	-	-
	0–6	1.36 ± 0.01^Ab^	1.42 ± 0.01^Aab^	1.48 ± 0.01^Da^	-	-
	1–2	1.43 ± 0.01^Acd^	1.26 ± 0.01^Ad^	1.62 ± 0.01^Abc^	1.80 ± 0.14^Aab^	1.93 ± 0.04^Aa^
	1–4	1.20 ± 0.01^Ac^	1.41 ± 0.01^Ab^	1.53 ± 0.01^CDa^	-	-
	1–6	0.87 ± 0.74^Aa^	0.87 ± 0.72^Aa^	1.56 ± 0.01^BCa^	-	-
	2–2	1.37 ± 0.01^Ab^	1.47 ± 0.01^Ab^	1.51 ± 0.01^CDb^	1.88 ± 0.04^Aa^	1.94 ± 0.19^Aa^
	2–4	1.42 ± 0.01^Ac^	1.47 ± 0.01^Ac^	1.48 ± 0.02^Dc^	1.94 ± 0.01^Ab^	2.03 ± 0.03^Aa^
	2–6	1.33 ± 0.01^Ab^	1.52 ± 0.01^Aa^	1.49 ± 0.01^Da^	-	-
	3–2	1.36 ± 0.03^Ad^	1.37 ± 0.01^Ad^	1.61 ± 0.01^ABc^	1.73 ± 0.02^Ab^	1.85 ± 0.02^Aa^
	3–4	1.42 ± 0.01^Ac^	1.43 ± 0.01^Ac^	1.63 ± 0.01^Ab^	1.84 ± 0.02^Aa^	-
	3–6	1.36 ± 0.01^Ac^	1.39 ± 0.01^Ac^	1.62 ± 0.01^Ab^	1.80 ± 0.03^Aa^	-
Pectin	0–2	1.24 ± 0.01^DEb^	1.28 ± 0.01^Fab^	1.34 ± 0.02^Fa^	-	-
	0–4	1.06 ± 0.01^Gb^	1.13 ± 0.01^Ga^	-	-	-
	0–6	1.36 ± 0.01^BCb^	1.42 ± 0.01^DEab^	1.48 ± 0.01^Ea^	-	-
	1–2	1.28 ± 0.01^Db^	1.51 ± 0.01^BCa^	-	-	-
	1–4	1.30 ± 0.00^CDd^	1.42 ± 0.01^DEc^	1.59 ± 0.02^Db^	1.77 ± 0.02^Ba^	-
	1–6	1.41 ± 0.01^ABc^	1.51 ± 0.01^BCb^	1.70 ± 0.03^Ca^	1.76 ± 0.01^Ba^	-
	2–2	1.13 ± 0.01^FGd^	1.48 ± 0.01^CDc^	1.70 ± 0.01^Cb^	1.91 ± 0.03^Aa^	-
	2–4	1.24 ± 0.06^DEb^	1.52 ± 0.02^ABCa^	-	-	-
	2–6	1.19 ± 0.01^EFb^	1.41 ± 0.01^Ea^	-	-	-
	3–2	1.36 ± 0.01^BCc^	1.58 ± 0.04^Ab^	1.81 ± 0.01^Ba^	-	-
	3–4	1.49 ± 0.01^Ac^	1.56 ± 0.01^ABb^	1.95 ± 0.01^Aa^	-	-
	3–6	1.22 ± 0.03^DEc^	1.38 ± 0.01^Eb^	-	-	-

A–G: Uppercase letters within the same column indicate comparisons among cheese varieties across storage periods.

a–d: Lowercase letters within the same row indicate comparisons among samples at different storage periods. Different letters indicate statistically significant differences between samples, whereas identical letters indicate no statistically significant difference.

**-**: Because of the cheese samples had melted, analysis did not be performed.

While total acidity in cheeses shows variability during maturation, it was measured as 0.89–1.43% at the beginning of storage and 1.85–2.03% at the end of maturation. While starter culture in the samples caused an increase in total acidity, the decrease in dry matter and the transfer of developed acidity into the stabilized brine water also caused a decrease in total acidity.

Water activity analysis was performed on white cheese samples with different stabilizers added to their brines, and results are presented in Table 8. When aw values of the samples were examined, the lowest value on the first day was found to be 0.9252 in samples containing carrageenan gum and gelatin, while the highest value was also observed in the sample containing gelatin. At the end of storage, measurement could only be performed in samples containing carrageenan gum and gelatin; the lowest value was 0.9254 in the sample with 1.5% carrageenan gum and 2% salt, while the highest value was 0.9310 in the sample with 1% carrageenan gum and 2% salt.

**Table 8:** *a_w_* values measured during storage in white cheese products containing different stabilizers and salts in their brine.

	Stabilizer % - Salt %	1st day	15th day	30th day	60th day	90th day
Carrageenan gum	0–2	0.9264 ± 0.002^ABCDa^	0.9275 ± 0.01^ABa^	0.9266 ± 0^Aa^	-	-
	0–4	0.9263 ± 0.003^ABCa^	0.9272 ± 0^ABa^	-	-	-
	0–6	0.9261 ± 0.003^ABCa^	0.9271 ± 0^ABa^	0.9288 ± 0.0^Aa^	-	-
	0.5–2	0.9252 ± 0.001^Da^	0.9271 ± 0.01^Aa^	0.9262 ± 0.00^Aa^	0.9261 ± 0.00^Aa^	-
	0.5–4	0.9263 ± 0.004^BCDa^	0.9254 ± 0.00^ABa^	0.9265 ± 0.00^Aa^	-	-
	0.5–6	0.9265 ± 0.002^ABCDab^	0.9253 ± 0.00^ABb^	0.9267 ± 0.00^Aa^	-	-
	1–2	0.9266 ± 0.005^ABCDb^	0.9267 ± 0.00^ABb^	0.9264 ± 0.00^Ab^	0.9265 ± 0.00^Ab^	0.9310 ± 0.00^Aa^
	1–4	0.9267 ± 0.004^ABa^	0.9263 ± 0.00^ABa^	0.9266 ± 0.00^Aa^	0.927 ± 0.00^Aa^	0.9295 ± 0.00^Aa^
	1–6	0.9261 ± 0.001^ABCa^	0.9262 ± 0.00^ABab^	0.9254 ± 0.00^Ab^	-	-
	1.5–2	0.9253 ± 0.001^CDb^	0.9250 ± 0.00^ABb^	0.9252 ± 0.00^Ab^	0.9267 ± 0.00^Aa^	0.9254 ± 0.00^Bb^
	1.5–4	0.9267 ± 0.004^BCDab^	0.9261 ± 0.00^Bab^	0.9263 ± 0.00^Aa^	0.9263 ± 0.00^Aa^	0.9256 ± 0.00^Bb^
	1.5–6	0.9270 ± 0.004^Aa^	0.9263 ± 0.00^ABa^	0.9248 ± 0.00^Ab^	-	-
Xanthan gum	0–2	0.9264 ± 0.002^BCDa^	0.9275 ± 0.01^Ba^	0.9266 ± 0^Aa^	-	-
	0–4	0.9263 ± 0.003^ABCa^	0.9272 ± 0^Ba^	-	-	-
	0–6	0.9261 ± 0.003^ABCa^	0.9271 ± 0^Ba^	0.9288 ± 0.0^Aa^	-	-
	0.5–2	0.9262 ± 0.001^CDb^	0.9304 ± 0.00^Aa^	0.9262 ± 0.00^Ab^	-	-
	0.5–4	0.9263 ± 0.005^ABCa^	0.9273 ± 0.00^Ba^	0.9253 ± 0.00^Aa^	-	-
	0.5–6	0.9274 ± 0.002^ABCa^	0.9273 ± 0.00^Ba^	0.9264 ± 0.01^Aa^	-	-
	1–2	0.9276 ± 0.002^Aa^	0.9271 ± 0.00^Ba^	0.9272 ± 0.00^Aa^	-	-
	1–4	0.9273 ± 0.003^ABa^	0.9274 ± 0.00^Ba^	0.9263 ± 0.00^Aa^	-	-
	1–6	0.9262 ± 0.001^BCDb^	0.9283 ± 0.00^Ba^	0.9267 ± 0.00^Ab^	-	-
	1.5–2	0.9264 ± 0.001^BCDab^	0.9275 ± 0.00^Ba^	0.9255 ± 0.00^Ab^	-	-
	1.5–4	0.9255 ± 0.004^Db^	0.9283 ± 0.00^Ba^	0.9262 ± 0.00^Ab^	-	-
	1.5–6	0.9263 ± 0.001^CDa^	0.9271 ± 0.01^Ba^	0.9263 ± 0.01^Aa^	-	-
Gelatin	0–2	0.9264 ± 0.002^CDa^	0.9275 ± 0.01^Aa^	0.9266 ± 0^Aa^	-	-
	0–4	0.9263 ± 0.003^BCa^	0.9272 ± 0^Aa^	-	-	-
	0–6	0.9261 ± 0.003^BCa^	0.9271 ± 0^Aa^	0.9288 ± 0.0^Aa^	-	-
	1–2	0.9274 ± 0.001^ABa^	0.9270 ± 0.01^Aa^	0.9264 ± 0^Aa^	0.9264 ± 0^ABa^	0.9264 ± 0^Aa^
	1–4	0.9272 ± 0.001^ABa^	0.9261 ± 0^Aa^	0.9263 ± 0^Aa^	-	-
	1–6	0.9273 ± 0.004^Aa^	0.9263 ± 0^Ab^	0.9271 ± 0^Aab^	-	-
	2–2	0.9278 ± 0.004^Aa^	0.9262 ± 0.01^Aa^	0.9263 ± 0^Aa^	0.9255 ± 0^Ba^	0.9263 ± 0^Aa^
	2–4	0.9252 ± 0.002^Ea^	0.9262 ± 0.01^Aa^	0.9266 ± 0^Aa^	0.9262 ± 0^ABa^	0.9260 ± 0^Aa^
	2–6	0.9264 ± 0.001^CDEb^	0.9265 ± 0^Ab^	0.9272 ± 0^Aa^	-	-
	3–2	0.9253 ± 0.003^DEa^	0.9264 ± 0.01^Aa^	0.9270 ± 0^Aa^	0.9263 ± 0^ABa^	0.9262 ± 0^Aa^
	3–4	0.9262 ± 0.001^CDEa^	0.9266 ± 0^Aa^	0.9274 ± 0^Aa^	0.9261 ± 0^ABa^	-
	3–6	0.9261 ± 0.001^CDEa^	0.9264 ± 0^Aa^	0.9265 ± 0^Aa^	0.9285 ± 0^Aa^	-
Pectin	0–2	0.9264 ± 0.002^Aa^	0.9275 ± 0.01^Aa^	0.9266 ± 0^Aa^	-	-
	0–4	0.9263 ± 0.003^Aa^	0.9272 ± 0^Aa^	-	-	-
	0–6	0.9261 ± 0.003^Aa^	0.9271 ± 0^Aa^	0.9288 ± 0.0^Bb^	-	-
	1–2	0.9271 ± 0.003^Ba^	0.9260 ± 0^Da^	-	-	-
	1–4	0.9262 ± 0.004^Bb^	0.9260 ± 0^CDb^	0.9272 ± 0^Ba^	0.9253 ± 0^Ab^	-
	1–6	0.9263 ± 0.007^Ba^	0.9261 ± 0^BCa^	0.9271 ± 0^Ba^	0.9252 ± 0^Aa^	-
	2–2	0.9265 ± 0.003^Ba^	0.9262 ± 0^CDa^	0.9265 ± 0^Ba^	0.9255 ± 0^Aa^	-
	2–4	0.9264 ± 0.003^Ba^	0.9263 ± 0^CDa^	-	-	-
	2–6	0.9264 ± 0.003^Ba^	0.9272 ± 0^Ba^	-	-	-
	3–2	0.9268 ± 0.002^Ba^	0.9266 ± 0^CDa^	0.9267 ± 0^Ba^	-	-
	3–4	0.9265 ± 0.003^Ba^	0.9267 ± 0^BCa^	0.9271 ± 0^Ba^	-	-
	3–6	0.9267 ± 0.004^Ba^	0.9270 ± 0^Ba^	-	-	-

A–G: Uppercase letters within the same column indicate comparisons among cheese varieties across storage periods.

a–d: Lowercase letters within the same row indicate comparisons among samples at different storage periods. Different letters indicate statistically significant differences between samples, whereas identical letters indicate no statistically significant difference.

**-**: Because of the cheese samples had melted, analysis did not be performed.

Water activity values generally decreased. This was because the cheeses absorbed water over time and absorbed salt along with the water. Salt is among the factors that decrease water activity in foods. Concurrently, water-soluble nitrogen ratios increase as ripening progresses. Water-soluble nitrogen also contributes to a decrease in water activity in cheese. However, water activity did not decrease as much as expected because the cheese absorbed excess water during this period. Consequently, water activity changed upward again. For these reasons, water activity values followed a fluctuating course and these changes were found to be statistically significant (*p* < 0.05).

### 3.3 Color properties

Color analysis was performed on white cheese samples with different stabilizers added to their brines, and the results are presented in Tables 9–11. The L* values of the samples followed a fluctuating course throughout storage. The lowest L* value was measured at 71.82 in the 15-day analysis of the control white cheese with 6% salt in its brine. The highest L* value was measured at 94.59 in the day 1 analysis of the cheese with 1.5% xanthan and 6% salt in its brine. Examination of the color values of the white cheeses produced with the addition of different stabilizers and salt levels to their brine revealed fluctuations in the samples’ L* values throughout storage. It was determined that there was no statistically significant difference (*p* > 0.05) in L* values between samples on day 1 and at the end of storage. The highest value on day 1 of storage was 94.55 in the sample containing xanthan in its brine, while the lowest value at the end of storage was 73.00 in the sample containing carrageenan gum in its brine (Table 9).

**Table 9:** L* values measured during storage in white cheese products containing different stabilizers and salts in their brine.

	Stabilizer % - Salt %	1st day	15th day	30th day	60th day	90th day
Carrageenan gum	0–2	89.61 ± 0.19^Fa^	73.70 ± 0.00^Fc^	88.93 ± 0.00^BCDb^	-	-
	0–4	91.64 ± 0.11^Aa^	73.34 ± 0.04^Gb^	-	-	-
	0–6	89.88 ± 0.08^EFa^	71.82 ± 0.05^Hc^	87.36 ± 0.03^Fb^	-	-
	0.5–2	88.73 ± 0.07^Gb^	90.11 ± 0.01^CDa^	88.23 ± 0.44^Eb^	89.99 ± 0.02^Aa^	-
	0.5–4	88.07 ± 0.04^Hc^	90.02 ± 0.03^Da^	89.50 ± 0.35^Bb^	-	-
	0.5–6	90.53 ± 0.01^CDa^	89.87 ± 0.01^Eb^	88.32 ± 0.03^DEc^	-	-
	1–2	90.82 ± 0.06^BCa^	90.50 ± 0.01^Ab^	85.98 ± 0.05^Gd^	88.51 ± 0.03^Bc^	77.80 ± 0.01^Ae^
	1–4	91.14 ± 0.03^Ba^	90.36 ± 0.02^Bb^	86.06 ± 0.08^Gd^	87.41 ± 0.03^Dc^	73.00 ± 0.09^Ce^
	1–6	91.11 ± 0.01^Ba^	90.16 ± 0.00^Cb^	89.36 ± 0.30^BCc^	-	-
	1.5–2	90.24 ± 0.06^DEb^	90.43 ± 0.09^ABb^	91.02 ± 0.01^Aa^	88.49 ± 0.00^Bc^	73.22 ± 0.03^Cd^
	1.5–4	91.79 ± 0.04^Aa^	90.42 ± 0.01^ABb^	86.60 ± 0.29^Gd^	87.94 ± 0.23^Cc^	74.21 ± 0.024^Be^
	1.5–6	86.33 ± 0.07^Ic^	90.03 ± 0.01^CDa^	88.73 ± 0.02^CDEb^	-	-
Xanthan gum	0–2	89.61 ± 0.19^CDa^	73.70 ± 0.00^Hc^	88.93 ± 0.00^Ab^	-	-
	0–4	91.64 ± 0.11^Ba^	73.34 ± 0.04^Ib^	-	-	-
	0–6	89.88 ± 0.08^CDa^	71.82 ± 0.05^Jc^	87.36 ± 0.03^Ab^	-	-
	0.5–2	89.12 ± 0.30^DEFa^	90.30 ± 0.01^Fa^	87.66 ± 2.84^Aa^	-	-
	0.5–4	89.50 ± 0.59^CDEab^	90.12 ± 0.01^Ga^	88.61 ± 0.02^Ab^	-	-
	0.5–6	88.64 ± 0.01^Fb^	90.54 ± 0.01^CDa^	88.61 ± 0.00^Ac^	-	-
	1–2	88.64 ± 0.01^Fc^	91.07 ± 0.00^Ba^	90.44 ± 0.01^Ab^	-	-
	1–4	84.55 ± 0.10^Gc^	91.14 ± 0.00^Ba^	88.60 ± 0.04^Ab^	-	-
	1–6	90.12 ± 0.03^Cb^	90.39 ± 0.04^Ea^	88.53 ± 0.01^Ac^	-	-
	1.5–2	88.71 ± 0.07^EFb^	90.59 ± 0.00^Ca^	88.88 ± 0.04^Ab^	-	-
	1.5–4	88.64 ± 0.16^EFc^	90.47 ± 0.03^DEa^	89.54 ± 0.04^Ab^	-	-
	1.5–6	94.59 ± 0.18^Aa^	91.29 ± 0.01^Ab^	89.14 ± 0.04^Ac^	-	-
Gelatin	0–2	89.61 ± 0.19^Ea^	73.70 ± 0.00^Fc^	88.93 ± 0.00^BCb^	-	-
	0–4	91.64 ± 0.11^Ba^	73.34 ± 0.04^Gb^	-	-	-
	0–6	89.88 ± 0.08^Ea^	71.82 ± 0.05^Hc^	87.36 ± 0.03^DEFb^	-	-
	1–2	91.94 ± 0.00^Aa^	90.38 ± 0.06^Ac^	90.93 ± 0.06^Ab^	90.26 ± 0.21^Ac^	90.85 ± 0.02^Ab^
	1–4	91.00 ± 0.00^Ca^	88.10 ± 0.04^Eb^	87.18 ± 0.04^EFc^	-	-
	1–6	91.55 ± 0.01^Ba^	89.09 ± 0.03^Cb^	88.77 ± 0.93^BCb^	-	-
	2–2	90.54 ± 0.00^Da^	90.23 ± 0.00^Ab^	86.55 ± 0.10^Fe^	88.58 ± 0.01^Bd^	89.05 ± 0.01^Bc^
	2–4	90.68 ± 0.04^Da^	89.75 ± 0.06^Bab^	89.34 ± 0.01^Bbc^	86.81 ± 0.35^Dd^	88.80 ± 0.02^Bc^
	2–6	91.55 ± 0.02^Ba^	88.73 ± 0.01^Dc^	89.39 ± 0.00^Bb^	-	-
	3–2	91.69 ± 0.02^ABa^	89.67 ± 0.02^Bb^	88.14 ± 0.05^CDEc^	87.88 ± 0.03^BCd^	88.09 ± 0.00^Cc^
	3–4	90.80 ± 0.00^CDa^	88.45 ± 0.01^Dc^	89.39 ± 0.01^Bb^	87.77 ± 0.02^Cd^	-
	3–6	90.72 ± 0.00^Da^	89.78 ± 0.02^Bb^	88.46 ± 0.11^BCDc^	88.10 ± 0.03^BCc^	-
Pectin	0–2	89.61 ± 0.19^Da^	73.70 ± 0.00^Gc^	88.93 ± 0.00^Ab^	-	-
	0–4	91.64 ± 0.11^Aa^	73.34 ± 0.04^Hb^	-	-	-
	0–6	89.88 ± 0.08^Da^	71.82 ± 0.05^Ic^	87.36 ± 0.03^Bb^	-	-
	1–2	87.47 ± 0.08^Fb^	88.22 ± 0.08^Da^	-	-	-
	1–4	87.12 ± 0.03^Fc^	89.67 ± 0.04^Ba^	85.74 ± 0.07^Cd^	88.51 ± 0.01^Cb^	-
	1–6	90.91 ± 0.03^BCa^	87.91 ± 0.04^DEc^	87.31 ± 0.21^Bd^	88.76 ± 0.00^Bb^	-
	2–2	90.60 ± 0.16^Ca^	90.12 ± 0.01^Ab^	88.92 ± 0.03^Ac^	88.81 ± 0.00^Ac^	-
	2–4	91.06 ± 0.04^BCa^	88.81 ± 0.01^Cb^	-	-	-
	2–6	90.57 ± 0.21^Ca^	86.22 ± 0.06^Fb^	-	-	-
	3–2	91.18 ± 0.01^ABa^	87.76 ± 0.03^Eb^	83.46 ± 0.30^Dc^	-	-
	3–4	89.77 ± 0.26^Da^	89.05 ± 0.03^Cb^	88.76 ± 0.30^Ab^	-	-
	3–6	88.67 ± 0.11^Ea^	88.18 ± 0.05^Db^	-	-	-

A–G: Uppercase letters within the same column indicate comparisons among cheese varieties across storage periods.

a–d: Lowercase letters within the same row indicate comparisons among samples at different storage periods. Different letters indicate statistically significant differences between samples, whereas identical letters indicate no statistically significant difference.

**-**: Because of the cheese samples had melted, analysis did not be performed.

**Table 10:** a* values measured during storage in white cheese products containing different stabilizers and salts in their brine.

	Stabilizer % - Salt %	1st day	15th day	30th day	60th day	90th day
Carrageenan gum	0–2	−0.19 ± 0.04^Bb^	−0.17 ± 0.02^Aa^	−0.37 ± 0.01^BCDc^	-	-
	0–4	−0.14 ± 0.03^ABa^	−0.01 ± 0.52^Aa^	-	-	-
	0–6	−0.10 ± 0.02^Aa^	−0.01 ± 0.51^Aa^	−0.30 ± 0.00^ABa^	-	-
	0.5–2	−0.44 ± 0.00^DEb^	−0.43 ± 0.01^Ab^	−0.31 ± 0.04^ABCa^	−0.24 ± 0.01^Ba^	-
	0.5–4	−0.62 ± 0.01^Fb^	−0.46 ± 0.01^Aa^	−0.40 ± 0.02^DEa^	-	-
	0.5–6	−0.47 ± 0.01^Ea^	−0.44 ± 0.01^Aa^	−0.42 ± 0.03^DEa^	-	-
	1–2	−0.42 ± 0.01^DEc^	−0.40 ± 0.01^Ac^	−0.48 ± 0.01^Ed^	−0.18 ± 0.01^Aa^	−0.32 ± 0.00^Cb^
	1–4	−0.42 ± 0.01^DEb^	−0.43 ± 0.00^Ab^	−0.88 ± 0.01^Fc^	−0.23 ± 0.01^ABa^	−0.28 ± 0.01^Ba^
	1–6	−0.40 ± 0.00^Dab^	−0.46 ± 0.01^Ab^	−0.38 ± 0.00^BCDa^	-	-
	1.5–2	−0.33 ± 0.01^Cc^	−0.42 ± 0.01^Ae^	−0.26 ± 0.00^Ab^	−0.35 ± 0.00^Cd^	−0.14 ± 0.01^Aa^
	1.5–4	−0.42 ± 0.01^DEd^	−0.45 ± 0.00^Ae^	−0.39 ± 0.01^CDc^	−0.30 ± 0.01^Cb^	−0.27 ± 0.01^Ba^
	1.5–6	−0.42 ± 0.00^DEb^	−0.44 ± 0.01^Ab^	−0.38 ± 0.01^BCDa^	-	-
Xanthan gum	0–2	−0.19 ± 0.04^Ba^	−0.17 ± 0.02^Aa^	−0.37 ± 0.01^ABb^	-	-
	0–4	−0.14 ± 0.03^ABa^	−0.01 ± 0.52^Aa^	-	-	-
	0–6	−0.10 ± 0.02^Aa^	−0.01 ± 0.51^Aa^	−0.30 ± 0.00^ABa^	-	-
	0.5–2	−0.32 ± 0.01^Ca^	−0.49 ± 0.01^Ab^	−0.34 ± 0.00^ABa^	-	-
	0.5–4	−0.31 ± 0.01^Ca^	−0.44 ± 0.00^Ab^	−0.64 ± 0.00^ABc^	-	-
	0.5–6	−0.66 ± 0.00^Eb^	−0.46 ± 0.01^Aa^	−1.06 ± 0.00^Bc^	-	-
	1–2	−0.66 ± 0.00^Ec^	−0.39 ± 0.00^Ab^	−0.21 ± 0.01^ABa^	-	-
	1–4	−0.16 ± 0.01^ABa^	−0.42 ± 0.01^Ab^	−0.41 ± 0.01^ABb^	-	-
	1–6	−0.52 ± 0.00^Da^	−0.52 ± 0.00^Aa^	−0.64 ± 0.00^ABb^	-	-
	1.5–2	−0.33 ± 0.01^Ca^	−0.47 ± 0.01^Aa^	−0.59 ± 0.01^ABa^	-	-
	1.5–4	−0.37 ± 0.01^Cb^	−0.44 ± 0.00^Aa^	0.00 ± 0.78^Aa^	-	-
	1.5–6	−0.67 ± 0.01^Eb^	−0.41 ± 0.01^Aa^	−0.40 ± 0.01^ABc^	-	-
Gelatin	0–2	−0.19 ± 0.04^Bb^	−0.17 ± 0.02^Aa^	−0.37 ± 0.01^Dc^	-	-
	0–4	−0.14 ± 0.03^ABa^	−0.01 ± 0.52^Aa^	-	-	-
	0–6	−0.10 ± 0.02^Aa^	−0.01 ± 0.51^Aa^	−0.30 ± 0.00^CDa^	-	-
	1–2	−0.44 ± 0.01^Dc^	−0.24 ± 0.00^Ab^	−0.19 ± 0.01^CDa^	−0.19 ± 0.01^Ba^	−0.21 ± 0.02^Bb^
	1–4	−0.41 ± 0.01^CDc^	−0.13 ± 0.01^Ab^	0.28 ± 0.04^ABa^	-	-
	1–6	−0.42 ± 0.00^CDb^	−0.22 ± 0.01^Aa^	−0.45 ± 0.03^Db^	-	-
	2–2	−0.40 ± 0.00^CDd^	−0.05 ± 0.01^Aa^	−0.23 ± 0.00^CDc^	−0.20 ± 0.01^CDb^	−0.18 ± 0.01^Ab^
	2–4	−0.41 ± 0.01^CDd^	−0.04 ± 0.02^Aa^	−0.14 ± 0.01^CDb^	−0.30 ± 0.02^Dc^	−0.25 ± 0.02^Bc^
	2–6	−0.44 ± 0.00^Da^	−0.06 ± 0.03^Aa^	−0.03 ± 0.25^BCa^	-	-
	3–2	−0.45 ± 0.00^Dd^	0.04 ± 0.00^Ab^	0.35 ± 0.02^Aa^	−0.09 ± 0.00^Bc^	−0.15 ± 0.03^Ac^
	3–4	−0.43 ± 0.01^CDc^	−0.12 ± 0.01^Ab^	0.24 ± 0.02^AB^a	−0.45 ± 0.01^Ec^	-
	3–6	−0.37 ± 0.00^Cc^	−0.18 ± 0.00^Ab^	0.33 ± 0.04^Aa^	0.38 ± 0.00^Aa^	-
Pectin	0–2	−0.19 ± 0.04^Eb^	−0.17 ± 0.02^ABa^	−0.37 ± 0.01^Ec^	-	-
	0–4	−0.14 ± 0.03^BCDa^	−0.01 ± 0.52^ABa^	-	-	-
	0–6	−0.10 ± 0.02^ABCDa^	−0.01 ± 0.51^ABa^	−0.30 ± 0.00^Da^	-	-
	1–2	−0.34 ± 0.00^Fb^	−0.04 ± 0.01^ABa^	-	-	-
	1–4	−0.20 ± 0.00^Eb^	−0.01 ± 0.01^ABa^	−0.20 ± 0.00^Cb^	−0.37 ± 0.00^Ac^	-
	1–6	−0.17 ± 0.00^DEc^	0.19 ± 0.03^ABb^	0.62 ± 0.02^Aa^	−0.36 ± 0.00^Ad^	-
	2–2	−0.07 ± 0.01^ABa^	−0.13 ± 0.01^ABb^	−0.10 ± 0.00^Bab^	−0.34 ± 0.00^Ac^	-
	2–4	−0.08 ± 0.02^ABCb^	−0.32 ± 0.01^ABa^	-	-	-
	2–6	−0.03 ± 0.04^Aa^	−0.39 ± 0.01^Bb^	-	-	-
	3–2	−0.13 ± 0.01^BCDEb^	−0.70 ± 0.01^Aa^	−0.27 ± 0.01^Dc^	-	-
	3–4	−0.13 ± 0.01^BCDEb^	−0.01 ± 0.00^ABa^	-	-	-
	3–6	−0.16 ± 0.01^CDEb^	0.32 ± 0.01^ABa^	-	-	-

A–G: Uppercase letters within the same column indicate comparisons among cheese varieties across storage periods.

a–d: Lowercase letters within the same row indicate comparisons among samples at different storage periods. Different letters indicate statistically significant differences between samples, whereas identical letters indicate no statistically significant difference.

**-**: Because of the cheese samples had melted, analysis did not be performed.

**Table 11:** b* values measured during storage in white cheese products containing different stabilizers and salts in their brine.

	Stabilizer % - Salt %	1st day	15th day	30th day	60th day	90th day
Carrageenan gum	0–2	8.79 ± 0.30^Eb^	8.00 ± 0.13^Bc^	10.65 ± 0.01^BCa^	-	-
	0–4	9.21 ± 0.02^CDb^	9.44 ± 0.04^Aa^	-	-	-
	0–6	8.97 ± 0.14^DEab^	8.41 ± 0.54^Bb^	9.96 ± 0.02^DEa^	-	-
	0.5–2	9.40 ± 0.07^BCDb^	9.92 ± 0.00^Cc^	10.01 ± 0.06^CDEb^	11.82 ± 0.35^Ba^	-
	0.5–4	9.04 ± 0.01^CDEb^	9.73 ± 0.01^Cc^	9.55 ± 0.02^Ea^	-	-
	0.5–6	9.29 ± 0.03^CDb^	9.82 ± 0.00^Cc^	10.05 ± 0.03^DEa^	-	-
	1–2	9.11 ± 0.02^CDEd^	9.83 ± 0.00^Ce^	10.84 ± 0.01^Bb^	12.41 ± 0.06^Aa^	10.04 ± 0.00^Ac^
	1–4	10.23 ± 0.04^Ac^	9.91 ± 0.00^Ce^	10.74 ± 0.01^BCa^	10.45 ± 0.03^Db^	8.58 ± 0.04^Bd^
	1–6	10.20 ± 0.01^Ab^	9.75 ± 0.13^Cc^	9.93 ± 0.01^Aa^	-	-
	1.5–2	9.85 ± 0.01^ABc^	9.94 ± 0.04^Ce^	10.37 ± 0.01^BCDb^	10.68 ± 0.01^Da^	7.76 ± 0.01^Cd^
	1.5–4	9.29 ± 0.01^CDc^	10.06 ± 0.01^Ce^	10.38 ± 0.01^BCDb^	11.28 ± 0.01^Ca^	8.19 ± 0.01^Dd^
	1.5–6	9.39 ± 0.04^Cb^	10.87 ± 0.01^Cc^	12.21 ± 0.04^Aa^	-	-
Xanthan gum	0–2	8.79 ± 0.30^Cb^	8.00 ± 0.13^Bc^	10.65 ± 0.01^Ea^	-	-
	0–4	9.21 ± 0.02^BCb^	9.44 ± 0.04^Aa^	-	-	-
	0–6	8.97 ± 0.14^BCab^	8.41 ± 0.54^Bb^	9.96 ± 0.02^Fa^	-	-
	0.5–2	8.09 ± 0.11^Db^	8.95 ± 0.00^Cc^	11.36 ± 0.04^Da^	-	-
	0.5–4	7.88 ± 0.23^Db^	8.73 ± 0.01^Cc^	11.55 ± 0.16^CDa^	-	-
	0.5–6	11.41 ± 0.01^Aa^	8.79 ± 0.01^Cc^	10.71 ± 0.03^Eb^	-	-
	1–2	11.41 ± 0.01^Aa^	8.75 ± 0.00^Cb^	11.70 ± 0.20^Ca^	-	-
	1–4	9.33 ± 0.01^Bb^	8.77 ± 0.00^Cc^	12.48 ± 0.02^Ba^	-	-
	1–6	8.09 ± 0.01^Db^	9.02 ± 0.00^Cc^	11.83 ± 0.02^Ca^	-	-
	1.5–2	8.14 ± 0.01^Db^	8.86 ± 0.01^Cc^	11.80 ± 0.03^Ca^	-	-
	1.5–4	8.13 ± 0.06^Db^	8.85 ± 0.01^Cc^	10.93 ± 0.04^Ea^	-	-
	1.5–6	9.22 ± 0.01^BCb^	8.76 ± 0.00^Cc^	12.95 ± 0.04^Aa^	-	-
Gelatin	0–2	8.79 ± 0.30^Bb^	8.00 ± 0.13^Ec^	10.65 ± 0.01^FGa^	-	-
	0–4	9.21 ± 0.02^Ab^	9.44 ± 0.04^Da^	-	-	-
	0–6	8.97 ± 0.14^ABab^	8.41 ± 0.54^Eb^	9.96 ± 0.02^Ha^	-	-
	1–2	10.93 ± 0.01^Ce^	10.74 ± 0.06^Cb^	11.07 ± 0.04^EFa^	9.91 ± 0.04^Bd^	10.45 ± 0.03B^Cc^
	1–4	10.93 ± 0.01^Cc^	11.14 ± 0.01^BCb^	12.24 ± 0.23^BCa^	-	-
	1–6	10.86 ± 0.01^Cc^	11.45 ± 0.18^BCa^	10.31 ± 0.23^GHb^	-	-
	2–2	10.84 ± 0.00^Cd^	11.24 ± 0.07^BCb^	11.74 ± 0.01^CDa^	10.78 ± 0.03^Ac^	10.72 ± 0.12^ABc^
	2–4	10.93 ± 0.01^Cd^	11.19 ± 0.04^BCa^	11.51 ± 0.01^DEa^	10.72 ± 0.17^Ab^	10.15 ± 0.01^Cc^
	2–6	10.95 ± 0.01^Cc^	11.48 ± 0.14^Bb^	12.01 ± 0.16^CDa^	-	-
	3–2	11.04 ± 0.00^Cd^	11.35 ± 0.01^BCb^	12.71 ± 0.17^ABa^	10.25 ± 0.00^Bc^	11.10 ± 0.03^Ab^
	3–4	10.98 ± 0.00^Cd^	11.25 ± 0.05^BCb^	11.99 ± 0.12^CDa^	9.92 ± 0.00^Bc^	-
	3–6	10.86 ± 0.01^Cc^	12.63 ± 0.02^Aa^	12.85 ± 0.25^Aa^	10.70 ± 0.01^Ab^	-
Pectin	0–2	8.79 ± 0.30^Eb^	8.00 ± 0.13^Hc^	10.65 ± 0.01^Da^	-	-
	0–4	9.21 ± 0.02^Eb^	9.44 ± 0.04^Ga^	-	-	-
	0–6	8.97 ± 0.14^Eab^	8.41 ± 0.54^Hb^	9.96 ± 0.02^Ea^	-	-
	1–2	9.68 ± 0.01^Db^	13.40 ± 0.03^BCa^	-	-	-
	1–4	9.90 ± 0.03^Dd^	11.51 ± 0.00^Fa^	10.77 ± 0.03^Db^	10.59 ± 0.01^Cc^	-
	1–6	9.76 ± 0.02^Dd^	12.11 ± 0.06^EFb^	14.68 ± 0.08^Aa^	10.70 ± 0.01^Bc^	-
	2–2	9.89 ± 0.10^Dd^	12.64 ± 0.10^DEb^	13.03 ± 0.02^Ba^	10.92 ± 0.01^Ac^	-
	2–4	10.44 ± 0.13^BCb^	12.90 ± 0.04^CDa^	-	-	-
	2–6	10.85 ± 0.04^Bb^	11.76 ± 0.05^Fa^	-	-	-
	3–2	9.91 ± 0.02^Dc^	13.91 ± 0.11^Ba^	12.08 ± 0.07^Cb^	-	-
	3–4	10.02 ± 0.03^CDc^	12.81 ± 0.03^CDa^	11.85 ± 0.05^Cb^	-	-
	3–6	13.98 ± 0.04^Ab^	14.80 ± 0.03^Aa^	-	-	-

A–G: Uppercase letters within the same column indicate comparisons among cheese varieties across storage periods.

a–d: Lowercase letters within the same row indicate comparisons among samples at different storage periods. Different letters indicate statistically significant differences between samples, whereas identical letters indicate no statistically significant difference.

**-**: Because of the cheese samples had melted, analysis did not be performed.

The a* values, indicating redness, followed a fluctuating course throughout storage, as seen in Table 10. While the highest a* value on day 1 was −0.66 in the sample containing xanthan in its brine, the lowest value at the end of storage was −0.14 in the sample containing carrageenan gum in its brine. According to this, it can be said that the use of different stabilizers had significant effects on the a* value of cheese.

The b* value results for white cheese samples with different stabilizers added to their brines are presented in Table 11. These values followed a fluctuating course throughout storage. While the values of some samples continuously increased, others first increased and then decreased. The increase recorded in b* value throughout storage was also found to be statistically significant (*p* < 0.05). The increase in b* value indicates an increase in yellowness. Accordingly, it was determined that the yellowness of the samples increased over time and then decreased again. Values that were 7.85–13.98 on day 1 were found to be between 7.76 and 10.04 at the end of storage.

### 3.4 Microbiological properties

To examine the microbiological changes occurring during storage of cheeses produced with the gel brine technique, *Lactococcus, Lactobacillus*, total mesophile, and total yeast-mold analyses were performed on the samples. *Lactococcus* analyses were performed on white cheese samples with different stabilizers added to their brines, and the 90-day values for *Lactococcus* counts are presented in Table 12. The lowest *Lactococcus* count was measured as 3.18 log cfu/g in the day 1 analysis of the control group white cheese with 6% salt in its brine. The highest *Lactococcus* count was observed as 8.56 log cfu/g on day 90 in the cheese with 1% pectin and 2% salt in its brine. When the data are examined, it is understood that the *Lactococcus* counts of the samples showed fluctuating changes during storage and that this change did not follow a regular pattern.

**Table 12:** *Lactococcus* counts measured during storage in white cheese products containing different stabilizers and salts in their brine (log cfu/g).

	Stabilizer % - Salt %	1st day	15th day	30th day	60th day	90th day
Carrageenan gum	0–2	3.20 ± 0.04^Gc^	7.02 ± 0.03^B^	8.34 ± 0.03^Aa^	-	-
	0–4	3.46 ± 0.02^Fb^	6.60 ± 0.00^EFa^	-	-	-
	0–6	3.18 ± 0.04^Gc^	6.78 ± 0.00^Db^	7.30 ± 0.02^Ca^	-	-
	0.5–2	5.70 ± 0.00^Ed^	6.81 ± 0.05^CDb^	6.30 ± 0.00^Hc^	7.90 ± 0.02^Ba^	-
	0.5–4	7.36 ± 0.00^Aa^	6.70 ± 0.00^DEb^	6.48 ± 0.00^Gc^	-	-
	0.5–6	6.30 ± 0.00^Db^	6.54 ± 0.09^Fa^	6.48 ± 0.00^Gab^	-	-
	1–2	6.48 ± 0.00^Ce^	7.50 ± 0.03^Ac^	7.23 ± 0.00^Cd^	8.39 ± 0.07^Aa^	8.10 ± 0.02^Ab^
	1–4	6.41 ± 0.00^Cc^	6.74 ± 0.06^DEb^	7.26 ± 0.00^Ca^	7.35 ± 0.05^Ca^	7.25 ± 0.01^Ba^
	1–6	6.48 ± 0.00^Cb^	6.30 ± 0.00^Gc^	6.74 ± 0.06^Ea^	-	-
	1.5–2	5.70 ± 0.00^Ee^	6.93 ± 0.04^BCd^	7.41 ± 0.02^Bb^	8.54 ± 0.01^Aa^	7.29 ± 0.02^Bc^
	1.5–4	6.30 ± 0.00^Dc^	6.30 ± 0.00^Gc^	7.10 ± 0.02^Db^	7.10 ± 0.02^Db^	7.30 ± 0.02^Ba^
	1.5–6	6.90 ± 0.00^Ba^	6.70 ± 0.00^DEb^	6.60 ± 0.00^Fb^	-	-
Xanthan gum	0–2	3.20 ± 0.04^FGc^	7.02 ± 0.03^Eb^	8.34 ± 0.03^Aa^	-	-
	0–4	3.46 ± 0.02^Fb^	6.60 ± 0.00^Ga^	-	-	-
	0–6	3.18 ± 0.04^Gc^	6.78 ± 0.00^Fb^	7.30 ± 0.02^Fa^	-	-
	0.5–2	6.60 ± 0.00^Ac^	7.92 ± 0.04^Ab^	8.13 ± 0.02^BCa^	-	-
	0.5–4	6.15 ± 0.04^BCc^	7.15 ± 0.04^Db^	7.90 ± 0.08^DEa^	-	-
	0.5–6	6.39 ± 0.12^ABb^	6.48 ± 0.00^Hb^	8.22 ± 0.02^ABa^	-	-
	1–2	6.24 ± 0.09^BCb^	6.48 ± 0.00^Hb^	7.77 ± 0.10^Ea^	-	-
	1–4	6.06 ± 0.03^CDc^	7.67 ± 0.02^Bb^	8.32 ± 0.00^Aa^	-	-
	1–6	5.84 ± 0.09^Dc^	6.30 ± 0.00^Ib^	7.93 ± 0.04^DEa^	-	-
	1.5–2	6.28 ± 0.03^BCc^	7.00 ± 0.00^Eb^	8.27 ± 0.05^ABa^	-	-
	1.5–4	5.54 ± 0.09^Ec^	7.36 ± 0.03^Cb^	8.20 ± 0.00^ABa^	-	-
	1.5–6	5.54 ± 0.09^Ec^	6.74 ± 0.06^Fb^	8.00 ± 0.00^CDa^	-	-
Gelatin	0–2	3.20 ± 0.04^Hc^	7.02 ± 0.03^ABCDb^	8.34 ± 0.03^Aa^	-	-
	0–4	3.46 ± 0.02^Gb^	6.60 ± 0.00^Da^	-	-	-
	0–6	3.18 ± 0.04^Hc^	6.78 ± 0.00^BCDb^	7.30 ± 0.02^DEa^	-	-
	1–2	6.93 ± 0.04^Cb^	6.87 ± 0.04^BCDbc^	7.57 ± 0.02^Ca^	7.55 ± 0.02^Aa^	6.78 ± 0.00^Dc^
	1–4	7.30 ± 0.00^ABb^	7.09 ± 0.07^ABCDc^	7.87 ± 0.02^Ba^	-	-
	1–6	6.74 ± 0.06^Db^	6.93 ± 0.04^BCDab^	7.18 ± 0.10^Ea^	-	-
	2–2	7.28 ± 0.03^Ba^	7.26 ± 0.00^ABa^	7.13 ± 0.07^Ea^	7.25 ± 20.07^Ba^	7.18 ± 0.00^Ca^
	2–4	7.45 ± 0.03^Ab^	7.20 ± 0.00^ABCc^	7.60 ± 0.02^Ca^	7.28 ± 0.03^Bc^	7.72 ± 0.03^Aa^
	2–6	6.54 ± 0.09^Eb^	7.23 ± 0.00^ABCa^	7.28 ± 0.03^DEa^	-	-
	3–2	6.95 ± 0.00^Cc^	7.53 ± 0.00^Aa^	7.56 ± 0.03^Ca^	7.42 ± 0.02^ABb^	7.40 ± 0.02^Bb^
	3–4	6.70 ± 0.00^Da^	7.22 ± 0.45^ABCa^	7.45 ± 0.03^CDa^	7.25 ± 0.01^Ba^	-
	3–6	6.30 ± 0.00^Fb^	6.70 ± 0.00^CDa^	6.65 ± 0.07^Fa^	6.45 ± 0.02^Cb^	-
Pectin	0–2	3.20 ± 0.04^Fc^	7.02 ± 0.03^Bb^	8.34 ± 0.03^Aa^	-	-
	0–4	3.46 ± 0.02^Eb^	6.60 ± 0.00^FGa^	-	-	-
	0–6	3.18 ± 0.04^Fc^	6.78 ± 0.00^CDb^	7.30 ± 0.02^Ba^	-	-
	1–2	7.24 ± 0.02^Aa^	7.22 ± 0.02^Aa^	-	-	-
	1–4	7.13 ± 0.02^ABa^	6.78 ± 0.00^CDb^	6.90 ± 0.04^Cb^	7.10 ± 0.02^Ba^	-
	1–6	7.19 ± 0.02^Aa^	6.74 ± 0.06^DEc^	6.60 ± 0.00^Dd^	7.05 ± 0.02^Bb^	-
	2–2	6.65 ± 0.07^Db^	6.65 ± 0.07^EFb^	6.60 ± 0.00^Db^	8.50 ± 0.01^Aa^	-
	2–4	7.18 ± 0.00^Aa^	6.90 ± 0.00^BCb^	-	-	-
	2–6	6.74 ± 0.06^CDa^	6.48 ± 0.00^Gb^	-	-	-
	3–2	6.70 ± 0.00^Db^	6.48 ± 0.00^Gc^	6.90 ± 0.04^Ca^	-	
	3–4	7.02 ± 0.03^Ba^	6.74 ± 0.06^DEb^	6.60 ± 0.00^Db^	-	-
	3–6	6.87 ± 0.04^Cb^	7.00 ± 0.00^Ba^	-	-	-

A–G: Uppercase letters within the same column indicate comparisons among cheese varieties across storage periods.

a–d: Lowercase letters within the same row indicate comparisons among samples at different storage periods. Different letters indicate statistically significant differences between samples, whereas identical letters indicate no statistically significant difference.

**-**: Because of the cheese samples had melted, analysis did not be performed.

*Lactobacillus* analysis was performed on white cheese samples with different stabilizers added to their brines, and the 90-day values for *Lactobacillus* counts are presented in Table 13. The lowest *Lactobacillus* count was measured as 5.3 log cfu/g on day 1 of the cheese with 1.5% xanthan and 6% salt in its brine. The highest *Lactobacillus* count was measured as 7.6 log cfu/g on day 30 of the cheese with 1% gelatin and 4% salt in its brine. As with *Lactococcus* counts, *Lactobacillus* counts of the samples also showed fluctuating changes during storage without following a clear pattern. Total mesophilic aerobic bacteria analysis was performed on white cheese samples with different stabilizers added to their brines, and the 90-day values for total mesophilic aerobic bacteria counts are presented in Table 14. The lowest mesophilic aerobic bacteria count was measured as 5.39 log cfu/g on day 15 of the cheese with 3% pectin and 6% salt in its brine. The highest mesophilic aerobic bacteria count was measured as 8.41 log cfu/g on day 90 in the cheese with 1% gelatin and 2% salt in its brine. When the data are examined, it is understood that total mesophilic aerobic bacteria counts of the samples showed fluctuating changes during storage and these changes did not follow a regular pattern.

**Table 13:** *Lactobacillus* counts measured during storage in white cheese products containing different stabilizers and salts in their brine (log cfu/g).

	Stabilizer % - Salt %	1st day	15th day	30th day	60th day	90th day
Carrageenan gum	0–2	6.74 ± 0.06^Cb^	7.30 ± 0.00^Ba^	6.81 ± 0.05F^Gb^	-	-
	0–4	7.41 ± 0.00^Aa^	7.15 ± 0.00^BCb^	-	-	-
	0–6	7.06 ± 0.03^Bb^	7.54 ± 0.09^Aa^	7.30 ± 0.03^Aab^	-	-
	0.5–2	5.90 ± 0.00^Fc^	7.17 ± 0.08^BCa^	6.98 ± 0.03^DEb^	7.05 ± 0.02^Aab^	-
	0.5–4	6.72 ± 0.03^Ca^	6.70 ± 0.00^EFa^	6.74 ± 0.06^Ga^	-	-
	0.5–6	5.54 ± 0.09^Gb^	6.87 ± 0.04^DEa^	6.70 ± 0.00^Ga^	-	-
	1–2	5.54 ± 0.09^Gd^	7.57 ± 0.00^Aa^	7.16 ± 0.02^BCb^	6.65 ± 0.01^Cc^	6.70 ± 0.01^Cc^
	1–4	5.85 ± 0.00^Fc^	6.90 ± 0.00^Da^	6.90 ± 0.00^EFa^	5.60 ± 0.00^Ed^	6.65 ± 0.01^Db^
	1–6	6.40 ± 0.00^Dc^	6.65 ± 0.07^Fb^	7.26 ± 0.00^EBa^	-	-
	1.5–2	5.48 ± 0.00^Ge^	7.02 ± 0.03^CDb^	6.95 ± 0.00^DEc^	5.77 ± 0.00^Dd^	7.30 ± 0.00^Aa^
	1.5–4	6.11 ± 0.00^Ec^	7.04 ± 0.00^CDa^	7.06 ± 0.03^CDa^	6.95 ± 0.01^Bb^	6.90 ± 0.01^Bb^
	1.5–6	5.65 ± 0.07^Gb^	6.54 ± 0.09^Fa^	6.70 ± 0.00^Ga^	-	-
Xanthan gum	0–2	6.74 ± 0.06^Cb^	7.30 ± 0.00^BCa^	6.81 ± 0.05^Bb^	-	-
	0–4	7.41 ± 0.00^Ab^	7.15 ± 0.00^CDa^	-	-	-
	0–6	7.06 ± 0.03^Bb^	7.54 ± 0.09^Aa^	7.30 ± 0.03^Aab^	-	-
	0.5–2	6.19 ± 0.02^DEc^	7.46 ± 0.00^ABa^	6.65 ± 0.07^Bb^	-	-
	0.5–4	6.02 ± 0.17^EFGb^	7.16 ± 0.02^CDa^	6.59 ± 0.16^Bb^	-	-
	0.5–6	6.06 ± 0.03^EFc^	7.06 ± 0.03^Da^	6.54 ± 0.09^Bb^	-	-
	1–2	5.93 ± 0.04^FGc^	7.00 ± 0.00^DEa^	6.54 ± 0.09^Bb^	-	-
	1–4	5.65 ± 0.07^Hc^	7.36 ± 0.00^ABa^	6.48 ± 0.00^Bb^	-	-
	1–6	5.77 ± 0.10^GHb^	6.39 ± 0.12^Ga^	6.48 ± 0.00^Ba^	-	-
	1.5–2	6.35 ± 0.01^Da^	6.60 ± 0.00^Fa^	6.59 ± 0.16^Ba^	-	-
	1.5–4	5.60 ± 0.00^Hb^	6.87 ± 0.04^Ea^	6.75 ± 0.21^Ba^	-	-
	1.5–6	5.30 ± 0.00^Ic^	6.48 ± 0.00^FGb^	6.87 ± 0.04^Ba^	-	-
Gelatin	0–2	6.74 ± 0.06^DEFGb^	7.30 ± 0.00^Ba^	6.81 ± 0.05^EFb^	-	-
	0–4	7.41 ± 0.00^Ab^	7.15 ± 0.00^Ca^	-	-	-
	0–6	7.06 ± 0.03^BCb^	7.54 ± 0.09^Aa^	7.30 ± 0.03^Bab^	-	-
	1–2	6.87 ± 0.04^CDEFc^	6.93 ± 0.04^Ec^	7.52 ± 0.03^Aa^	7.23 ± 0.00^Ab^	6.95 ± 0.00^Bc^
	1–4	7.20 ± 0.04^ABb^	7.02 ± 0.03^DEc^	7.60 ± 0.00^Aa^	-	-
	1–6	6.98 ± 0.03^BCDb^	7.08 ± 0.00^CDa^	6.93 ± 0.04^Dab^	-	-
	2–2	6.90 ± 0.00^CDEc^	7.18 ± 0.00^Ca^	6.80 ± 0.00^b^	7.02 ± 0.00^Cd^	7.19 ± 0.06^Aa^
	2–4	6.70 ± 0.00^EFGe^	7.08 ± 0.00^CDa^	6.78 ± 0.00^FGb^	6.6 ± 0.00^Fc^	7.05 ± 0.02^ABd^
	2–6	6.59 ± 0.16^Gb^	7.10 ± 0.02^CDa^	7.15 ± 0.00^Ca^	-	-
	3–2	6.65 ± 0.07^FG^	7.46 ± 0.02^A^	7.24 ± 0.02^BC^	7.10 ± 0.01^B^	6.95 ± 0.01^AB^
	3–4	7.33 ± 0.01^A^	6.60 ± 0.00^F^	6.90 ± 0.00^DE^	6.80 ± 0.00^D^	-
	3–6	6.54 ± 0.09^G^	6.70 ± 0.00^F^	6.70 ± 0.00^G^	6.65 ± 0.01^E^	-
Pectin	0–2	6.74 ± 0.06^Eb^	7.30 ± 0.00^Ba^	6.81 ± 0.05^Bb^	-	-
	0–4	7.41 ± 0.00^Bb^	7.15 ± 0.00^Ba^	-	-	-
	0–6	7.06 ± 0.03^Cb^	7.54 ± 0.09^Aa^	7.30 ± 0.03^Aab^	-	-
	1–2	7.56 ± 0.01^Aa^	6.74 ± 0.06^Db^	-	-	-
	1–4	7.16 ± 0.02^Ca^	6.93 ± 0.04^Cb^	6.90 ± 0.02^Bb^	6.00 ± 0.00^Cc^	-
	1–6	6.74 ± 0.06^Eab^	6.54 ± 0.09^Ebc^	6.48 ± 0.00^Cc^	6.88 ± 0.00^Ba^	-
	2–2	6.85 ± 0.00^Db^	6.24 ± 0.02^FGc^	6.48 ± 0.00^Cc^	7.39 ± 0.12^Aa^	-
	2–4	6.70 ± 0.00^Ea^	6.10 ± 0.02^Gb^	-	-	-
	2–6	6.48 ± 0.00^Fa^	5.60 ± 0.00^Hb^	-	-	-
	3–2	6.70 ± 0.00^Ea^	6.60 ± 0.00^DEb^	6.30 ± 0.00^Dc^	-	-
	3–4	6.48 ± 0.00^Fa^	6.32 ± 0.03^Fb^	6.48 ± 0.00^Ca^	-	-
	3–6	7.50 ± 0.03^ABa^	5.60 ± 0.00^Hb^	-	-	-

A–G: Uppercase letters within the same column indicate comparisons among cheese varieties across storage periods.

a–d: Lowercase letters within the same row indicate comparisons among samples at different storage periods. Different letters indicate statistically significant differences between samples, whereas identical letters indicate no statistically significant difference.

**-**: Because of the cheese samples had melted, analysis did not be performed.

**Table 14:** Total mesophilic aerobic bacteria counts measured during storage in white cheese products containing different stabilizers and salts in their brine (log cfu/g).

	Stabilizer % - Salt %	1st day	15th day	30th day	60th day	90th day
Carrageenan gum	0–2	6.90 ± 0.00^Cc^	8.00 ± 0.00^Aa^	7.58 ± 0.16^Ab^	-	-
	0–4	7.65 ± 0.00^Ab^	6.95 ± 0.00^Ca^	-	-	-
	0–6	6.78 ± 0.00^Cc^	7.95 ± 0.00^Aa^	7.45 ± 0.10^ABb^	-	-
	0.5–2	6.54 ± 0.09^Db^	6.98 ± 0.03^Ca^	6.65 ± 0.07^Db^	6.60 ± 0.01^BCb^	-
	0.5–4	7.48 ± 0.05^ABa^	6.69 ± 0.12^DEb^	6.48 ± 0.00^DEb^	-	-
	0.5–6	5.74 ± 0.06^Fc^	6.48 ± 0.00^Eb^	7.23 ± 0.00^Ba^	-	-
	1–2	6.00 ± 0.06^Ed^	7.34 ± 0.03^Ba^	6.48 ± 0.00^DEc^	7.47 ± 0.00^Aa^	6.95 ± 0.02^Ab^
	1–4	7.41 ± 0.02^Ba^	6.77 ± 0.10^CDb^	6.30 ± 0.00^Ed^	6.40 ± 0.01^Ccd^	6.65 ± 0.00^ABbc^
	1–6	6.48 ± 0.00^Db^	7.00 ± 0.06^Ca^	6.54 ± 0.09^DEb^	-	-
	1.5–2	5.78 ± 0.00^Fc^	6.93 ± 0.04^Ca^	6.30 ± 0.00^Eb^	6.93 ± 0.04^Ba^	6.20 ± 0.01^Cb^
	1.5–4	6.39 ± 0.12^Db^	6.54 ± 0.09^Eab^	6.90 ± 0.00^Ca^	6.50 ± 0.02^Cb^	6.45 ± 0.00^BCb^
	1.5–6	6.16 ± 0.02^Ec^	6.79 ± 0.00^CDa^	6.54 ± 0.01^DEb^	-	-
Xanthan gum	0–2	6.90 ± 0.00^Bc^	8.00 ± 0.00^Aa^	7.58 ± 0.16^Bb^	-	-
	0–4	7.65 ± 0.00^Ab^	6.95 ± 0.00^Ea^	-	-	-
	0–6	6.78 ± 0.00^Bc^	7.95 ± 0.00^Aa^	7.45 ± 0.10^BCb^	-	-
	0.5–2	6.50 ± 0.01^Cc^	7.15 ± 0.00^Da^	6.81 ± 0.05^EFb^	-	-
	0.5–4	7.25 ± 0.02^CDb^	7.24 ± 0.02^CDa^	7.02 ± 0.03^DEa^	-	-
	0.5–6	6.80 ± 0.00^CDEb^	6.39 ± 0.12^Ga^	6.60 ± 0.00^FGa^	-	-
	1–2	6.90 ± 0.03^Cc^	6.85 ± 0.00^Ea^	6.54 ± 0.09^FGb^	-	-
	1–4	7.30 ± 0.02^Bb^	7.39 ± 0.04^BCa^	7.34 ± 0.00^BCa^	-	-
	1–6	6.90 ± 0.01^DEc^	6.95 ± 0.07^Ea^	6.48 ± 0.00^Gb^	-	-
	1.5–2	6.75 ± 0.03^DEc^	6.65 ± 0.07^Fb^	7.00 ± 0.06^DEa^	-	-
	1.5–4	7.10 ± 0.02^EFb^	6.85 ± 0.00^Ea^	7.24 ± 0.05^CDa^	-	-
	1.5–6	7.35 ± 0.03^Fc^	7.47 ± 0.01^Bb^	8.07 ± 0.16^Aa^	-	-
Gelatin	0–2	6.90 ± 0.00^BCc^	8.00 ± 0.00^Aa^	7.58 ± 0.16^Bb^	-	-
	0–4	7.65 ± 0.00^Aa^	6.95 ± 0.00^Ab^	-	-	-
	0–6	6.78 ± 0.00^CDc^	7.95 ± 0.00^Aa^	7.45 ± 0.10^Bb^	-	-
	1–2	6.60 ± 0.00^Dd^	6.99 ± 0.12^Ac^	7.97 ± 0.02^Ab^	8.31 ± 0.07^Aa^	8.41 ± 0.06^Aa^
	1–4	7.68 ± 0.03^Aa^	6.85 ± 0.21^Ab^	8.01 ± 0.01^Aa^	-	-
	1–6	6.99 ± 0.12^Ba^	7.02 ± 0.45^Aa^	7.49 ± 0.02^Ba^	-	-
	2–2	7.56 ± 0.03^Aab^	7.35 ± 0.07^Abc^	7.11 ± 0.10^Cc^	7.69 ± 0.06^Ba^	7.40 ± 0.03^Bab^
	2–4	7.75 ± 0.02^Aa^	7.22 ± 0.20^Ab^	6.93 ± 0.04^Cb^	7.40 ± 0.03^BCab^	7.30 ± 0.01^Bab^
	2–6	6.65 ± 0.07^Da^	7.41 ± 0.47^Aa^	7.41 ± 0.04^Ba^	-	-
	3–2	6.30 ± 0.00^Ea^	7.24 ± 0.76^Aa^	7.55 ± 0.04^Ba^	7.30 ± 0.02^Ca^	7.10 ± 0.01^Ba^
	3–4	6.90 ± 0.08^BCb^	6.89 ± 0.27^Ab^	7.86 ± 0.02^Aa^	7.20 ± 0.04^CDb^	-
	3–6	6.00 ± 0.00^Fb^	7.12 ± 0.01^Aa^	7.10 ± 0.02^Ca^	6.90 ± 0.02^Da^	-
Pectin	0–2	6.90 ± 0.00^DEFc^	8.00 ± 0.00^Aa^	7.58 ± 0.16^Ab^	-	-
	0–4	7.65 ± 0.00^Aa^	6.95 ± 0.00^Bb^	-	-	-
	0–6	6.78 ± 0.00^Fc^	7.95 ± 0.00^Aa^	7.45 ± 0.10^Ab^	-	-
	1–2	6.93 ± 0.04^CDEFa^	6.87 ± 0.04^Ba^	-	-	-
	1–4	7.00 ± 0.06^BCDEa^	5.90 ± 0.00^Ec^	6.65 ± 0.02^Bb^	6.80 ± 0.03^Bab^	-
	1–6	7.10 ± 0.02^Ba^	6.87 ± 0.04^Bb^	6.70 ± 0.00^Bc^	6.85 ± 0.05^Bbc^	-
	2–2	6.85 ± 0.00^EFbc^	6.70 ± 0.00^Cc^	6.93 ± 0.04^Bb^	7.87 ± 0.04^Aa^	-
	2–4	7.08 ± 0.00^BCb^	6.60 ± 0.00^Ca^	-		-
	2–6	6.90 ± 0.00^DEFb^	6.32 ± 0.03^Da^	-	-	-
	3–2	6.87 ± 0.04^EFa^	6.37 ± 0.04^Dc^	6.70 ± 0.00^Bb^	-	-
	3–4	7.04 ± 0.06^BCDa^	6.41 ± 0.02^Db^	6.95 ± 0.04^Ba^	-	-
	3–6	6.54 ± 0.09^Ga^	5.39 ± 0.12^Fb^	-	-	-

A–G: Uppercase letters within the same column indicate comparisons among cheese varieties across storage periods.

a–d: Lowercase letters within the same row indicate comparisons among samples at different storage periods. Different letters indicate statistically significant differences between samples, whereas identical letters indicate no statistically significant difference.

**-**: Because of the cheese samples had melted, analysis did not be performed.

Total yeast-mold analysis was performed on white cheese samples with different stabilizers added to their brines, and the 90-day values for total yeast-mold counts are presented in Table 15. Total yeast-mold counts of the samples showed an increase compared to the beginning of storage. The lowest total yeast-mold count was measured as 1.0 log cfu/g on day 1 of the cheese with 2% pectin and 2% salt in its brine. The highest total yeast-mold count was measured as 6.7 log cfu/g in the day 1 analysis of the cheese with 1% xanthan gum and 4% salt in its brine. Total yeast-mold counts of the samples showed fluctuating changes during storage and did not follow a regular pattern.

**Table 15:** Yeast-mold counts measured during storage in white cheese products containing different stabilizers and salts in their brine (log cfu/g).

	Stabilizer % - Salt %	1st day	15th day	30th day	60th day	90th day
Carrageenan gum	0–2	1.50 ± 0.00^Gc^	2.30 ± 0.00^Fb^	2.60 ± 0.00^Fa^	-	-
	0–4	1.35 ± 0.00^Gb^	2.60 ± 0.00^DEFa^	-	-	-
	0–6	1.78 ± 0.00^EFb^	2.30 ± 0.00^Fa^	2.50 ± 0.01^Fa^	-	-
	0.5–2	2.11 ± 0.10^CDd^	2.70 ± 0.00^DEc^	3.70 ± 0.00^Ca^	3.30 ± 0.01^Ab^	-
	0.5–4	2.19 ± 0.02^BCb^	2.39 ± 0.12^EFb^	3.54 ± 0.09^Da^	-	-
	0.5–6	1.87 ± 0.04^DEc^	2.39 ± 0.12^EFb^	3.60 ± 0.00^CDa^	-	-
	1–2	2.28 ± 0.03^ABCb^	2.83 ± 0.18^CDab^	3.74 ± 0.06^Ca^	3.25 ± 0.02^Aab^	3.00 ± 0.01^Aab^
	1–4	2.43 ± 0.02^ABc^	3.50 ± 0.03^Aab^	3.90 ± 0.00^Ba^	3.00 ± 0.00^Abc^	3.45 ± 0.01^Aab^
	1–6	2.46 ± 0.09^Ab^	3.10 ± 0.02^BCa^	2.98 ± 0.03^Ea^	-	-
	1.5–2	1.48 ± 0.00^Gd^	2.30 ± 0.00^Fc^	4.28 ± 0.03^Aa^	3.01 ± 0.00^Ab^	4.09 ± 0.12^Aa^
	1.5–4	2.29 ± 0.08^ABCc^	2.39 ± 0.12^EFc^	4.41 ± 0.01^Aa^	3.00 ± 0.00^Ab^	3.85 ± 0.01^Aa^
	1.5–6	1.54 ± 0.09^FGc^	3.26 ± 0.12^ABb^	3.70 ± 0.00^Ca^	-	-
Xanthan gum	0–2	1.50 ± 0.00^FGc^	2.30 ± 0.00^FGb^	2.60 ± 0.00^Fa^	-	-
	0–4	1.35 ± 0.00^Gb^	2.60 ± 0.00^EFa^	-	-	-
	0–6	1.78 ± 0.00^Fb^	2.30 ± 0.00^FGa^	2.50 ± 0.01^Fa^	-	-
	0.5–2	2.62 ± 0.08^Ba^	2.84 ± 0.09^DEc^	3.69 ± 0.12^BCDb^	-	-
	0.5–4	2.61 ± 0.16^BCa^	2.65 ± 0.07^EFb^	3.04 ± 0.06^Eb^	-	-
	0.5–6	2.60 ± 0.00^BCDa^	2.89 ± 0.16^CDEc^	3.42 ± 0.12^Db^	-	-
	1–2	2.62 ± 0.04^Ba^	3.69 ± 0.12^ABb^	3.74 ± 0.06^BCb^	-	-
	1–4	2.67 ± 0.02^Aa^	3.18 ± 0.10^BCDc^	4.46 ± 0.04^Ab^	-	-
	1–6	2.58 ± 0.14^CDa^	2.00 ± 0.00^Gc^	3.54 ± 0.09^CDb^	-	-
	1.5–2	2.58 ± 0.05^CDa^	3.78 ± 0.05^Ac^	4.29 ± 0.02^Ab^	-	-
	1.5–4	2.57 ± 0.17^DEa^	3.39 ± 0.06^ABCc^	3.90 ± 0.08^Bb^	-	-
	1.5–6	2.54 ± 0.12^Ea^	3.24 ± 0.34^BCDb^	3.54 ± 0.09^CDb^	-	-
Gelatin	0–2	1.50 ± 0.00^Bc^	2.30 ± 0.00^Db^	2.60 ± 0.00^Da^	-	-
	0–4	1.35 ± 0.00^Bb^	2.60 ± 0.00^Ca^	-	-	-
	0–6	1.78 ± 0.00^Bb^	2.30 ± 0.00^Da^	2.50 ± 0.01^Da^	-	-
	1–2	2.61 ± 0.08^Ab^	3.15 ± 0.02^ABb^	4.42 ± 0.08^Ca^	5.00 ± 0.23^Aa^	5.07 ± 0.23^Ba^
	1–4	2.60 ± 0.16^Ac^	3.10 ± 0.02^ABb^	5.01 ± 0.01^Aa^	-	-
	1–6	2.60 ± 0.00^Ac^	3.16 ± 0.02^ABb^	5.15 ± 0.04^Aa^	-	-
	2–2	2.62 ± 0.04^Ac^	3.11 ± 0.02^ABc^	4.74 ± 0.01^Bb^	5.67 ± 0.23^Aa^	5.60 ± 0.06^Aa^
	2–4	2.66 ± 0.02^Ab^	2.92 ± 0.11^Bb^	5.00 ± 0.00^Aa^	5.40 ± 0.20^Aa^	5.30 ± 0.02^ABa^
	2–6	2.57 ± 0.14^Ac^	3.20 ± 0.02^Ab^	4.59 ± 0.08^BCa^	-	-
	3–2	2.59 ± 0.05^Ac^	3.15 ± 0.04^ABb^	5.02 ± 0.01^Aa^	5.10 ± 0.12^Aa^	5.00 ± 0.03^Ba^
	3–4	2.57 ± 0.17^Ac^	3.16 ± 0.02^ABb^	5.18 ± 0.01^Aa^	5.15 ± 0.03^Aa^	-
	3–6	2.52 ± 0.12^Ac^	3.21 ± 0.02^Ab^	4.69 ± 0.07^Ba^	5.00 ± 0.02^Aa^	-
Pectin	0–2	1.50 ± 0.00^BCDc^	2.30 ± 0.00^Eb^	2.60 ± 0.00^Ca^	-	-
	0–4	1.35 ± 0.00^CDb^	2.60 ± 0.00^CDa^	-	-	-
	0–6	1.78 ± 0.00^Ab^	2.30 ± 0.00^Ea^	2.50 ± 0.01^Ca^	-	-
	1–2	1.66 ± 0.07^ABb^	2.78 ± 0.00^Ba^	-	-	-
	1–4	1.60 ± 0.07^ABCc^	3.34 ± 0.03^Aa^	3.10 ± 0.01^Ab^	3.05 ± 0.02^Bb^	-
	1–6	1.65 ± 0.07^ABd^	3.45 ± 0.03^Ab^	2.84 ± 0.09^Bc^	4.00 ± 0.05^Aa^	-
	2–2	1.00 ± 0.00^Ed^	2.30 ± 0.00^Eb^	2.00 ± 0.00^Dc^	4.04 ± 0.05^Aa^	-
	2–4	1.65 ± 0.07^ABb^	2.60 ± 0.00^CDa^	-	-	-
	2–6	1.30 ± 0.00^Db^	2.30 ± 0.00^Ea^	-	-	-
	3–2	1.24 ± 0.06^DEb^	2.74 ± 0.06^BCa^	2.80 ± 0.09^ABa^	-	-
	3–4	1.00 ± 0.00^Ec^	2.30 ± 0.00^Eb^	2.84 ± 0.09^Ba^	-	-
	3–6	1.30 ± 0.00^Db^	2.48 ± 0.00^Da^	-	-	-

A–G: Uppercase letters within the same column indicate comparisons among cheese varieties across storage periods.

a–d: Lowercase letters within the same row indicate comparisons among samples at different storage periods. Different letters indicate statistically significant differences between samples, whereas identical letters indicate no statistically significant difference.

**-**: Because of the cheese samples had melted, analysis did not be performed.

### 3.5 Multivariate statistical analyses

Pearson correlation analysis, principal component analysis, and two-way clustering heatmaps were performed using XLSTAT software, and the results are presented in Figures 3, 4. The clustering structure created using the Ward coupling method and Euclidean distance was evaluated for both samples and variables.

**Figure 3:**
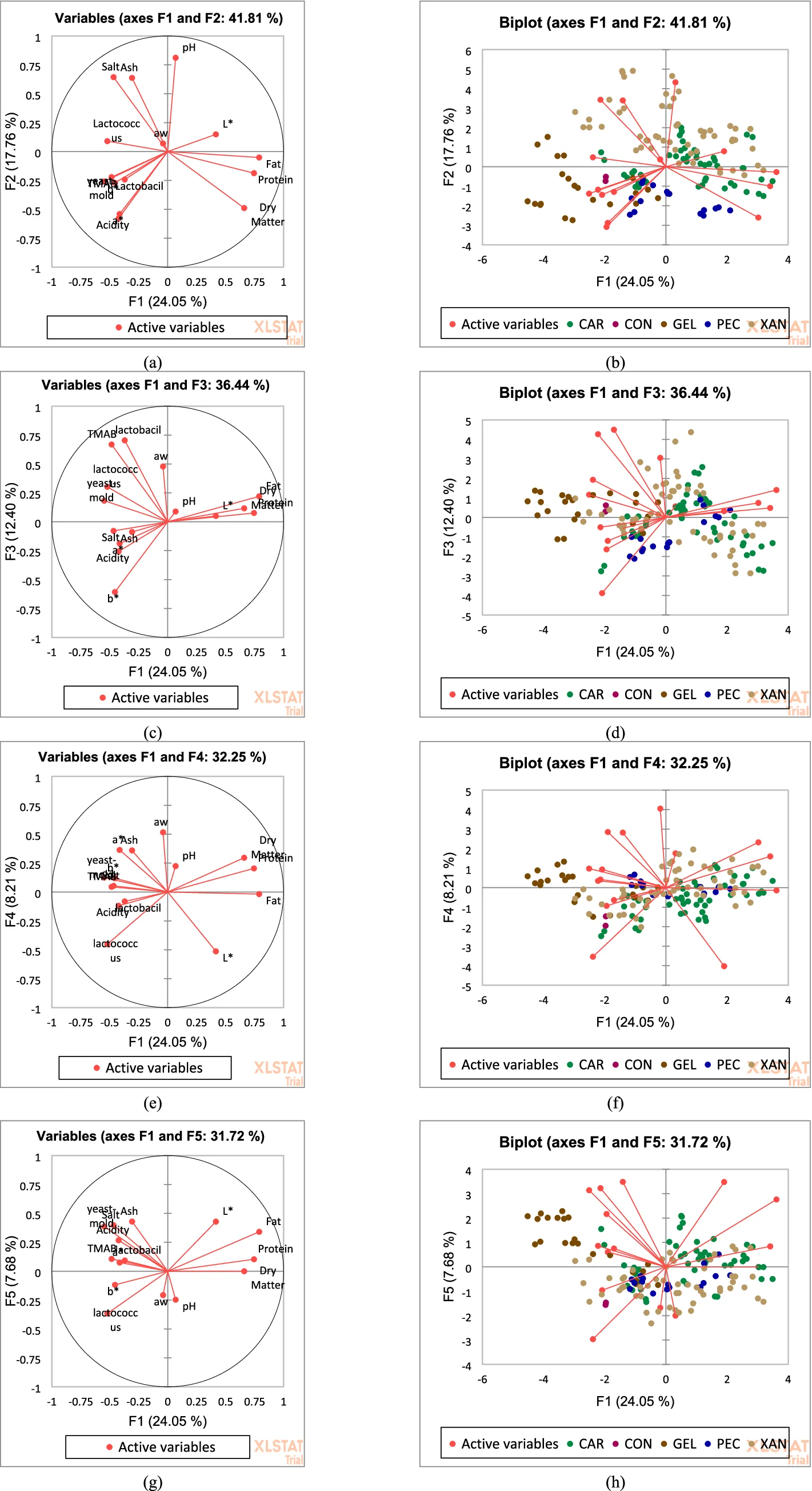
Biplot of the PCs loading and score vectors: **(a)** Variables (axes F1 and F2); **(b)** Biplot (axes F1 and F2); **(c)** Variables (axes F1 and F3); **(d)** Biplot (axes F1 and F3); **(e)** Variables (axes F1 and F4); **(f)** Biplot (axes F1 and F4); **(g)** Variables (axes F1 and F5); **(h)** Biplot (axes F1 and F5).

**Figure 4:**
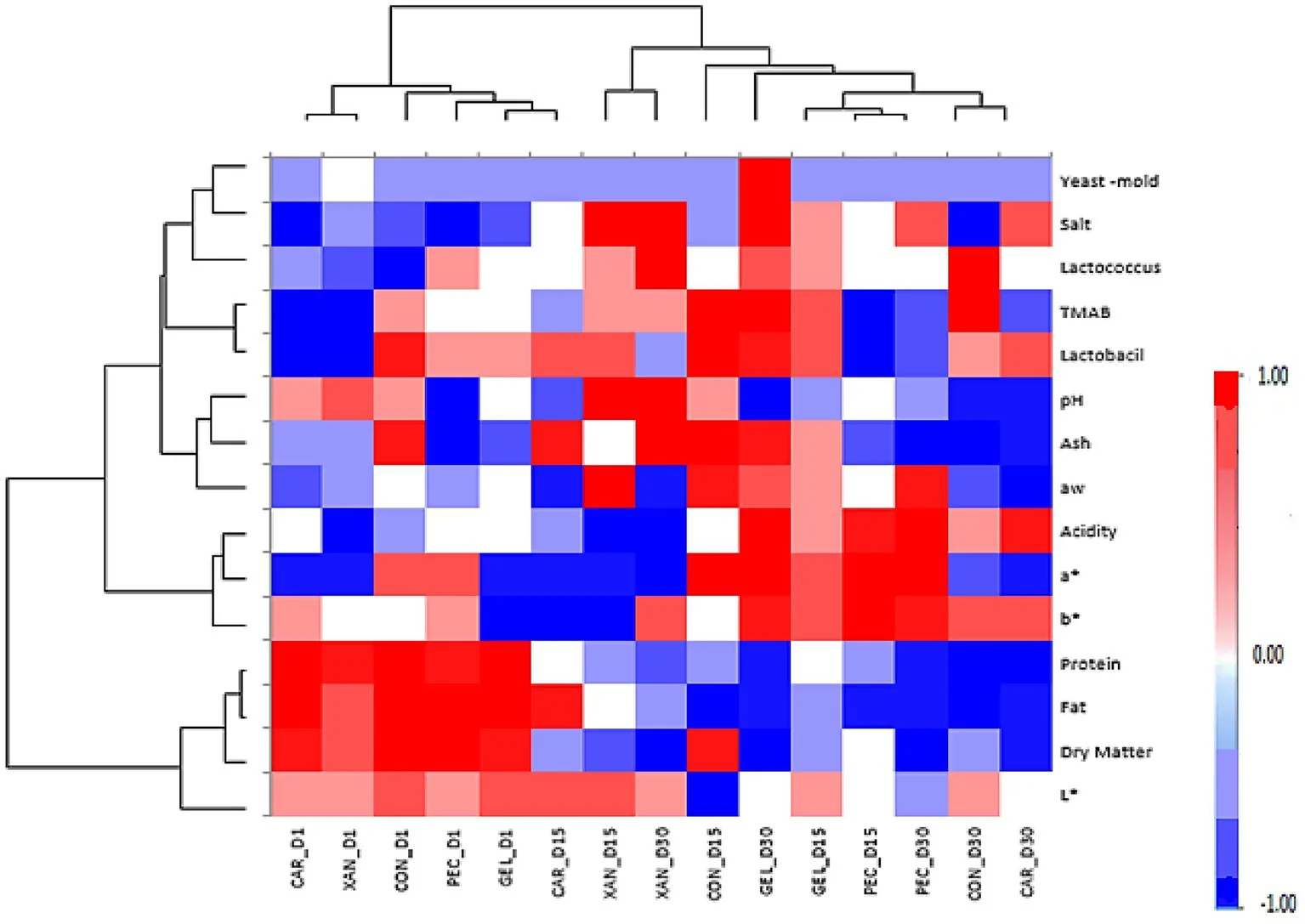
Heat map of physicochemical, color, and microbiological compounds measured during storage in white cheese products containing different stabilizers and salts in their brine.

#### 3.5.1 Correlation results

Table 16 shows the correlation coefficients between 15 variables. There are several important correlation parameters between variables. For example, there are positive and negative correlations between dry matter value and physicochemical values. The dry matter value showed a positive correlation with fat and protein, while showing a negative correlation with salt value. Additionally, pH showed a negative correlation with acidity. In addition, fat showed a positive correlation with protein. These results are to be expected because each parameter should inherently be related to the others. Moreover, ash showed a positive correlation with salt value. *Lactobacil* showed a positive correlation with TMAB.

**Table 16:** Correlations between the parameters.

Variables	Dry Matter	Fat	Ash	aw	L*	a*	b*	Salt	Protein	pH	Acidity	*Lactobacil*	*Lactococcus*	Yeast-Mold	TMAB
Dry Matter	**1**	0.550	−0.391	0.057	0.011	0.027	−0.193	−0.535	0.558	−0.291	−0.103	−0.070	−0.435	−0.176	−0.102
Fat	0.550	**1**	−0.107	−0.034	0.423	−0.301	−0.427	−0.307	0.616	−0.028	−0.259	−0.103	−0.366	−0.273	−0.161
Ash	−0.391	−0.107	**1**	0.033	−0.134	−0.097	0.032	0.660	−0.225	0.402	−0.136	−0.040	−0.057	0.144	0.005
aw	0.057	−0.034	0.033	**1**	−0.074	0.052	−0.172	−0.015	0.062	0.199	−0.132	0.131	0.075	0.135	0.190
L*	0.011	0.423	−0.134	−0.074	**1**	−0.251	−0.259	0.028	0.236	0.004	−0.139	−0.150	−0.083	−0.107	−0.200
a*	0.027	−0.301	−0.097	0.052	−0.251	**1**	0.442	−0.054	−0.140	−0.331	0.422	0.158	−0.041	0.300	0.209
b*	−0.193	−0.427	0.032	−0.172	−0.259	0.442	**1**	0.030	−0.227	−0.201	0.357	−0.193	0.106	0.217	−0.046
Salt	−0.535	−0.307	0.660	−0.015	0.028	−0.054	0.030	**1**	−0.401	0.358	−0.046	0.000	0.130	0.139	0.084
Protein	0.558	0.616	−0.225	0.062	0.236	−0.140	−0.227	−0.401	**1**	−0.025	−0.199	−0.215	−0.362	−0.221	−0.209
pH	−0.291	−0.028	0.402	0.199	0.004	−0.331	−0.201	0.358	−0.025	**1**	−0.558	−0.204	0.050	−0.269	−0.095
Acidity	−0.103	−0.259	−0.136	−0.132	−0.139	0.422	0.357	−0.046	−0.199	−0.558	**1**	0.112	0.046	0.309	0.171
Lactobacil	−0.070	−0.103	−0.040	0.131	−0.150	0.158	−0.193	0.000	−0.215	−0.204	0.112	**1**	0.268	0.245	0.640
Lactococcus	−0.435	−0.366	−0.057	0.075	−0.083	−0.041	0.106	0.130	−0.362	0.050	0.046	0.268	**1**	0.219	0.341
Yeast-mold	−0.176	−0.273	0.144	0.135	−0.107	0.300	0.217	0.139	−0.221	−0.269	0.309	0.245	0.219	**1**	0.405
TMAB	−0.102	−0.161	0.005	0.190	−0.200	0.209	−0.046	0.084	−0.209	−0.095	0.171	0.640	0.341	0.405	**1**

Color scale ranges from −1 (blue) to +1 (red), representing correlations. *denotes the L, a, and b* parameters.

#### 3.5.2 PCA results

In the literature, PCA and heat map analysis have been successfully used to correlate the physicochemical, color, and microbiological properties of white brined cheese in Tables 17–19 and Figure 3. In this study, five PCs (principal components) were identified as significant in explaining the total variability of the product properties. Table 17 shows the eigenvalues and variances of each PC. As can be seen, the eigenvalues of PC1-PC4 were found to be greater than 1. PC1, PC2, PC3, PC4 and PC5 explained 24.04, 17.76, 12.40, 8.20 and 7.67% of the total variability in the dataset, respectively. In other words, 70.08% of the total variance in the 15 variables tested can be reduced to four new variables (PCs). This percentage can be considered sufficient to explain the variability, as it is higher than 70%. The PC coefficients (loadings) and contributions of the significant principal components are shown in Table 18. The results of the PCA analysis, which was performed using data on the physicochemical, color, and microbial properties of white brined cheese, are given in Table 19.

**Table 17:** Results of PCA analysis using data of all properties of the white cheese products containing different stabilizers and salts in their brine.

	F1	F2	F3	F4	F5	F6	F7	F8	F9	F10	F11	F12	F13	F14	F15
Eigenvalue	3,607	2,664	1,860	1,231	1,151	0,904	0,667	0,607	0,495	0,392	0,362	0,357	0,313	0,212	0,179
Variability (%)	24,045	17,762	12,400	8,206	7,675	6,029	4,445	4,044	3,302	2,611	2,416	2,378	2,084	1,415	1,190
Cumulative %	24,045	41,807	54,206	62,412	70,087	76,116	80,561	84,605	87,907	90,518	92,934	95,311	97,395	98,810	100,000

**Table 18:** Principal component coefficients (loadings) and their contributions.

	1st loading		2nd loading		3nd loading		4th loading		5th loading	
	Contributions		Contributions		Contributions		Contributions		Contributions	
Components	PC1	(%)	PC2	(%)	PC3	(%)	PC4	(%)	PC5	(%)
Chemical composition										
Dry matter	**0.660**	12.077	−0.489	8.970	0.116	0.728	0.296	7.130	0.000	0.000
Fat	**0.789**	17.278	−0.051	0.099	0.220	2.606	−0.018	0.026	0.341	10.080
Protein	**0.745**	15.369	−0.187	1.310	0.076	0.309	0.204	3.392	0.103	0.928
Ash	−0.307	2.621	**0.637**	15.235	−0.088	0.415	0.360	10.510	0.430	16.025
Aw	−0.040	0.045	0.070	0.183	0.478	12.310	**0.516**	21.672	−0.207	3.720
Salt	−0.467	6.045	**0.643**	15.539	−0.079	0.334	0.053	0.229	0.398	13.728
Ph	0.068	0.130	**0.811**	24.705	0.089	0.424	0.224	4.089	−0.247	5.290
Acidity	−0.423	4.972	**−0.579**	12.566	−0.257	3.544	−0.119	1.148	0.267	6.193
Color values										
L*	0.416	4.792	0.149	0.829	0.050	0.137	**−0.513**	21.397	0.429	15.973
a*	−0.415	4.768	**−0.540**	10.959	−0.188	1.907	0.364	10.743	0.075	0.484
b*	−0.455	5.745	−0.270	2.743	**−0.608**	19.867	0.119	1.151	−0.118	1.217
Microbiological parameters										
Lactobacil	−0.371	3.809	−0.242	2.201	**0.705**	26.688	−0.081	0.535	0.093	0.745
Lactococcus	**−0.521**	7.520	0.090	0.304	0.302	4.912	−0.451	16.554	−0.366	11.615
Yeast-mold	**−0.548**	8.325	−0.259	2.520	0.182	1.787	0.125	1.262	0.387	13.023
TMAB	−0.484	6.504	−0.221	1.836	**0.669**	24.033	0.045	0.162	0.106	0.978

For each parameter, the factor loadings across PC1–PC4 were compared, and the loading with the highest absolute value was selected.

**Table 19:** Variables belonging to the principial components.

PC1:	*Lactococcus*, Yeast-mold, Protein, fat, dry matter
PC2:	a* value, Acidity, pH, Salt, Ash
PC3:	*Lactobacil*, TMAB, b* value,
PC4:	L* value, a_w_
PC5:	-

The table below shows which basic components the variables belong to. Score graphs and PC loadings are shown in Figure 3; F1-F2, F1-F3 and F1-F4 graphs are also presented here. The F1-F2 score graph shows that gelatin brine is positively correlated with ash, aw, *Lactococcus* and salt content, as these are located in the same quartile. The clustering in the upper right quartile of the F1-F3 graph is due to correlations with carregenan brine, pH, dry matter, fat, protein, L value. The relationships observed from these score graphs have been explained in detail previously using correlation analysis (Table 16).

#### 3.5.3 HCA results

In cheese samples containing carrageenan in their brine, on the first day, dry matter, protein and fat values were high (red zone), while a* values, *Lactobacil*, TMAB and salt values were low (blue zone). On the 15th day, ash and fat values were high (red zone), while aw, a* and * values were low (blue zone). On the 30th day, acidity values were high (red zone), while protein, fat, dry matter, ash, pH and aw were low (blue zone).

In cheese samples containing xantan in their brine, on the first day, protein values were high (red zone), while *Lactobacil*, TMAB, acidity, a* values were low (blue zone). On the 15th day, aw, pH and salt were high (red zone); while acidity, a* and b* values were low (blue zone). On the 30th day, pH, salt, ash and *Lactococcus* values were high (red zone), while acidity, dry matter, aw, a* value were low (blue zone).

In cheese samples containing pectin in their brine, on the first day, dry matter, protein and fat values were high (red zone); while a* values, ash, pH and salt values were low (blue zone). On the 15th day, acidity, a* and b* values were high (red zone); while fat, TMAB, *Lactobacil* and ash values were low (blue zone). On the 30th day, acidity, aw, a* and b* values were high (red zone); while protein, fat, dry matter, and ash were low (blue zone).

In cheese samples containing gelatin in their brine, on the first day dry matter, protein and fat values were high (red zone), while a* and b* values were low (blue zone). On the 30th day acidity, yeast-mold, salt, a* and b* values were high (red zone); while protein, fat, dry matter and pH were low (blue zone).

In control cheese samples, on the first day ash, *Lactobacil*, dry matter, protein and fat values were high (red zone), while *Lactococcus* values were low (blue zone). On the 15th day, dry matter, aw, a* value, ash, *Lactobacil* and TMAB were high (red zone), while fat and L* values were low (blue zone). On the 30th day, TMAB, *Lactococcus* were high (red zone); while salt, pH, ash, protein and fat values were low (blue zone).

## 4 Discussion

Stabilizers, also called hydrocolloids, are of plant and animal origin. However, they perform many functions in food, some of which include gelling, thickening, and stabilizing the product. The working principle of stabilizers is that they either bind the free water in food as hydration water or react with the proteins in the food structure to increase the hydration degree (27). At the same time, stabilizers, together with milk components, prevent the movement of free water in the food, thus enabling the formation of a gel structure in the food. Another advantage of stabilizers in dairy products is that they strengthen the gel structure and consequently increase the resistance to syneresis (28–31).

Gelatin, which is a high-molecular-weight protein, is obtained by treating various animal collagens such as cow, pig, and fish with acid or alkali (32). It is used very intensively in the food sector in our country. Gelatin is used for various purposes in many dairy products, especially yogurt (33). Gelatin also plays an active role in the food industry as a thickener in emulsion products and in the formation of gel network structure (34). Gelatins are divided into three groups according to their degree of bloom: high, medium and low gel properties. High-bloom gelatin is widely used in milk and dairy products, especially in fruit yogurts (35).

Carrageenan gums perform various functions in dairy products, such as increasing viscosity, accelerating protein function, tightening fat structure, and providing stable structure to dairy products (36). Not all carrageenan gums perform the same function; for example, κ- and ι-carrageenan gums form gels in milk and dairy products, while λ-carrageenan gum plays an active role in thickening foods (32, 34, 37). In addition to forming high-viscosity gels (34), carrageenan gums adapt to the movements of proteins when they are in the same environment as proteins (28, 37).

Xanthan gum is effectively used in the food industry, especially in ice cream, because of its high water-holding capacity, its role in obtaining products with good viscosity, and its good thickening properties (38). Xanthan gum has a different structure in aqueous solutions depending on the temperature. Due to its helical structure at high temperatures, xanthan gum has an effective gel structure (34). At the same time, xanthan gum molecules dissolve very quickly in hot and cold water regardless of the temperature. Xanthan gum has an effective viscosity even at very low concentrations (28, 39, 40). Since xanthan gum, like carrageenan gum, has synergistic properties, its gelling and thickening properties become more pronounced when used together with other polysaccharides.

The variability of dry matter values in brined cheeses throughout storage has been demonstrated in numerous studies. Nespolo and Brandelli (41) reported dry matter values of 44.3–37.6% in Feta cheeses made from sheep and goat milk, while Pitso and Bester (42) reported 39.98–41.28% in Feta cheese made from a mixture of cow and goat milk. Prinsloo (43) found a moisture content of 40–63.5% in Feta cheeses made from cow milk; Pexara et al. (44) reported 53.4% in Feta cheese; Vassiliadis et al. (45) reported a moisture content of 60.99 and 62% in Feta cheeses they produced from raw and heat-treated sheep milk. Other researchers similarly reported that dry matter percentage values in white cheeses decreased at the end of storage (2, 16, 46). They attributed this decrease to the increased heat treatment temperature applied to milk (47). While TS 591 requires a minimum dry matter content of 40% in white cheese, according to the draft Turkish Food Codex Cheese Communiqué, this ratio may be set at 35% depending on the cheese type (48). The results we obtained in our study are consistent with this communiqué. The cause of this increase in dry matter was attributed to the fact that as acidity in cheese increased, the water-holding capacity of casein decreased, and as a result, the cheese lost water and its dry matter increased. Our findings showed parallelism with some studies (47) and also complied with the Turkish Food Codex Cheese Communiqué.

However, in order for the cheeses produced in our study to resemble those produced in industry, pasteurization was performed at high temperature. Cankurt (16) reported that the fat ratios of cheeses obtained from milk pasteurized at high temperatures were relatively lower. While the sample with the highest fat content at the beginning of storage was the cheese containing carrageenan gum in its brine, the sample with the lowest value at the end of storage was the cheese containing pectin in its brine. The decrease in fat ratios during storage in white cheese samples with different stabilizers and salt levels in their brines was not due to fat transfer to brine, but rather to the proportional decrease in fat resulting from the absorption of water by the cheeses. Nespolo and Brandelli (41) reported fat content of 34.2 and 37.3% in Feta cheeses made from sheep and goat milk; Pitso and Bester (42) reported fat content of 17–19% in Feta cheeses made from cow and goat milk mixture; Pexara et al. (44) reported a fat content of 26.1% in Feta cheeses. Sancak and Sancak (49) and Karaca (50) indicated that because some of the water-soluble proteins also occur with fats, when transferred to water they carry small amounts of fat with them, leading to a decrease in fat values at the end of storage. In accordance with this, the lower-than-expected fat values in the cheeses were due to the high-temperature pasteurization of the milk; after the pasteurization process, whey that should have been removed remained in the cheese in greater quantities than it should have, and because serum proteins and other components found in curd cheese have high water-holding capacity, the cheese retains more water than necessary, leading to a proportionally lower fat content.

Kırkın (51) reported that ash ratios changed statistically significantly in fresh and ripened white cheeses. Koyuncu (46) found ash content in cheeses to be 3.75% at the beginning of storage and 5.43%) at the end of storage. Researchers in the literature have found variable amounts of ash in cheese. Salt in the cheese structure directly affects ash content (52). Since the salt levels used in the brines with stabilizers were different, it is normal for the ash content of the cheeses to differ. The increase in ash content of the cheeses was mainly associated with changes in salt content, as sodium chloride constitutes a major fraction of the mineral components measured as ash. The low salt concentration of the brine promoted water migration into the cheese matrix, leading to increased moisture content, softening of the cheese structure, and a decrease in dry matter values. Simultaneously, salt diffusion from the brine into the cheese occurred due to the concentration gradient, contributing to the increase in ash content. Therefore, the changes in ash content were related to both salt uptake and moisture variations during storage.

Pitso and Bester (42) reported total protein as 13.77–15.30% in Feta cheeses made from a mixture of cow and goat milk; Prinsloo (43) reported total protein of 12.0–20.8% in Feta cheeses made from cow milk. Cankurt (16) reported that in his study, the protein value in white cheese samples with hydrosol in their brine was 14.41% at the beginning of storage and 8.55% at the end of maturation. The decrease in protein content during storage in brined white cheeses has been explained by the breakdown of proteins into smaller water-soluble compounds and their transfer into the brine (10, 53–55)Salt used in the production and storage of cheese affects biochemical events during ripening, enzymatic and microbial activities, and the composition of cheese, as well as its flavor, rheological, and textural properties (56, 57).

When reviewing publications by other researchers regarding salt values in white cheeses (58) reported 4.21–4.83% and 5.03–5.26%; Karaca (50) reported 1.79–2.53%; and Cankurt (16) reported 3.21–3.86% and 4.51–4.62%. Pitso and Bester (42) reported salt content of 4.00–4.11% in Feta cheeses made from cow and goat milk mixture; Prinsloo (43) reported salt content of 1.58–6.58% in Feta cheeses made from cow milk; Pexara et al. (44) detected 2.20% in Feta cheeses. Koyuncu (46) and Cankurt (2) reported that the salt content of cheeses increased at the end of storage in their studies. The results of our study showed similarity with other studies in the literature and demonstrated an increase at the end of storage. When all these studies are reviewed, it becomes evident how variable salt content in cheese is. The salt content in cheese does not remain as initially added. Many factors affect salt transfer, such as whether the cheese contains starter culture, the heating temperature of the milk used in production, whether calcium chloride was used in production, brine pH, brine temperature, initial salting method of the cheese, and the duration of stay in brine.

There are many studies in the literature tracking pH values on cheese. Hayaloğlu et al. (6) reported pH values between 5.19 and 6.04. Nespolo and Brandelli (41) reported pH values of 5.13–5.89 in Feta cheeses made from sheep and goat milk; Pitso and Bester (42) reported pH values of 4.58–4.63 in Feta cheeses made from cow and goat milk mixture, Prinsloo (43) reported pH values of 4.10–5.30 in Feta cheeses made from cow milk; Anifantakis (59) reported a pH value of 4.41 in Feta cheeses obtained from the market in Greece; Vassiliadis et al. (45) reported a pH value of 5.06 in both groups of Feta cheeses produced from raw and heat-treated sheep milk.

Cankurt (16) attributed the increase in pH of cheeses to the fact that some basic-fragmentation products resulting from proteolysis act as a buffer in acidic environments, raising the pH value. The pH values in our study showed similarity with other studies in the literature and decreased at the end of storage.

Researchers have found total acidity values between 0.82 and 1.31% in fresh white cheeses (60, 61). Ertürkmen (62) and Biçer (63) reported that titratable acidity rose from 1.64 to 2.43% during storage. Cankurt (16) stated that although titratable acidity showed variability during maturation, it increased at the end of storage. Koyuncu (46) also reported that total acidity increased at the end of storage in white cheese. The total acidity values in our study were similar to those of other researchers, and the total acidity of our cheese samples increased at the end of storage as reported by many.

When the values were examined in detail, no significant relationship could be established between the salt content in brine, the type and level of stabilizer in brine, and water activity. This is considered to be due to the fact that dry matter content continuously changes with storage. aw values in different studies on white cheese have been reported to fluctuate, typically ranging from 0.87 to 0.98. These variations are driven by factors such as salt and moisture transfer, glycolysis, and dehydration, which continuously alter the water activity during ripening and storage (64).

When studies in the literature on cheese color values are examined, İlhan et al. (65) in their study examining different types of cheese reported that a* values increased while L* and b* values decreased. They also noted that color is an important parameter for consumers. Cankurt (16) reported that L* and b* values increased in cheeses he produced, while a* value decreased. Gün et al. (66) in their study investigating the effect of modified atmosphere packaging on some attributes of different types of cheese reported that a* values increased while L* and b* values decreased. Khosrowshahi et al. (67) reported L values of 90.82–94.65 and b* values of 11.80–12.94 in Iranian White cheese produced using different concentrations of starter culture. The results of our study show parallelism with other studies. There are many processes that affect cheese color, including heat treatment, the amount of calcium chloride added, and whether the curd is scalded. Since high-temperature heat treatment was applied to the milk when producing cheeses in our study, the color was whiter. If the cheese had been made from low-temperature pasteurized milk, or if curd scalded cheeses had been produced, yellower cheese would have been obtained.

*Lactococcus* constitute the most important group of lactic acid bacteria for general use, and are employed as starter cultures in many fermented dairy products, including cheese (17, 24). The increases that occurred during storage were statistically significant (*p* < 0.05). Significant differences (*p* < 0.05) were also found between samples in comparisons across storage days. From previous studies on white cheese, Şengül et al. (68) found lactococcus counts of 2.3 × 10^7^ cfu/g in cheeses produced from pasteurized milk and 4.1 × 10^6^ cfu/g in cheeses produced from raw milk. Kesenkaş et al. (69). In their study examining village cheeses produced in three different establishments in the İzmir region reported first day lactococcus counts of 6.26–7.74 log. Cankurt (16) reported that the lactococcus counts measured in the cheeses he produced generally increased until day 15 and then decreased again, and that the increases occurring during storage were statistically significant (*p* < 0.05).

The highest value at the beginning of storage was measured as 7.56 log in the sample containing pectin in its brine, while at the end of storage the highest value was 6.30 log in the sample also containing pectin in its brine. The *Lactobacillus* counts of the samples showed fluctuating changes during storage, with no clear pattern. The increases occurring during storage were statistically significant (*p* < 0.05). The differences between samples in comparisons across storage days were also statistically significant (*p* < 0.05). Variables such as cheese packaging form, storage temperatures, hygiene sensitivity during cheese production, and protective starter cultures used in production are some of the factors that affect yeast and mold development. While the highest value at the beginning of storage was 2.74 log cfu/g in the sample containing xanthan in its brine, the highest value at the end of storage was 5.60 log cfu/g in the sample containing gelatin in its brine. In studies in the literature, total yeast-mold counts in white cheeses were reported as 9.5 × 10^3^ on day 1 and 2.8 × 10^2^ cfu/g on day 60 by (70), and mold counts between a minimum of 1.5 × 10^4^ and a maximum of 4.4 × 10^6^ cfu/g by Akyüz and Şimşek (88). Gün et al. (66) reported that mold development was significantly slowed in cheeses stored by modified atmosphere packaging. Cankurt (16) reported that total yeast-mold values were <10^2^ cfu/g in all samples in his study and that yeast-mold could not be detected in the samples. When the results in our study were compared with other studies, it was understood that total yeast-mold content was higher than some and lower than others. Some were found to be similar (71). Kırkın (51) stored white cheeses in packages with different gas combinations (0% O₂ + 75% CO₂ and 10% O₂ + 75% CO₂) and reported that these gas combinations gave good results in preventing yeast-mold. Urhan (72) reported a TMAB count of 5.8 × 10^6^ cfu/g, yeast-mold count of 1.2 × 10^6^ cfu/g, and coliform bacteria count of 5.4 × 10^2^ cfu/g in white cheeses; the same study also detected the presence of pathogenic bacteria: *E. coli*, *S. aureus*, and *L. monocytogenes* in cheeses. Tavsanli et al. (73) in their study adding six different organic acids to cheese brine, reported that fumaric, lactic, and malic acid limited the counts of *Lactobacillus* and *Lactococcus*, while malic and tartaric acid suppressed the growth of *L. monocytogenes*. Özcan et al. (74), reported that in cheeses with different added starter cultures, TMAB count ranged from 5.16 to 9.83 log cfu/g and LAB count (on MRS agar) from 5.85 to 8.78 log cfu/g. Yanmaz (75) reported a TMAB count of 5.59–6.44 log cfu/g in white cheeses held in brines containing 12, 14, and 16% salt concentrations.

Manolopoulou et al. (76), reported yeast-mold count of 3.28–3.94 log cfu/g and LAB count of 7.78–8.32 log cfu/g in traditional Feta cheese. Vassiliadis et al. (45) reported total mesophilic aerobic bacteria count of 8.37 and 7.95 log cfu/g, LAB count (on MRS agar) of 8.29 and 7.90 log cfu/g, and yeast-mold count of 4.77 and 5.05 log cfu/g in Feta cheese. Altın and Tekinşen (77) reported TMAB count of 1.60 × 10^8^ cfu/g, LAB count of 2.68 × 10^7^ cfu/g, and yeast-mold count of 2.46 × 10^5^ cfu/g in brined white cheese. Ceylan and Demirkaya (78) reported total aerobic mesophilic bacteria count of 3.35–6.14 log cfu/g, LAB count of 3.38–6.09 log cfu/g, and yeast-mold count of 1.85–4.67 log cfu/g. Göncüoğlu et al. (79) reported TMAB count of 6.84 log cfu/g in white cheese, Kurşun et al. (80) reported TMAB count of 10^6^–10^9^ cfu/g and yeast-mold count of ≥10^4^ cfu/g in white cheese. Uğur (81) reported TMAB count of 1.0 × 10^8^ cfu/g, and yeast-mold count of 1.0 × 10^6^ cfu/g in homemade white cheese in Muğla. Cankurt (2) noted that there was an inverse relationship between the increase in gum content in brine and bacterial count in cheeses held in stabilized brine. Furthermore, it was determined that generally the highest values were detected in control samples and that, although not very significant, gelatin-containing samples contained a lower number of lactic acid bacteria compared to others.

Principal component analysis revealed a strong positive correlation between moisture content and ash content, suggesting that the water-binding capacity of oat flour increases its moisture-holding capacity (82). For example, a positive correlation has been reported between dry matter content and cohesion value (83), while a negative correlation was reported by Delgado et al. (84). Furthermore, a negative correlation has been reported between fat content and cohesion value Bryant et al. (85). Accordingly, Upreti et al. (86) reported that fat type, degree of proteolysis during ripening, moisture content, and pH value are other important factors that may affect cheese samples. It has also been reported that the level of proteolysis varies depending on the type of cheese; therefore, each type of cheese has a unique proteolysis pattern, which leads to the classification of cheeses using proteolytic data (82).

All cheeses produced from cow’s milk were located in the positive F2 region, where F2 indicates variation in milk type. It was determined that storage time had a significant effect on cheese characteristics; milk type also influenced these characteristics, but to a lesser extent than storage time (87). The conductivity and TDS of brine are positively correlated with the NaCl concentration. It can also be noticed that the pH and b* coordinate of the brine samples’ color are positively correlated with the day of storage, while the a* coordinate of brine samples’ color is positively correlated with the calcium lactate concentration (14).

By preventing the water in the brine from migrating into the cheese, the cheese texture can be preserved for a longer time despite being less salty. In addition, defects such as melting and softening do not occur in the cheese, and the brine water becomes clear. In a previously conducted project, the gel brine technique was tested at 8 and 12% salt concentrations, and the results showed that the gel brine technique was extremely successful. The samples also maintained their top-quality characteristics until the end of storage. The usage rates of the stabilizers used in the research were determined through preliminary trials, since exceeding the appropriate limits leads to various problems. If the stabilizer is used above the selected rate, the brine becomes too thick, making production and application to the product very difficult, whereas if it is below the lower limit, it is ineffective, and the cheeses melt within a day or two. If production is to be done using the gel brine technique, it is recommended not to exceed the rates selected in our study. The stabilizers used in the study did not have a negative effect on taste and smell and could be used without any problems; however, according to our experience from previous studies, guar gum, another stabilizer, is not suitable for use in gel brine, both structurally and in terms of taste and aroma. Even with a 6% salt concentration in brine cheese, it has been observed that the structure of the cheeses is preserved for a long time. Therefore, it is believed that the gel brine technique is successful and can be easily used in white cheese production to avoid excessive salt, which is detrimental to human health, and for this reason, it is thought that reducing the salt content in brine to below 12% in the dairy industry would be beneficial both in terms of health and cost.

## 5 Conclusion

In this study, the gel brine technique enabled white cheese to be stored in brine containing substantially lower salt levels than the traditional 12–14% brine while preserving product quality throughout 90 days of storage at +4 °C. All control cheeses stored in stabilizer-free brine melted completely after the 30th day of storage, so that no measurements could be taken at the later stages of storage (days 60 and 90). In contrast, cheeses kept in gel brine containing gelatin or carrageenan gum retained their texture and flavor until the end of the storage period. In particular, the cheese stored in brine with 3% gelatin and 2% salt, followed by the cheese with 1% carrageenan gum and 2% salt, survived storage without melting and was generally appreciated.

Based on these results, gelatin is recommended as the most suitable stabilizer for the gel brine technique, and storage of white cheese in gel brine containing 3% gelatin and 2% salt is suggested as the optimal treatment for producing low-salt white cheese. Since the method is simple and does not require additional investment, it can be readily applied in the dairy industry and offers a practical contribution to reducing sodium intake while preserving the texture, flavor, and quality of white cheese.

## Data Availability

The original contributions presented in the study are included in the article/Supplementary material, further inquiries can be directed to the corresponding author/s.

## References

[1] Çavuş M. Peynir tuzlamada yeni bir yöntem: Jel salamura tekniği (Doctoral dissertation). Kayseri, Turkey: Fen Bilimleri Enstitüsü (2020).

[2] Cankurt H. The effects of adding different stabilizers in brine on the physicochemical, sensory, microbiological and textural properties of white cheese. Foods. (2019) 8:133. doi: 10.3390/foods8040133, 31010198 PMC6517965

[3] Özbek Ç Güzeler N. Implications of stabilizers used in brine of soft white cheese on the aroma profile of cheeses. Gida. (2023) 48:1199–215. doi: 10.15237/gida.gd22050

[4] Güneş G Çakır ÇA. Impact of gel brine on proteolytic, microbiological, textural properties of raw milk cheese. Dairy. (2025) 7:4–4. doi: 10.3390/dairy7010004

[5] Abd El-Salam MHA Alichanidis E. "Cheese varieties ripened in brine". In: Fox PF McSweeney PLH Cogan TM Guinee TP, editors. Cheese: Chemistry London, UK: Elsevier Academic Press (2004) 2:227–49. doi: 10.1016/S1874-558X(04)80046-0

[6] Hayaloglu AA Guven M Fox PF McSweeney PLH. Influence of starters on chemical, biochemical and sensory changes in Turkish white-brined cheese during ripening. J Dairy Sci. (2005) 88:3460–74. doi: 10.3168/jds.S0022-0302(05)73030-7, 16162519

[7] Hayaloğlu AA Güven M Fox PF. Microbiological, biochemical and technological properties of Turkish white cheese ‘Beyaz Peynir.’. Int Dairy J. (2002) 12:635–48. doi: 10.1016/s0958-6946(02)00055-9

[8] Jia R Mao Y Wei L Pu M Zhao A Chen X . Effects of reduced-sodium brine on the physicochemical, textural and flavor characteristics of sheep cheeses during maturation. Food Sci Anim Prod. (2023) 1:9240039. doi: 10.26599/fsap.2023.9240039

[9] Ferroukhi I Bord C Lavigne R Chassard C Mardon J. Exploring alternative salting methods to reduce sodium content in blue-veined cheeses. SPIRE - Sciences Po Institutional Repository. (2022) 138:105555. doi: 10.1016/j.idairyj.2022.105555

[10] Guinee TP. Salting and the role of salt in cheese. Int J Dairy Technol. (2004) 57:99–109. doi: 10.1111/j.1471-0307.2004.00145.x

[11] Pappas CP Kondyli E Voutsinas LP Mallatou H. Effects of salting method and storage time on composition and quality of feta cheese. Int J Dairy Technol. (1996) 49:113–8. doi: 10.1111/j.1471-0307.1996.tb02502.x

[12] Lazárková Z Šopík T Talár J Purevdorj K Salek RN Buňková L . Quality evaluation of white brined cheese stored in cans as affected by the storage temperature and time. Int Dairy J. (2021) 121:105105. doi: 10.1016/j.idairyj.2021.105105

[13] WHO. WHO Global Report on sodium Intake Reduction. Geneva, Switzerland: World Health Organization (2023).

[14] Jakopović KL Barukčić I Radovčić NM Božanić R Tušek AJ. Reduced sodium in white brined cheese production: artificial neural network modeling for the prediction of specific properties of brine and cheese during storage. Fermentation. (2023) 9:783. doi: 10.3390/fermentation9090783

[15] Anonymous. Türk Gıda Kodeksi Peynir Tebliği (Tebliğ No: 2015/6). Ankara, Türkiye: Resmî Gazete (2015).

[16] Cankurt H. Effect of some Hydrosols and Essential Oils on Various Properties of block type Processed cheese and white cheese. Kayseri, Türkiye: Erciyes Üniversitesi Fen Bilimleri Enstitüsü (2015).

[17] Metin M. Süt ve Mamülleri Analiz Yöntemleri. İzmir, Türkiye: Ege Üniversitesi, Ege Meslek Yüksekokulu Yayınları (2010) 94.

[18] Üçüncü M. A'dan Z'ye Peynir Teknolojisi Cilt II. Izmir: Meta Basım Matbaacılık Hizmetleri (2008).

[19] Kurt A Çakmakçı S Çağlar A. Süt ve Mamülleri Analiz Metotları Rehberi. Erzurum, Türkiye: Atatürk Üniversitesi Yayınları (1996).

[20] Bütikofer U Rüegg M Ardö Y. Determination of nitrogen fractions in cheese: evaluation of a collaborative study. LWT. (1993) 26:271–5. doi: 10.1006/fstl.1993.1056

[21] Kaban G Kaya M. Identification of lactic acid bacteria and gram-positive catalase-positive cocci isolated from naturally fermented sausage (sucuk). J Food Sci. (2008) 73:M385–M388. doi: 10.1111/j.1750-3841.2008.00906.x 19019118

[22] Mitsumoto M O’Grady MN Kerry JP Buckley DJ. Addition of tea catechins and vitamin C on sensory evaluation, colour and lipid stability during chilled storage in cooked or raw beef and chicken patties. Meat Sci. (2005) 69:773–9. doi: 10.1016/j.meatsci.2004.11.010, 22063156

[23] Todaro M Garofalo G Busetta G Gannuscio R Rosa ARD Scatassa ML . Reduction of PDO pecorino Siciliano cheese making duration: microbial dynamics and quality attributes deriving from replacing whey permeate with hot water during cooking. Int J Food Microbiol. (2023) 410:110481. doi: 10.1016/j.ijfoodmicro.2023.110481, 37977081

[24] Halkman K. (2019). Merck Gıda Mikrobiyolojisi Uygulamaları. Ankara, Türkiye. Available online at: http://www.mikrobiyoloji.org

[25] Bintsis Τ Mantzouridou FT Lalou S Alvanoudi P Ordoudi SA Angelidis AS . Comparative analysis of chemical, microbiological, sensory and volatile compound profiles in Manouri PDO and artisanal Manouri cheeses: a preliminary study. Food Bioprocess Technol. (2024) 17:3561–75. doi: 10.1007/s11947-024-03333-z

[26] Abdi H Williams LJ. "Tukey’s honestly significant difference (HSD) test". In: Encyclopedia od Research Design. Thousand Oaks, CA: Sage (2010). p. 1–5.

[27] Doğan M Şimşek O Kurultay Ş. Süt endüstrisinde katkı maddeleri olarak stabilizatörler. Gida. (1996) 21:251–9.

[28] Atasever M. Yoğurt üretiminde bazı stabilizörlerin kullanımı. Van YYU Vet Fak Derg. (2004) 15:1–4.

[29] Lal SND O’Connor CJ Eyres L. Application of emulsifiers/stabilizers in dairy products of high rheology. Adv Colloid Interf Sci. (2006) 123-126:433–7. doi: 10.1016/j.cis.2006.05.009, 16806031

[30] Penna ALB Oliveira MN Tamime AY. Influence of carrageenan and total solids content on the rheological properties of lactic beverage made with yogurt and whey. J Texture Stud. (2003) 34:95–113. doi: 10.1111/j.1745-4603.2003.tb01058.x

[31] Tamime AY Robinson RK. Yoghurt Science and Technology. Boca Raton, Boston, New York, Washington, DC: Woodhead Publishing Ltd (2007). p. 35–41.

[32] Borgeson N Müller S Kishi A. "Food additives". In: Food Additives, Specıalty Chemicals. Menlo Park, CA, USA: SRI intarnational (2002) 70–80.

[33] Sharma BR Naresh L Dhuldhoya NC Merchant SU Merchant UC. Xanthan gum - a boon to food industry. Food Promotion Chronicle. (2006) 1:27–30.

[34] Burey P Bhandarı BR Howes T Gıdley MJ. Hydrocolloid gel particles: formation, characterization, and application. Crit Rev Food Sci Nutr. (2008) 48:361–77. doi: 10.1080/10408390701347801, 18464027

[35] Spreer E. Milk And Dairy Product Technology. Madison Avenue, New York NY, USA: Marcel Dekker, Inc (1998).

[36] Everett DW McLeod RE. Interactions of polysaccharide stabilizers with casein aggregates in stirred skim-milk yoghurt. Int Dairy J. (2005) 15:1175–83. doi: 10.1016/j.idairyj.2004.12.004

[37] McClements DJ. Food Emulsions. Boca Raton, London, New York, Washington, DC: CRC Press (2005). p. 158–65.

[38] Yurdagel Ü. (1983). Xanthan Gum (Xanthan Sakızı). Gıda, 8:15–19. Available online at: https://izlik.org/JA22BK37EP

[39] Anonymous. Xanthan Gum. İzmir, Türkiye: Biokim (2026). Available online at: https://biokimkimya.com/en/gida-katkilari (Accessed August 29, 2026).

[40] Dickinson E. Hydrocolloids at interfaces and the influenceon the properties of dispersed systems. Food Hydrocoll. (2003) 17:25–39. doi: 10.1016/s0268-005x(01)00120-5

[41] Nespolo CR Brandelli A. Characterization of cheeses produced with ovine and caprine milk and microbiological evaluation of processing areas in the dairy plant in Brazil. Int Food Res J. (2012) 19:1713–21.

[42] Pitso S Bester BH. Quality aspects of feta cheese manufactured from mixtures of cow’s milk and goat’s milk. Milk Sci Int Milchwiss. (2000) 55:454–8.

[43] Prinsloo M. Quality Attributes of Feta Cheese Manufactured from Ultrafiltered Bovine Milk. Pretoria, South Africa: University of Pretoria (1997). p. 58–65.

[44] Pexara A Nikolaos Ν Sergelidis D Govaris A. Fate of enterotoxigenic*Staphylococcus aureus* and staphylococcal enterotoxins in feta and Galotyri cheeses. J Dairy Res. (2012) 79:405–13. doi: 10.1017/s0022029912000325 22849852

[45] Vassiliadis A Psoni L Nikolaou S Arvanitis L Tzanetakis N Litopoulou-Tzanetaki E. Changes in microbial populations, kinds of lactic acid bacteria and biochemical characteristics of Greek traditional feta cheese during ripening. Int J Dairy Technol. (2009) 62:39–47. doi: 10.1111/j.1471-0307.2008.00453.x

[46] Koyuncu M. Studies on Preventing body Softening Issue of white cheese by Various Production and Ripening Applications. Van, Türkiye: Van Yüzüncü Yıl Üniversitesi Fen Bilimleri Enstitüsü (2017).

[47] Yerlikaya O. A Study on the Production of white cheese with caper and its Quality Characteristics. İzmir, Türkiye: Ege Üniversitesi Ziraat Fakültesi Süt Teknolojisi ABD (2008).

[48] Çınar K Güleç HA Güneş G Hi̇csasmaz Z. Utilization of retentates for white brined cheese production: comparative evaluation of physico-chemical and sensory properties. J Food Meas Charact. (2024) 18:8397–407. doi: 10.1007/s11694-024-02808-z

[49] Sancak H Sancak YC. Studies on the physical, chemical, microbiological and organoleptical properties of the white pickled cheese consumed in Van. Van Yuzuncu Yil Univ Saglik Bil Derg. (1995) 2:106–13.

[50] Karaca OB. Effects of using Proteolytic and lipolytic Enzyme from Microbiolojical Sources on Ripening and Properties of white cheese. Adana, Türkiye: Çukurova Üniversitesi (2007).

[51] Kırkın C. Preservation of pre-cut white Cheeses by Modified Atmosphere Packaging. İstanbul: İstanbul Teknik Üniversitesi, Fen Bilimleri Enstitüsü, Gıda Mühendisliği ABD, Yüksek Lisans Tezi (2009).

[52] Medes PHO Pereira DA Pires CV Penna CFAM Glória JR Lana ÂMQ . Microbiological and physicochemical quality of raw materials and Cabacinha artisanal cheese: a traditional stretched-curd cheese from Vale do Jequitinhonha, Minas Gerais, Brazil. J Dairy Sci. (2026) 109:7941–55. doi: 10.3168/jds.2026-28395, 42114741

[53] Abdulhadi I Shakak M Doosh K. Study of the physicochemical and microbiological properties of mozzarella cheese coated with edible casings subsidized with green tea. J Appl Sci Nanotechnol. (2023) 3:151–63. doi: 10.53293/jasn.2022.5086.1175

[54] Şener L. Beyaz peynir üretiminde transglutaminaz enzimi kullanım olanakları. Bolu, Türkiye: Abant İzzet Baysal Üniversitesi, Fen Bilimleri Enstitüsü (2012).

[55] Yaşar E. Utilization of Rennet paste in the Production of Turkish white cheese and its Effects on cheese Quality. Ankara, Türkiye: Hacettepe Üniversitesi, Fen Bilimleri Enstitüsü, Gıda Mühendisliği ABD (2011).

[56] Anonymous. Tuz Tüketimi ve Gıda Kaynakları Çalışması -SALTurk II (2012). Ankara, Türkiye: Türk Hipertansiyon ve Böbrek Hastalıkları Derneği (2012).

[57] Gider K. Beyaz peynirlerde tuz geçisini etkileyen bazı Faktörlerin belirlenmesi. Konya, Türkiye: Selçuk Üniversitesi (2006).

[58] Tayar M. Chemical and microbiological changes during the ripening of white pickled cheese (Turkish with English abstract). Gıda: DergiPark (Istanbul University). (1995) 20:2. Available online at: https://izlik.org/JA67LD95ZB

[59] Anifantakis EM. In: Robinson RK Tamime AY, editors. Traditional Feta Cheese, “Feta and Related Cheeses”. England: Ellis Horwood Limited (1991). p. 49–69. doi: 10.1007/978-94-011-3824-6_2

[60] Barać M Šarić Z Vučić T Ignjatović IS Milinčić DD Špirović-Trifunović B . Effect of ripening in brine and vacuum on protein, fatty acid and mineral profiles, and antioxidant potential of reduced-fat white cheese. Food Technol Biotechnol. (2021) 59:44–55. doi: 10.17113/ftb.59.01.21.6891, 34084079 PMC8157088

[61] Erkaya T. Determination of some Quality Characteristics and Bioactivity of Formed Peptides in white Cheeses Produced with Probiotic Cultures during Ripening. Erzurum: Atatürk Üniveritesi Fen Bilimleri Enstitüsü Doktora Tezi (2014).

[62] Ertürkmen P. Isolation of Starter Culture from white cheese and those Cultures their Effects on the cheese Properties. Isparta: Süleyman Demirel Üniversitesi, Fen Bilimleri Enstitüsü, Gıda Mühendisliği ABD, Yüksek Lisans Tezi (2014).

[63] Biçer EB. The impact of curd Cooking Temperature and time in Traditional white cheese Produced in the region of Sivas on cheese Yield, Melting Behaviour and Texturel Properties. Sivas, Türkiye: Cumhuriyet Üniversitesi, Fen Bilimleri Enstitüsü, Gıda Mühendisliği ABD (2014).

[64] Soltani M. Changes in texture profile and mineral content of ultrafiltered white cheese produced using different salt concentrations. Mljekarstvo/MljekarstvoCom. (2023) 74:64–74. doi: 10.15567/mljekarstvo.2024.0105

[65] İlhan G Zeynep G-S Can S Atıf. The effect of modified atmosphere packaging on some properties of various types of cheese (Turkish with English abstract). DergiPark (Istanbul University). (2009)

[66] Gün İ Güzel Seydim Z Seydim AC. The effect of modified atmosphere packaging on some properties of various types of cheese (Turkish with English Abstract). Gida. (2009) 34:309–16. Available online at: https://izlik.org/JA73YZ54RW

[67] Khosrowshahi A Madadlou A Mousavi M Emam-Djomeh Z. Monitoring the chemical and textural changes during ripening of Iranian white cheese made with different concentrations of starter. J Dairy Sci. (2006) 89:3318–25. doi: 10.3168/jds.s0022-0302(06)72368-2, 16899664

[68] Şengül M Erkaya T Fırat N. The comparison of some microbiological properties of kashar cheeses produced from raw and pasteurized milks during the ripening period. Atatürk Üniv Ziraat Fak Derg. (2010) 41:149–56.

[69] Kesenkaş H Dinkçi N Kınık Ö. Properties of Köy cheeses produced in different dairies. Ege Univ Ziraat Fak Derg. (2012) 49:167–73.

[70] Doğruer Y Gürbüz Ü Nizamlıoğlu M. The effects of potassiuın sorbate on the chemical and microbiological quality in white pickled cheese. Vet Bilim Derg. (1996) 12:109–16.

[71] Akın N Aydemir S Koçak C Yıldız MA. Changes of free fatty acid contents and sensory properties of white pickled cheese during ripening. Food Chem. (2003) 80:77–83. doi: 10.1016/s0308-8146(02)00242-x

[72] Urhan G. Ankara’da çeşitli kaynaklardan satın alınan beyaz peynirlerin mikrobiyolojik kalite kontrolü üzerinde araştırmalar. Ankara, Türkiye: Ankara University, Institute of Health Sciences, Department of Pharmaceutical Microbiology (2012).

[73] Tavsanli H Irkin R Kisadere I. The effects of different organic acid treatments on some microflora and pathogen *Listeria monocytogenes* of white brine cheese. Kafkas Univ Vet Fak Derg. (2019) 25:201–207. doi: 10.9775/kvfd.2018.20661

[74] Özcan Y Artık N Aloğlu HŞ. Farklı Starter Kültür Kullanımının Beyaz Peynirlerin Olgunlaşma Süresince Laktoz Değişimlerine Etkisi. Kirklareli Univ Muhendislik Fen Bilim Derg. (2020) 6:71–83. doi: 10.34186/klujes.735777

[75] Yanmaz B. Salt Concentration and the Effects of Waiting time on some Quality Criteria of white cheese. Çanakkale, Türkiye: Çanakkale Onsekiz Mart Üniversitesi, Fen Bilimleri Enstitüsü, Gıda Mühendisliği ABD (2019).

[76] Manolopoulou E Sarantinopoulos P Zoidou E Aktypis A Moschopoulou E Kandarakis I . Evolution of microbial populations during traditional feta cheese manufacture and ripening. Int J Food Microbiol. (2003) 82:153–61. doi: 10.1016/s0168-1605(02)00258-1, 12568755

[77] Altın S Tekinşen OC. Konya ve yöresinde tüketime sunulan salamura Beyaz peynirlerin kalitesi. Vet Bilim Derg. (2002) 18:13–8.

[78] Ceylan ZD Demirkaya AK. Determination of *Listeria monocytogenes* presence and some microbiological properties in brined white cheese purchased from markets in Erzurum. Atatürk Üniv Ziraat Fak Derg. (2007) 38:137–41.

[79] Göncüoğlu M Bilir-Ormancı FS Doğru A. Usage of *Enterococcus faecium* as starter culture in white cheese production. Ankara Univ Vet Fak Derg. (2009) 56:249–54.

[80] Kurşun Ö Kırdar S Kale A Güner A. Burdur’da Tüketime Sunulan Beyaz Salamura Peynirlerin Mikrobiyolojik Kalitesinin Belirlenmesi. Erzurum, Türkiye: Türkiye 10. Gıda Kongresi (2008). p. 817–20.

[81] Uğur A. Muğla halk pazarında satışa sunulan ev yapımı peynirlerin mikrobiyolojik özellikleri. Ekoloji. (2001) 10:3–8.

[82] Eroglu A Doğan M Toker ÖS Yılmaz MT. Classification of kashar cheeses based on their chemical, color and instrumental textural characteristics using principal component and hierarchical cluster analysis. Int J Food Prop. (2015) 18:909–21. doi: 10.1080/10942912.2013.864673

[83] Karaman AD Akalın AS. Improving quality characteristics of reduced and low fat Turkish white cheeses using homogenized cream. LWT. (2013) 50:503–10. doi: 10.1016/j.lwt.2012.08.017

[84] Delgado FJ González-Crespo J Cava R Ramírez R. Proteolysis, texture and colour of a raw goat milk cheese throughout the maturation. Eur Food Res Technol. (2011) 233:483–8. doi: 10.1007/s00217-011-1536-3

[85] Bryant A Ustunol Z Steffe JF. Texture of Cheddar cheese as influenced by fat reduction. J Food Sci. (1995) 60:1216–9. doi: 10.1111/j.1365-2621.1995.tb04559.x

[86] Upreti P McKay LL Metzger LE. Influence of calcium and phosphorus, lactose, and salt-to-moisture ratio on Cheddar cheese quality: changes in residual sugars and water-soluble organic acids during ripening. J Dairy Sci. (2006) 89:429–43. doi: 10.3168/jds.s0022-0302(06)72107-5, 16428613

[87] Aydoğan E Ayrancı VK Salum P Erbay Z. Impact of different milk types and storage period on the quality characteristics of Malatya cheese. Dairy. (2025) 6:30–45. doi: 10.3390/dairy6040030

[88] Akyüz N Şimşek O. İthal ve yerli beyaz peynirlerin duyusal, fiziksel, kimyasal ve mikrobiyolojik özellikleri üzerinde araştırmalar. Gıda. (1986) 11.

