# Supplementary material for: Production of white cheese using the gel brine technique and determination of its physicochemical, color, and microbiological properties using multivariate statistical analyses
Source: Front Nutr. 2026 Oct 21;13:1930095. doi: 10.3389/fnut.2026.1930095 (PMC13731406; doi:10.3389/fnut.2026.1930095)
Supplement: Supplementary file 1 [file Table_1.pdf]

Table S1. Experimental Design of White Cheese Samples Containing Different Stabilizers and Salt Levels During Storage

Sample Code	Stabilizer Type	Stabilizer Level (%)	Salt Level (%)	Storage Times (Day)				
CON_0_2_R1	CONTROL	0	2	1st	15th	30th	60th	90th
CON_0_2_R2	CONTROL	0	2	1st	15th	30th	60th	90th
CON_0_4_R1	CONTROL	0	4	1st	15th	30th	60th	90th
CON_0_4_R2	CONTROL	0	4	1st	15th	30th	60th	90th
CON_0_6_R1	CONTROL	0	6	1st	15th	30th	60th	90th
CON_0_6_R2	CONTROL	0	6	1st	15th	30th	60th	90th
CAR_0.5_2_R1	CARRAGEENAN GUM	0.5	2	1st	15th	30th	60th	90th
CAR_0.5_2_R2	CARRAGEENAN GUM	0.5	2	1st	15th	30th	60th	90th
CAR_0.5_4_R1	CARRAGEENAN GUM	0.5	4	1st	15th	30th	60th	90th
CAR_0.5_4_R2	CARRAGEENAN GUM	0.5	4	1st	15th	30th	60th	90th
CAR_0.5_6_R1	CARRAGEENAN GUM	0.5	6	1st	15th	30th	60th	90th
CAR_0.5_6_R2	CARRAGEENAN GUM	0.5	6	1st	15th	30th	60th	90th
CAR_1.0_2_R1	CARRAGEENAN GUM	1	2	1st	15th	30th	60th	90th
CAR_1.0_2_R2	CARRAGEENAN GUM	1	2	1st	15th	30th	60th	90th
CAR_1.0_4_R1	CARRAGEENAN GUM	1	4	1st	15th	30th	60th	90th
CAR_1.0_4_R2	CARRAGEENAN GUM	1	4	1st	15th	30th	60th	90th
CAR_1.0_6_R1	CARRAGEENAN GUM	1	6	1st	15th	30th	60th	90th
CAR_1.0_6_R2	CARRAGEENAN GUM	1	6	1st	15th	30th	60th	90th
CAR_1.5_2_R1	CARRAGEENAN GUM	1.5	2	1st	15th	30th	60th	90th
CAR_1.5_2_R2	CARRAGEENAN GUM	1.5	2	1st	15th	30th	60th	90th
CAR_1.5_4_R1	CARRAGEENAN GUM	1.5	4	1st	15th	30th	60th	90th
CAR_1.5_4_R2	CARRAGEENAN GUM	1.5	4	1st	15th	30th	60th	90th
CAR_1.5_6_R1	CARRAGEENAN GUM	1.5	6	1st	15th	30th	60th	90th
CAR_1.5_6_R2	CARRAGEENAN GUM	1.5	6	1st	15th	30th	60th	90th
XAN_0.5_2_R1	XANTHAN GUM	0.5	2	1st	15th	30th	60th	90th
XAN_0.5_2_R2	XANTHAN GUM	0.5	2	1st	15th	30th	60th	90th
XAN_0.5_4_R1	XANTHAN GUM	0.5	4	1st	15th	30th	60th	90th
XAN_0.5_4_R2	XANTHAN GUM	0.5	4	1st	15th	30th	60th	90th
XAN_0.5_6_R1	XANTHAN GUM	0.5	6	1st	15th	30th	60th	90th
XAN_0.5_6_R2	XANTHAN GUM	0.5	6	1st	15th	30th	60th	90th
XAN_1.0_2_R1	XANTHAN GUM	1	2	1st	15th	30th	60th	90th
XAN_1.0_2_R2	XANTHAN GUM	1	2	1st	15th	30th	60th	90th
XAN_1.0_4_R1	XANTHAN GUM	1	4	1st	15th	30th	60th	90th
XAN_1.0_4_R2	XANTHAN GUM	1	4	1st	15th	30th	60th	90th
XAN_1.0_6_R1	XANTHAN GUM	1	6	1st	15th	30th	60th	90th
XAN_1.0_6_R2	XANTHAN GUM	1	6	1st	15th	30th	60th	90th
XAN_1.5_2_R1	XANTHAN GUM	1.5	2	1st	15th	30th	60th	90th
XAN_1.5_2_R2	XANTHAN GUM	1.5	2	1st	15th	30th	60th	90th
XAN_1.5_4_R1	XANTHAN GUM	1.5	4	1st	15th	30th	60th	90th
XAN_1.5_4_R2	XANTHAN GUM	1.5	4	1st	15th	30th	60th	90th

XAN_1.5_6_R1	XANTHAN GUM	1.5	6	1st	15th	30th	60th	90th
XAN_1.5_6_R2	XANTHAN GUM	1.5	6	1st	15th	30th	60th	90th
GEL_1.0_2_R1	GELATIN	1	2	1st	15th	30th	60th	90th
GEL_1.0_2_R2	GELATIN	1	2	1st	15th	30th	60th	90th
GEL_1.0_4_R1	GELATIN	1	4	1st	15th	30th	60th	90th
GEL_1.0_4_R2	GELATIN	1	4	1st	15th	30th	60th	90th
GEL_1.0_6_R1	GELATIN	1	6	1st	15th	30th	60th	90th
GEL_1.0_6_R2	GELATIN	1	6	1st	15th	30th	60th	90th
GEL_2.0_2_R1	GELATIN	2	2	1st	15th	30th	60th	90th
GEL_2.0_2_R2	GELATIN	2	2	1st	15th	30th	60th	90th
GEL_2.0_4_R1	GELATIN	2	4	1st	15th	30th	60th	90th
GEL_2.0_4_R2	GELATIN	2	4	1st	15th	30th	60th	90th
GEL_2.0_6_R1	GELATIN	2	6	1st	15th	30th	60th	90th
GEL_2.0_6_R2	GELATIN	2	6	1st	15th	30th	60th	90th
GEL_3.0_2_R1	GELATIN	3	2	1st	15th	30th	60th	90th
GEL_3.0_2_R2	GELATIN	3	2	1st	15th	30th	60th	90th
GEL_3.0_4_R1	GELATIN	3	4	1st	15th	30th	60th	90th
GEL_3.0_4_R2	GELATIN	3	4	1st	15th	30th	60th	90th
GEL_3.0_6_R1	GELATIN	3	6	1st	15th	30th	60th	90th
GEL_3.0_6_R2	GELATIN	3	6	1st	15th	30th	60th	90th
PEC_1.0_2_R1	PECTIN	1	2	1st	15th	30th	60th	90th
PEC_1.0_2_R2	PECTIN	1	2	1st	15th	30th	60th	90th
PEC_1.0_4_R1	PECTIN	1	4	1st	15th	30th	60th	90th
PEC_1.0_4_R2	PECTIN	1	4	1st	15th	30th	60th	90th
PEC_1.0_6_R1	PECTIN	1	6	1st	15th	30th	60th	90th
PEC_1.0_6_R2	PECTIN	1	6	1st	15th	30th	60th	90th
PEC_2.0_2_R1	PECTIN	2	2	1st	15th	30th	60th	90th
PEC_2.0_2_R2	PECTIN	2	2	1st	15th	30th	60th	90th
PEC_2.0_4_R1	PECTIN	2	4	1st	15th	30th	60th	90th
PEC_2.0_4_R2	PECTIN	2	4	1st	15th	30th	60th	90th
PEC_2.0_6_R1	PECTIN	2	6	1st	15th	30th	60th	90th
PEC_2.0_6_R2	PECTIN	2	6	1st	15th	30th	60th	90th
PEC_3.0_2_R1	PECTIN	3	2	1st	15th	30th	60th	90th
PEC_3.0_2_R2	PECTIN	3	2	1st	15th	30th	60th	90th
PEC_3.0_4_R1	PECTIN	3	4	1st	15th	30th	60th	90th
PEC_3.0_4_R2	PECTIN	3	4	1st	15th	30th	60th	90th
PEC_3.0_6_R1	PECTIN	3	6	1st	15th	30th	60th	90th
PEC_3.0_6_R2	PECTIN	3	6	1st	15th	30th	60th	90th

Note: Note: Sample codes indicate the stabilizer type, stabilizer concentration, salt concentration, and replicate number, respectively. For example, CAR_1.0_4_R2 represents carrageenan gum at 1.0% concentration, 4% salt concentration, and the second experimental replicate. R1 and R2 represent the first and second experimental replicates, respectively.
